# Synthesis of structurally diverse 3,4-dihydropyrimidin-2(1*H*)-ones via sequential Biginelli and Passerini reactions

**DOI:** 10.3762/bjoc.13.7

**Published:** 2017-01-09

**Authors:** Andreas C Boukis, Baptiste Monney, Michael A R Meier

**Affiliations:** 1 Karlsruhe Institute of Technology (KIT), Institute of Organic Chemistry, Materialwissenschaftliches Zentrum MZE, Building 30.48, Straße am Forum 7, 76131 Karlsruhe, Germany

**Keywords:** Biginelli reaction, molecular diversity, multicomponent reactions, Passerini reaction, tandem reactions

## Abstract

The Biginelli reaction was combined with the Passerini reaction for the first time in a sequential multicomponent tandem reaction approach. After evaluation of all possible linker components and a suitable solvent system, highly functionalized dihydropyrimidone–α-acyloxycarboxamide compounds were obtained in good to excellent yields. In a first reaction step, different 3,4-dihydropyrimidin-2(1*H*)-one acids were synthesized, isolated and fully characterized. These products were subsequently used in a Passerini reaction utilizing a dichloromethane/dimethyl sulfoxide solvent mixture. By variation of the components in both multicomponent reactions, a large number of structurally diverse compounds could be synthesized. In addition, a one-pot Biginelli–Passerini tandem reaction was demonstrated. All products were carefully characterized via 1D and 2D NMR as well as IR and HRMS.

## Introduction

Multicomponent reactions (MCRs) are fascinating straightforward reactions for the preparation of diversely substituted products starting from three or more precursor molecules, forming products containing atoms/moieties of all precursor components. MCRs are often one-pot reactions with high-atom economy, convergence and efficiency. Generally, one-pot procedures have many advantages compared to multiple-step syntheses [1–3]. One-pot MCRs can shorten reaction times, provide high yields, reduce work-up steps and waste as well as energy consumption and hence lead to more effective and sustainable processes [4–6]. MCRs found numerous applications, i.e., in combinatorial chemistry, target oriented synthesis or polymer science [6–8]. The most important MCRs are the Strecker amino acid synthesis (1850), the Hantzsch dihydropyridine synthesis (1882), the Biginelli dihydropyrimidone synthesis (1891), the Mannich reaction (1912), the Passerini three-component reaction (1921) and the Ugi four-component reaction (1959) [9]. In this work, we used Biginelli and Passerini reactions to synthesize highly functionalized compounds, hence both reactions will be described in detail.

### The Biginelli reaction

**Scheme 1:**
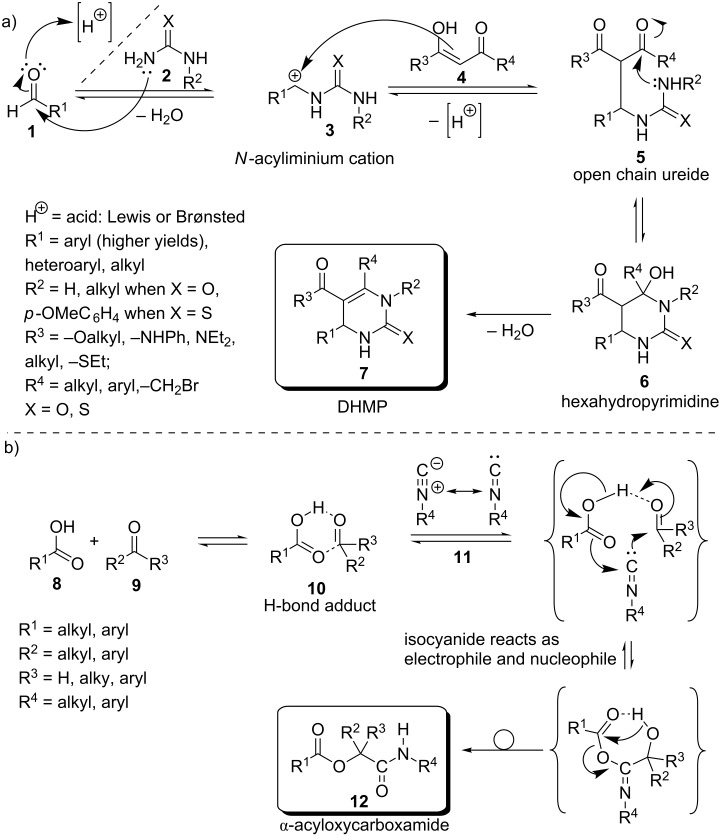
a) Proposed mechanism of the Biginelli reaction according to [6]. b) Proposed mechanism of the Passerini reaction.

The Biginelli reaction is a three-component reaction between an aldehyde (in many cases aromatic aldehydes give much better results than aliphatic ones), a β-keto ester (α-acidic compound) and urea or thiourea (some mono *N*-substituted ureas can also be employed). The Biginelli reaction was discovered in 1891 by the chemist Pietro Biginelli [10]. Later, Biginelli identified the reaction product as a 3,4-dihydropyrimidin-2(1*H*)-one (DHMP) [11]. DHMPs are of great interest due to their pharmaceutic activities (i.e., calcium channel modulation, α_1a_ adrenoceptor-selective antagonists, cancer therapy, anti-HIV alkaloids) [12–15]. The mechanism of the Biginelli cyclocondensation was proposed and investigated by Kappe and is illustrated in Scheme 1a [16]. According to the generally accepted mechanism of the Biginelli reaction, aldehyde **1** is activated by a Lewis- or a Brønsted acid. In the next step, urea/thiourea **2** can serve as a nucleophile and react with the activated carbonyl carbon to form a heminal species. However, under acidic conditions heminals can eliminate water and form an *N*-acyliminium cation **3**. This reactive cation **3** can then react with the nucleophilic α-carbon atom of β-ketoester **4** to an open chain ureide **5**. Subsequent ring closure results in a hexahydropyrimidine intermediate **6**. In the last step, the irreversible elimination of water forms the thermodynamically favored DHMP product **7**. This accepted mechanism was supported by spectroscopic data. However, alternative mechanisms are discussed in the literature [17–18]. In the so called enamine route, urea **2** and the β-ketoester **4** form an enamine in the first reaction step. Subsequently, the enamine reacts with the aldehyde **1** [19]. A third mechanism discussed, is the Knoevenagel type reaction between the aldehyde **1** and β-ketoester **4** followed by a subsequent reaction with urea **2** [20].

### The Passerini reaction

The Passerini reaction was discovered in 1921 by Mario Passerini and is a three-component reaction between a carboxylic acid **8**, a carbonyl compound **9** and an isocyanide **11** [21]. The Passerini reaction works best in non-polar solvents like dichloromethane. The mechanism of the Passerini reaction (Scheme 1b) is proposed to proceed via the formation of a hydrogen bond (H-bond) adduct between carboxylic acid **8** and carbonyl component **9**, resulting in a six-membered cyclic H-bond adduct **10**. Subsequently, isocyanide **11** reacts with **10**, thereby showing a simultaneous nucleophilic and electrophilic reactivity (α-addition). The herein formed seven-membered intermediate has not been isolated, because it immediately undergoes a rearrangement, affording the Passerini α*-*acyloxycarboxamide adduct **12** [18].

### Tandem reactions

Tandem reactions (also known as cascade [22] or domino reactions [23]) are chemical transformations that involve at least two independent reactions utilizing different functional groups with distinct chemical reactivities [24–27]. So far, only a few examples of multicomponent tandem reactions are described in the literature [28–29]. Portlock et al. reported on Petasis–Ugi tandem reactions leading to a product with six different side chains [30–31]. Al-Tel et al. combined the Groebke–Blackburn reaction with either Passerini or Ugi reactions in a sequential one-pot procedure [32]. Furthermore, up to eight components were reacted by the combination of three multicomponent reactions [33]. In 2010, the Ugi reaction and the Ugi–Smiles reaction were combined by Westermann et al. [34]. In addition, the Ugi reaction was used in combination with the Biginelli reaction by Brodsky et al. [35]. In this work, five Biginelli acids were synthesized in 33–83% yields and utilized in a Ugi reaction for the synthesis of six DHMP amides with 21–63% yields. In a similar reaction strategy, Wipf et al. synthesized a library of twelve Biginelli compounds and reacted them with the respective Ugi components under reflux in methanol to yield 30 different DHMP amides in 5–51% yield [36]. Furthermore, the Biginelli reaction has been used in a polymerization process combined with the Hantzsch reaction to from copolycondensates [37]. It is noteworthy that in the literature the term tandem is not always used consistently with the initial definition by Tieze et al. [23].

In this work, the Biginelli reaction was combined in a sequential approach with the Passerini reaction for the first time. Furthermore, both reactions were combined in a one-pot tandem procedure. A general overview of our investigations is illustrated in Supporting Information File 1, Scheme S1. All synthesized substances are displayed in Supporting Information File 1, Figure S1.

## Results and Discussion

**Figure 1:**
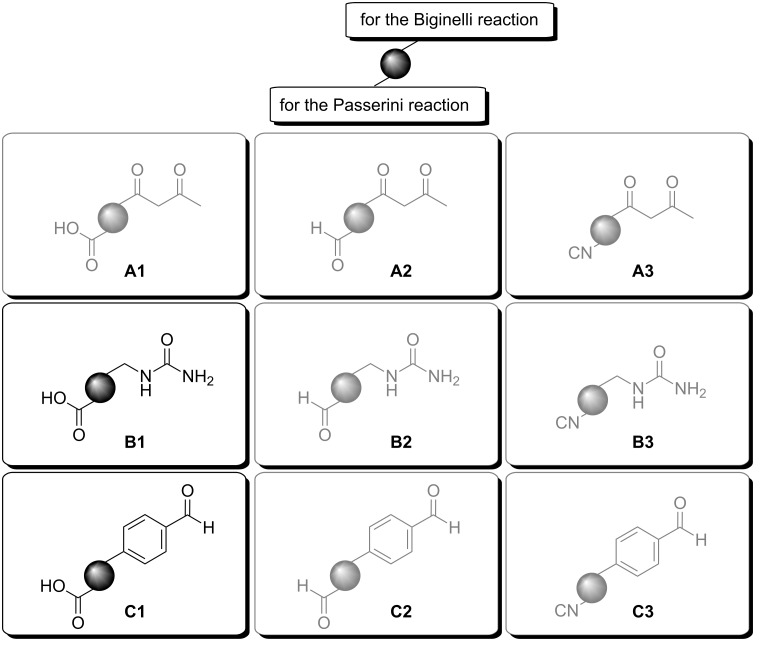
Bifunctional components for the Biginelli–Passerini tandem reaction.

For the Biginelli–Passerini sequential reaction, the Biginelli reaction was performed first, in order to avoid undesired transesterification reactions (of the Passerini product) due to the acidic conditions of the Biginelli reaction [33]. A general challenge, which has to be faced in this context, is the choice of solvent and the selection of bifunctional components (which can interlink both the Biginelli and the Passerini reaction). In the earlier reported Biginelli–Ugi tandem reaction of Wipf et al. [36], methanol was used as solvent. As mentioned previously, the solvent of choice for the Passerini reaction is dichloromethane, providing the highest yields. The DHMP Biginelli products, however, are in most cases very poorly soluble in non-polar solvents. In our investigations, a solvent mixture of dichloromethane with a small amount of dimethyl sulfoxide (polar but aprotic) allowed the successful combination of both chemistries. All possible bifunctional components for the Biginelli–Passerini reaction are represented in Figure 1. Compared to the above mentioned multicomponent tandem approaches, our strategy provides higher yields and makes use of more bifunctional linker components.

Careful evaluation of the bifunctional components allowed a pre-selection: **A3**, **B3** and **C3** in Figure 1 carry an isocyanide functionality, which could hydrolyze under the acidic conditions for the Biginelli reaction [38]. Components **A2**, **B2** and **C2** carry an aldehyde functional group for the Passerini reaction, but this could react on both sides in the Biginelli reaction. Therefore, **A2**, **B2**, **C2** as well as **A3**, **B3**, **C3** were excluded from our investigations. The remaining components **A1**, **B1** and **C1** seemed most promising for our purposes. Hence, we focused on commercially available components with **A1**, **B1** and **C1** like structures, i.e., **C1**: 4-formylbenzoic acid; **B1**: *N*-carbamoylglycine, **A1**: benzyl acetoacetate for the Biginelli reaction and subsequent hydrogenolytic deprotection to the corresponding acid.

The Biginelli reactions were performed in dimethyl sulfoxide at 110 °C in order to remove the water formed in course of the reaction. After a simple washing procedure, the desired DHMP acids **13**–**18** were obtained in 63–93% yield (Table 1). Alternative syntheses for DHMP acids (**13**–**15** and **17**) were described in literature and can be found in Supporting Information File 1. However, our Biginelli approach is simple, utilizes *p*-TSA as a cheap catalyst, provides high yields and can be used for the preparation of various DHMP acids with different bifunctional linkers. Aliphatic aldehydes did not react well under these conditions (even after longer reaction periods of up to six days) and product isolation was not straightforward.

**Table 1:** Biginelli reactions for the preparation of DHMP acids.^a^

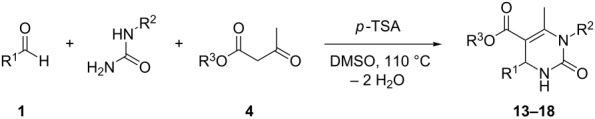					

Entry	R^1^	R^2^	R^3^	Yield [%]	Product

1	Ph	H	Bn	91	**13**
2^b^	Ph	H	H	93	**14**
3	Ph	CH_2_CO_2_H	Et	63	**15**
4	Ph	CH_2_CO_2_H	Bn	78	**16**
5	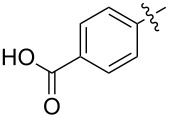	H	Et	90	**17**
6	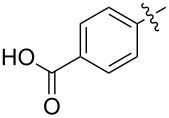	H	Bn	91	**18**

^a^Conditions: 0.10 equiv *p*-TSA, 110 °C 8–48 h in DMSO. ^b^Obtained via hydrogenolytic deprotection of product **13** (entry 1). Conditions: H_2_ (balloon), 10 wt % Pd/C, acetic acid/ethanol (1:3), 50 °C, 15 h.

For the subsequent Passerini reactions, the DHMP acids were dissolved in a mixture of dichloromethane and dimethyl sulfoxide (4:1 → 2:1). After the subsequent addition of the aldehyde and isocyanide components, three days reaction time at room temperature and subsequent purification via column chromatography, the Biginelli–Passerini products **19**–**25** were obtained in 22–99% yield (Table 2).

**Table 2:** Passerini reaction on DHMP acids.^a^

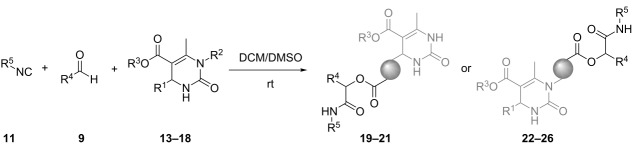								

Entry	DHMP acid	R^1^	R^2^	R^3^	R^4^	R^5^	Yield [%]	Product

1	**17**	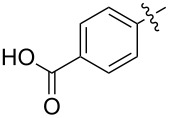	H	Et	C_6_H_13_	*t*-Bu	67	**19**
2	**18**	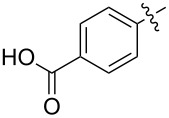	H	Bn	C_6_H_13_	*t*-Bu	22	**20**
3	**17**	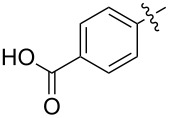	H	Et	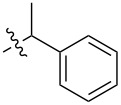	cyclohexyl	98	**21**
4	**15**	Ph	CH_2_COOH	Et	iPr	*t*-Bu	76	**22**
5	**15**	Ph	CH_2_COOH	Et	C_10_H_19_	*t*-Bu	99	**23**
6	**15**	Ph	CH_2_COOH	Et	C_7_H_15_	Bn	76	**24**
7	**15**	Ph	CH_2_COOH	Et	C_7_H_15_	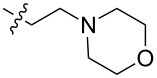	39	**25**
8	**15**	Ph	CH_2_COOH	Et	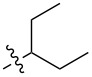	C_5_H_11_	79	**26**

9^b^	**–**	*p-*C_6_H_4_F_c_	CH_2_COOH	Et	*p-*C_6_H_4_F_c_	C_5_H_11_	41	**27**

^a^Conditions: Room temperature, 3 d in DCM. ^b^One pot procedure: Biginelli acid was not isolated.

**Figure 2:**
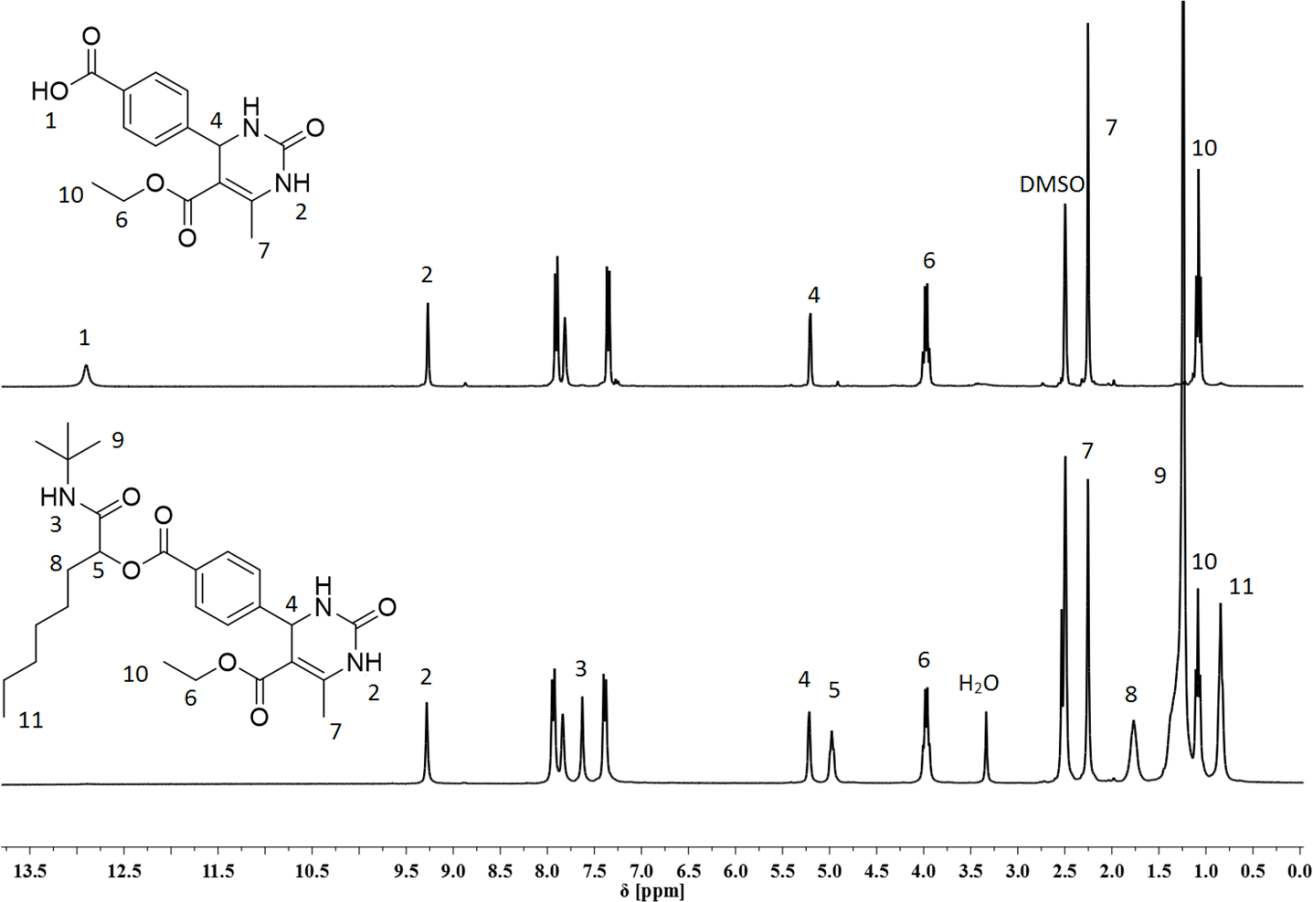
Stacked ^1^H NMR spectra and signal assignment. Top: DHMP acid **17**; bottom: Biginelli–Passerini tandem product **19**.

The lower yield for **25** (39%) might be due to the tertiary amine structure of the morpholinoethyl side chain, requiring a more complex purification. The reaction mixture for the Passerini reaction of DHMP **18** was not completely homogeneous, which might be responsible for the lower yield of **20** (22%). For the other reactions investigated in this work, our Passerini protocol proved to be robust and very effective providing very good to quantitative yields (up to 99% for **23**). In Figure 2, a representative ^1^H NMR comparison between the DHMP acid **17** and the Passerini product **19** is illustrated. The CO_2_*H* proton at 12.9 ppm disappeared after the Passerini reaction, while all other DHMP signals, i.e., the N*H*C at 9.2 ppm, the C*H*NH at 5.2 ppm or the CC*H*_3_ at 2.3 ppm, did not shift. Furthermore, the new characteristic signals for the CC*H*O at 4.9 ppm, the C(C*H*_3_)_3_ at 1.2 ppm and the terminal CH_2_C*H*_3_ methyl group at 0.84 ppm strongly indicate the formation of the respective Biginelli–Passerini product.

As a proof of principle, the Biginelli and Passerini reaction were combined in a one-pot synthesis. In this experiment, the Biginelli reaction was performed with an excess of the aldehyde component (three equivalents) in a minimal amount of dimethyl sulfoxide. After completion of the Biginelli reaction, the crude reaction mixture was cooled to room temperature and diluted with dichloromethane. Subsequently, an isocyanide was added to the mixture enabling the Passerini reaction with the exceeding aldehyde. The resulting one-pot product **27** was obtained in 41% yield after column chromatography (Table 2, entry 9). However, the structural diversity in this approach is limited if compared to the previously described two-step approach (isolation of Biginelli acid) because the same aldehyde component is participating in both MCRs.

**Figure 3:**
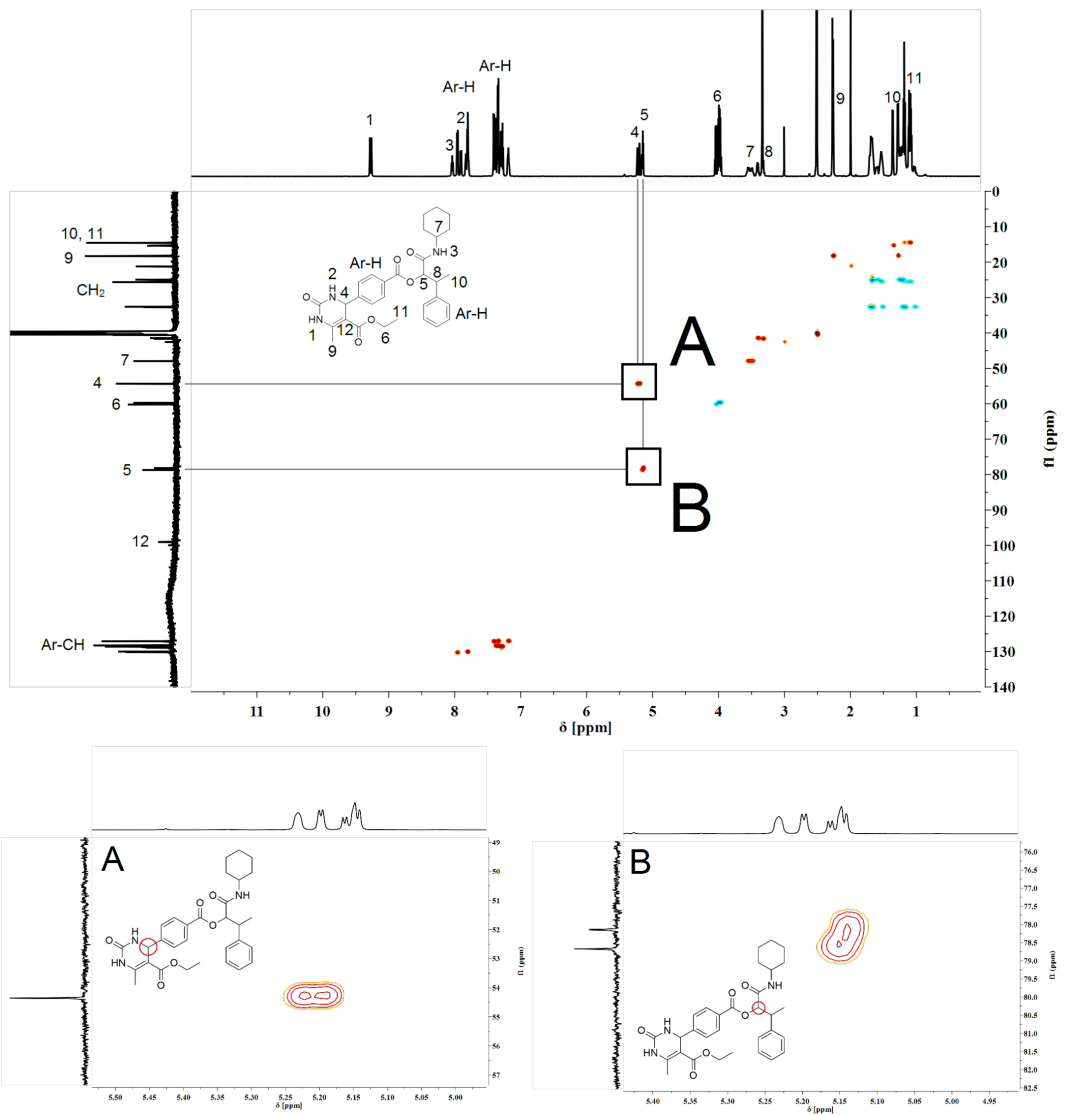
Representative HSQC spectrum of the pure Biginelli–Passerini tandem product **21**, expansions and signal assignment for two asymmetric carbon atoms. A: Diastereomeric signal splitting in ^1^H NMR solely. B: Diastereomeric splitting in both ^1^H and ^13^C NMR, two different species can be identified.

Interestingly, the ^1^H and ^13^C NMR spectra of the chromatographically pure Biginelli–Passerini products displayed a signal splitting for distinct signals (Figure 3).

**Figure 4:**
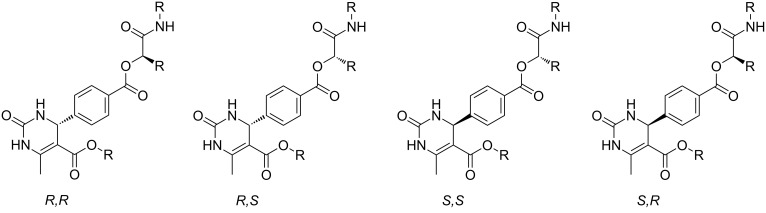
Stereoisomers formed in the Biginelli–Passerini tandem reaction. The homo (*RR*, *SS*) and hetero pairs (*RS*, *SR*) are diastereomers.

A more detailed analysis revealed that most of the split signals were located either next to chiral centres in the molecule or in the six-membered DHMP core. In order to identify the cause of this peak splitting, high temperature NMR experiments at 40 °C, 60 °C and 80 °C were conducted. Even at higher temperatures the peak splitting remained, evidencing that the splitting was not caused by rotational barriers or conformational effects. Furthermore, the splitting was not observed in the DHMP acids **13**–**18** (after the Biginelli reaction, which was performed first). In principle, the Biginelli and Passerini reactions both form a new chiral centre, which was not controlled in our investigations, leading to a racemic mixture (*R* and *S*). After the Passerini reactions, four different stereoisomers (*RR*, *RS*, *SR*, *SS*) are thus obtained. The homo (*RR*, *SS*) and hetero pairs (*RS*, *SR*) are diastereomers with slightly different physical properties. In the context of our experimental NMR data, it is thus fair to assume that the peak splitting is caused by these diastereomers (Figure 4).

## Conclusion

The Biginelli reaction was successfully combined with the Passerini reaction to obtain highly functionalized DHMP heterocyclic products. For this purpose, different DHMP acids were prepared by variation of the components and the bifunctional linker. The DHMP acids were then reacted in a Passerini reaction employing a dichloromethane/dimethyl sulfoxide solvent mixture. The respective Biginelli–Passerini reaction products were in most cases obtained in good to excellent yields. Furthermore, a one-pot Biginelli–Passerini reaction without intermediate work-up was demonstrated. All compounds of this investigation were carefully characterized via NMR (1D and 2D), IR and HRMS. The herein presented strategy is currently under investigation for the preparation of sequence-defined macromolecules [39–40]. Furthermore, the obtained compounds present a rigid, geometrically fixed and highly functionalized DHMP moiety, which could potentially be utilized for covalent organic frameworks and porous materials [41–42].

## Supporting Information

File 1 Experimental section and NMR spectra of all synthesized compounds.

## References

[1] Touré B B, Hall D G (2009). Chem Rev.

[2] D’Souza D M, Müller T J J (2007). Chem Soc Rev.

[3] Dömling A, Ugi I (2000). Angew Chem, Int Ed.

[4] Gu Y (2012). Green Chem.

[5] Ganem B (2009). Acc Chem Res.

[6] Boukis A C, Llevot A, Meier M A R (2016). Macromol Rapid Commun.

[7] Xue H, Zhao Y, Wu H, Wang Z, Yang B, Wei Y, Wang Z, Tao L (2016). J Am Chem Soc.

[8] Hu R, Li W, Tang B Z (2016). Macromol Chem Phys.

[9] Zhu J, Bienaymé H (2006). Multicomponent reactions.

[10] Biginelli P (1891). Ber Dtsch Chem Ges.

[11] Tron G C, Minassi A, Appendino G (2011). Eur J Org Chem.

[12] Kappe C O (2000). Eur J Med Chem.

[13] Ajani O O, Isaac J T, Owoeye T F, Akinsiku A A (2015). Int J Biol Chem.

[14] Selvam T P, James C R, Dniandev P V, Valzita S K (2012). Res Pharm.

[15] Sepehri S, Sanchez H P, Fassihi A (2015). J Pharm Pharm Sci.

[16] Kappe C O (1997). J Org Chem.

[17] Puripat M, Ramozzi R, Hatanaka M, Parasuk W, Parasuk V, Morokuma K (2015). J Org Chem.

[18] Alvim H G O, da Silva Júnior E N, Neto B A D (2014). RSC Adv.

[19] Folkers K, Johnson T B (1933). J Am Chem Soc.

[20] Sweet F, Fissekis J D (1973). J Am Chem Soc.

[21] Passerini M, Simone L (1921). Gazz Chim Ital.

[22] Nicolaou K C, Edmonds D J, Bulger P G (2006). Angew Chem, Int Ed.

[23] Tietze L F (1996). Chem Rev.

[24] Parsons P J, Penkett C S, Shell A J (1996). Chem Rev.

[25] Behr A, Vorholt A J, Ostrowski K A, Seidensticker T (2014). Green Chem.

[26] Zhou J (2010). Chem – Asian J.

[27] Batey R A (2007). J Am Chem Soc.

[28] Brauch S, van Berkel S S, Westermann B (2013). Chem Soc Rev.

[29] Cioc R C, Ruijter E, Orru R V A (2014). Green Chem.

[30] Portlock D E, Ostaszewski R, Naskar D, West L (2003). Tetrahedron Lett.

[31] Portlock D E, Naskar D, West L, Ostaszewski R, Chen J J (2003). Tetrahedron Lett.

[32] Al-Tel T H, Al-Qawasmeh R A, Voelter W (2010). Eur J Org Chem.

[33] Elders N, van der Born D, Hendrickx L J D, Timmer B J J, Krause A, Janssen E, de Kanter F J J, Ruijter E, Orru R V A (2009). Angew Chem, Int Ed.

[34] Brauch S, Gabriel L, Westermann B (2010). Chem Commun.

[35] Fewell S W, Smith C M, Lyon M A, Dumitrescu T P, Wipf P, Day B W, Brodsky J L (2004). J Biol Chem.

[36] Werner S, Turner D M, Lyon M A, Huryn D M, Wipf P (2006). Synlett.

[37] Wu H, Fu C, Zhao Y, Yang B, Wei Y, Wang Z, Tao L (2015). ACS Macro Lett.

[38] Sung K, Chen C-C (2001). Tetrahedron Lett.

[39] Solleder S C, Zengel D, Wetzel K S, Meier M A R (2016). Angew Chem, Int Ed.

[40] Lutz J-F, Lehn J-M, Meijer E W, Matyjaszewski K (2016). Nat Rev Mater.

[41] Feng X, Ding X, Jiang D (2012). Chem Soc Rev.

[42] Muller T, Bräse S (2014). RSC Adv.

