# Supplementary material for: Synthesis of structurally diverse 3,4-dihydropyrimidin-2(1H)-ones via sequential Biginelli and Passerini reactions
Source: Beilstein J Org Chem. 2017 Jan 9;13:54–62. doi: 10.3762/bjoc.13.7 (PMC5238564; doi:10.3762/bjoc.13.7)
Supplement: File 1 — Experimental section and NMR spectra of all synthesized compounds. [file Beilstein_J_Org_Chem-13-54-s001.pdf]

Supporting Information

for

Synthesis of structurally diverse 3,4-dihydropyrimidin-2(1*H*)-ones via sequential Biginelli and Passerini reactions

Andreas C. Boukis, Baptiste Monney and Michael A. R. Meier*

Address: Karlsruhe Institute of Technology (KIT), Institute of Organic Chemistry, Materialwissenschaftliches Zentrum MZE, Building 30.48, Straße am Forum 7, 76131 Karlsruhe, Germany

Email: Michael A. R. Meier - m.a.r.meier@kit.edu

* Corresponding author

Experimental section and NMR spectra of all synthesized compounds

Additional Schemes and Figures S2

General S3

General Procedure (GP 1) for Biginelli reactions S4

General Procedure (GP 2) for Passerini reactions S4

Biginelli compound **13** derived from benzaldehyde, benzylacetoacetate and urea S6

Biginelli acid **14** derived from Biginelli benzylester **13** S9

Biginelli compound **15** derived from *N*-carbamoylglycine, benzaldehyde and ethyl acetoacetate S12

Biginelli compound **16** derived from hydantoic acid, benzaldehyde and benzylacetoacetate S15

Biginelli acid **17** derived from 4-formylbenzoic acid, urea and ethylacetoacetate S19

Biginelli acid **18** derived from 4-formylbenzoic acid, urea and benzyl acetoacetate S21

Passerini product **19** derived from Biginelli acid **17**, *tert*-butyl isocyanide and heptanal S24

Passerini product **20** derived from Biginelli acid **18**, *tert*-butyl isocyanide and heptanal S27

Passerini product **21** derived from Biginelli acid **17**, cyclohexyl isocyanide and 2-phenylpropanal S30

Passerini product **22** derived from Biginelli acid **15**, isobutylaldehyde and *tert*-butyl isocyanide S35

Passerini product **23** derived from Biginelli acid **15**, undec-10-enal and *tert*-butyl isocyanide S38

Passerini compound **24** derived from Biginelli acid **15**, octanal and benzyl isocyanide. S42

Passerini compound **25** derived from Biginelli acid **15**, octanal and 2-morpholinoethyl isocyanide. S46

Passerini compound **26** derived from Biginelli acid **15**, 2-ethylbutanal and 1-pentyl isocyanide S50

Biginelli–Passerini product **27** prepared in one pot procedure from 4-fluorobenzaldehyde, hydantoic acid, ethyl acetoacetate and 1-pentylisocyanide S55

References S59

Additional Schemes and Figures

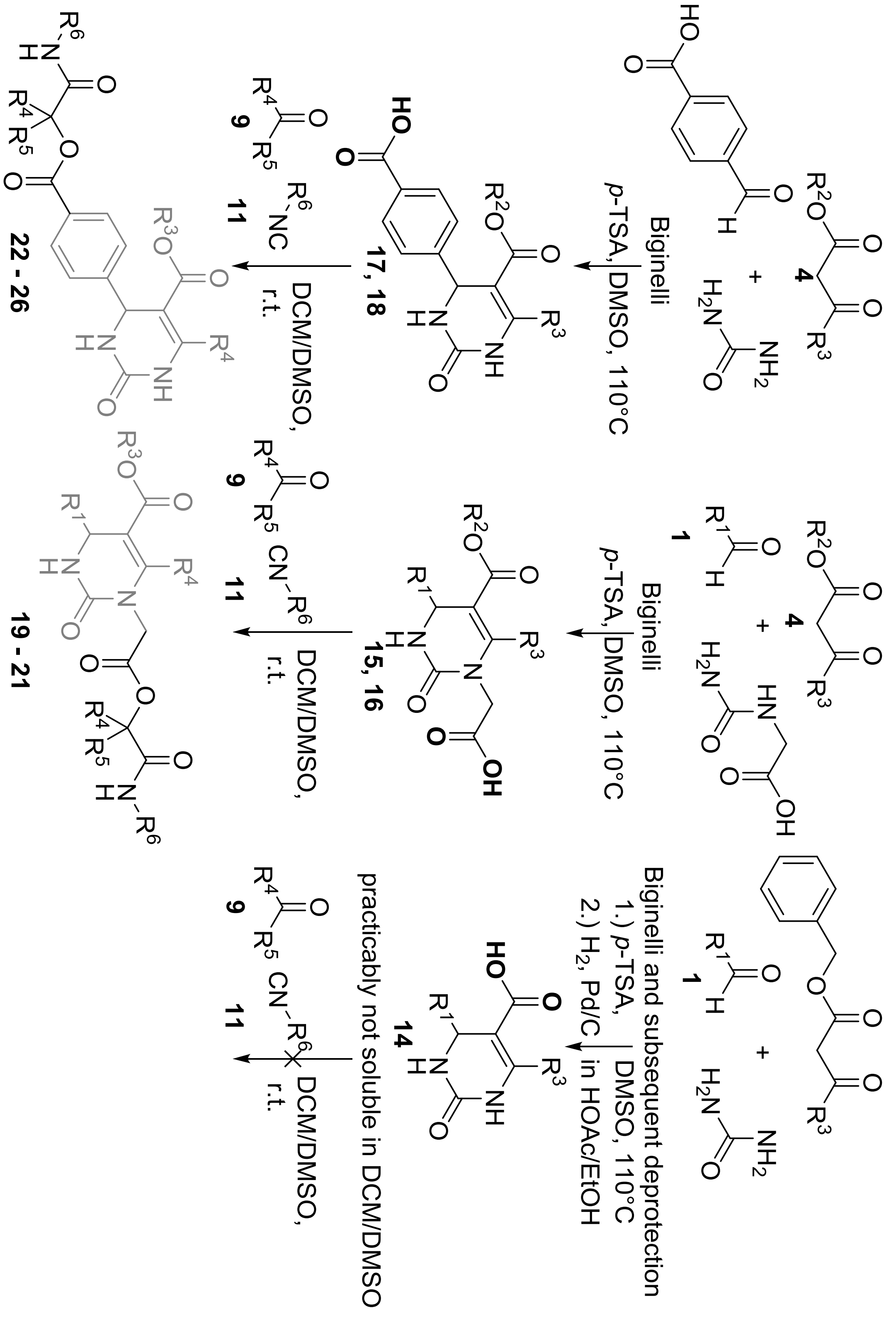

Scheme S1: Biginelli–Passerini tandem reactions, R^1 = aryl; R^2 = Et, Bn; R^3 = Me; R^4 = H; R^5 = alkyl; R^6 = alkyl.

Experimental Section

General. All technical grade solvents were used, if not explicitly described otherwise, without further purification. Ethyl acetate and hexanes were pre-distilled. All commercially available chemicals were used, unless otherwise stated, without further purification and purchased from Sigma-Aldrich. Aldehydes were tested for oxidative contaminations (carboxylic acids) before use via TLC and ^{1}H NMR. Flash column chromatography was performed utilizing Merck SiO_2 60 (230−400 mesh);[1] for TLC analysis, precoated aluminum foils with fluorescence indicator from Merck (TLC Silica gel 60, F_{254}, layer thickness: 0.25 mm) were employed as stationary phase. The spots were firstly visualized by fluorescence quenching under UV-light (λ = 254 nm), fluorescence (λ = 365 nm), and afterwards by staining with Seebach reagent: solution of 2.50 g cerium(IV) sulfate tetrahydrate ($Ce(SO_4)_2 \cdot 4H_2O$), 6.25 g ammonium heptamolybdate tetrahydrate ($(NH_4)_6Mo_7O_{24} \cdot 4H_2O$), 225 mL water and 25.0 mL concentrated sulfuric acid.

^{1}H and ^{13}C NMR spectra were recorded on Bruker Avance DPX spectrometers (Billerica, MA) with a 5 mm dual proton/carbon probe (300 and 400 MHz), on a Bruker Avance III with a 5 mm z-gradient cryogenically cooled probe head (CPTCI, 600 MHz ^{1}H/75.5 MHz) or on a 500 MHz WB Bruker Avance I spectrometer with a proton frequency of 499.97 MHz, ^{13}C frequency of 125.72 MHz on a 8 mm TXI probe head with actively shielded z-gradients (at Θ = 0°) and on a 4 mm triple HCX MAS probe head (at ca. Θ = 65°) at 298 K, regulated with a Bruker VTU-3000. Unless otherwise stated, all spectra were measured at ambient temperature. The chemical shift for ^{1}H NMR spectra are reported in parts per million (ppm) referenced to characteristic solvent signals of partly deuterated solvents, e.g., $CDCl_3$ at 7.26 ppm or the centroid peak of the DMSO-d_6 quintet at 2.50 ppm. ^{13}C NMR spectra are reported in ppm relative to characteristic signals of partly deuterated solvents, e.g., the centroid peak of the $CDCl_3$ triplet at 77.00 ppm or the DMSO-d_6 septet at 39.52 ppm.

Fast-atom-bombardment (FAB) and electron ionization (EI) spectra were recorded utilizing a Finnigan MAT 95 mass spectrometer. Infrared spectra (IR) were recorded on a Bruker Alpha-p instrument applying ATR-technology.

General Procedure (GP 1) for Biginelli reactions. In a flask of appropriate size, finely powdered urea (1.20 equiv) was mixed with the aldehyde (1.00 equiv) and suspended in a small amount dimethyl sulfoxide (1.50–3.00 M for 1.00 equiv). Subsequently, the acetoacetate (1.20 equiv) and 4-methylbenzenesulfonic acid (*p*-TSA, 0.10 equiv) were added. The resulting suspension was stirred at 110 °C for 4–8 h and subsequently for 2–4 d at 80 °C until TLC in ethyl acetate/*c*-hexane indicated complete conversion of the aldehyde. Subsequently, the crude reaction mixture was added dropwise into 100 mL of water while stirring; the resulting slurry was stirred for several hours until a precipitate was formed. The precipitate was filtered off, crushed, washed three times with water, dried and washed three times with cold *n*-hexane/ethyl acetate (2:1). The product was dried under reduced pressure.

General Procedure (GP 2) for Passerini reactions. In a flask of appropriate size, finely powdered Biginelli acid (1.00 equiv) was suspended in dichloromethane and dimethyl sulfoxide was slowly added dropwise while stirring until the compound was completely dissolved. Subsequently, the aldehyde (1.50 equiv) was added and stirred for a few minutes at room temperature. Afterwards, the isocyanide (1.50 equiv) was added to the mixture. The reaction was stirred at room temperature for 3–5 d. TLC in ethanol indicated complete conversion of the Biginelli acid. The crude reaction mixture was dried under reduced pressure and purified *via* column chromatography on silica gel eluting with a gradual solvent mixture of *c*-hexane/ethyl acetate or *c*-hexane/diethyl ether.

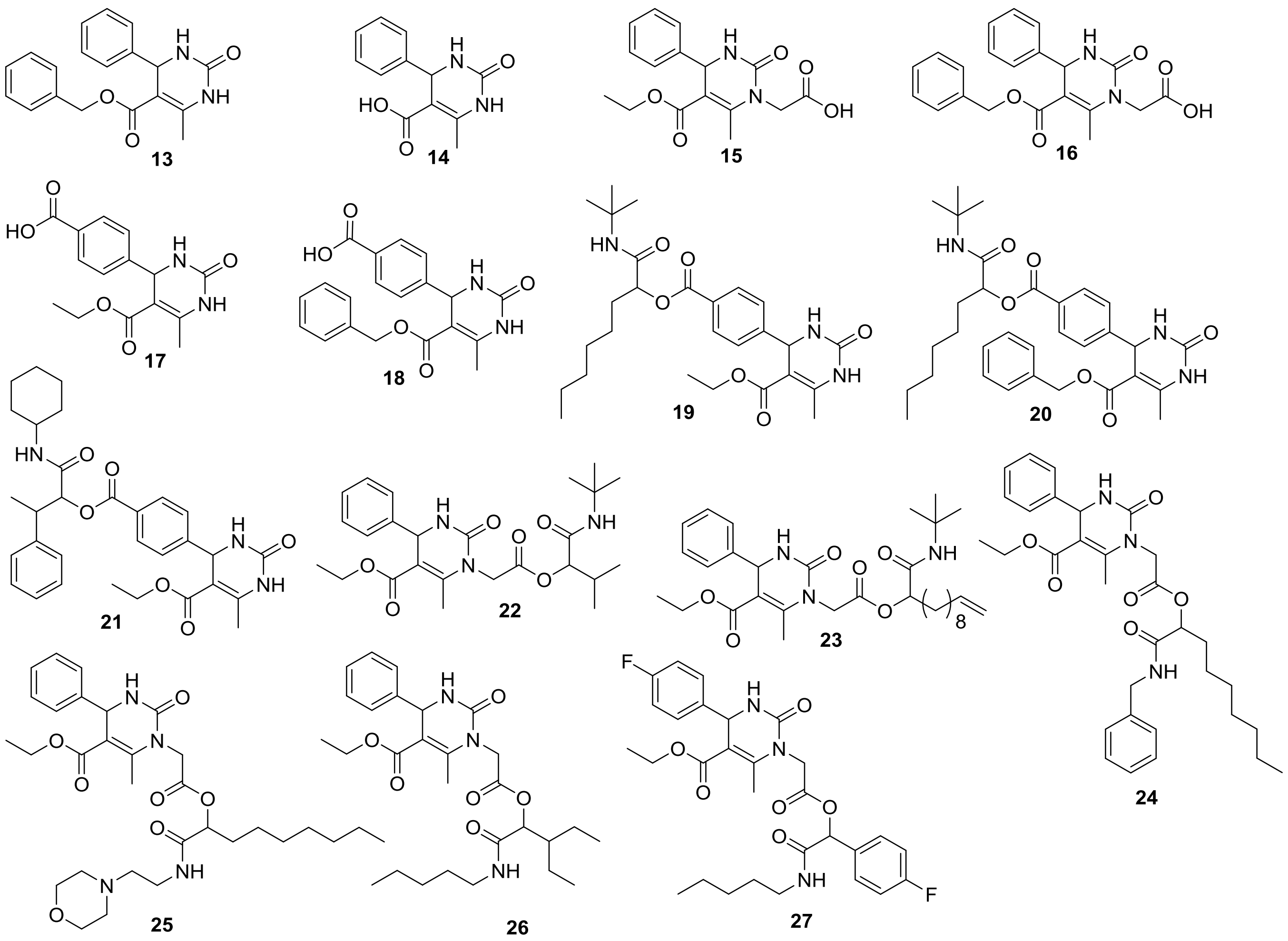

Figure S1: Overview: Synthesized DHMP acids **13–18** and Biginelli–Passerini products **19–27**.

Biginelli compound 13 derived from benzaldehyde, benzyl acetoacetate and urea

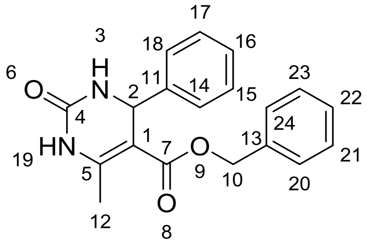

In a tube vial, finely powdered urea (1.50 g, 24.9 mmol, 1.50 equiv) and benzaldehyde (1.76 g, 16.6 mmol, 1.70 mL, 1.00 equiv) were suspended in 3.33 mL dimethyl sulfoxide (5.00 M for 1.00 equiv). Subsequently, benzyl acetoacetate (3.84 g, 20.0 mmol, 3.45 mL) and 4-methylbenzenesulfonic acid (*p*-TSA) (99.9 mg, 833 µmol, 0.05 equiv) were added. The resulting mixture was stirred at 110 °C for 24 h. After TLC indicated complete conversion of benzaldehyde, the crude reaction mixture was added dropwise into 100 mL water while stirring. The suspension was stirred for 3 h until a precipitate was formed. The precipitate was filtered off, crushed, washed with water (3 x 30 mL) and dried. Then, the precipitate was washed with and *n*-hexane (2 x 30 mL) and dried under reduced pressure to yield the Biginelli product **13** as a pale yellow solid (4.86 g, 15.1 mmol, 90.5%).

IR (ATR): ν [cm^{-1}] = 3352.9 (w, ν(N-H)), 3108.6 (br,ν(N-H)), 2975.5 (br, ν(C-H), 1701.2 (s, ν(C=O)), 1684.5 (s, ν(C=O)), 1634.3 (s), 1493.8 (w), 1453.9 (m), 1421.1 (m), 1376.4 (m), 1320.2 (m), 1292.4 (m), 1221.1 (vs), 1137.0 (w), 1082.1 (vs), 1025.1 (m), 963.5 (w), 791.7 (w), 750.7 (w), 720.1 (m), 699.0 (s), 660.3 (m), 612.5 (w), 523.0 (w), 488.3 (m), 385.9 (w).

^{1}H NMR (300 MHz, DMSO-d_6): δ [ppm] = 9.28 (s, 1 H, NH^{19}), 7.77 (s, 1 H, NH^{3}), 7.36 – 7.11 (m, 10 H, CH_{Ar}), 5.18 (d, J = 2.7 Hz, 1 H, CH^{2}), AB-signal (δ A = 5.05, δ B = 5.00, J_{AB} = 13.5 Hz, $CH_2{}^{10}$), 2.28 (s, 3 H, $CH_3{}^{12}$).

^{13}C NMR (101 MHz, DMSO-d_6): δ [ppm] = 165.10 (s, CO_2R^{7}), 152.03 (s, CO^{4}), 149.31 (s, C^{5}), 144.68 (s, $C_{Ar}{}^{11}$), 136.54 (s, $C_{Ar}{}^{13}$), 128.46 (s, CH_{Ar}), 128.30 (s, CH_{Ar}), 127.74 (s, CH_{Ar}), 127.57 (s, CH_{Ar}), 127.35 (s, CH_{Ar}), 126.33 (s, CH_{Ar}), 98.76 (s, C^{1}), 64.84 (s, $CH_2{}^{10}$), 53.96 (s, CH^{2}), 17.89 (s, $CH_3{}^{12}$).

FAB – MS [*m/z*] (relative intensity): 323.3 (100%) $[M + H]^+$, 245.1 (30%) $[M – C_7H_7O]^+$, 231.0 (35%) $[M – C_7H_7]^+$, 215.0 (20%) $[M – C_7H_7]^+$.

HRMS – FAB [*m/z*]: $[M + H]^+$ calculated for $^{12}C_{19}{}^{1}H_{19}{}^{16}O_3{}^{14}N_2$, 321.1390; found, 323.1388; Δ = 0.19 mmu.

Spectral data is in accordance to those reported by Arab-Ameri *et al.*[2]

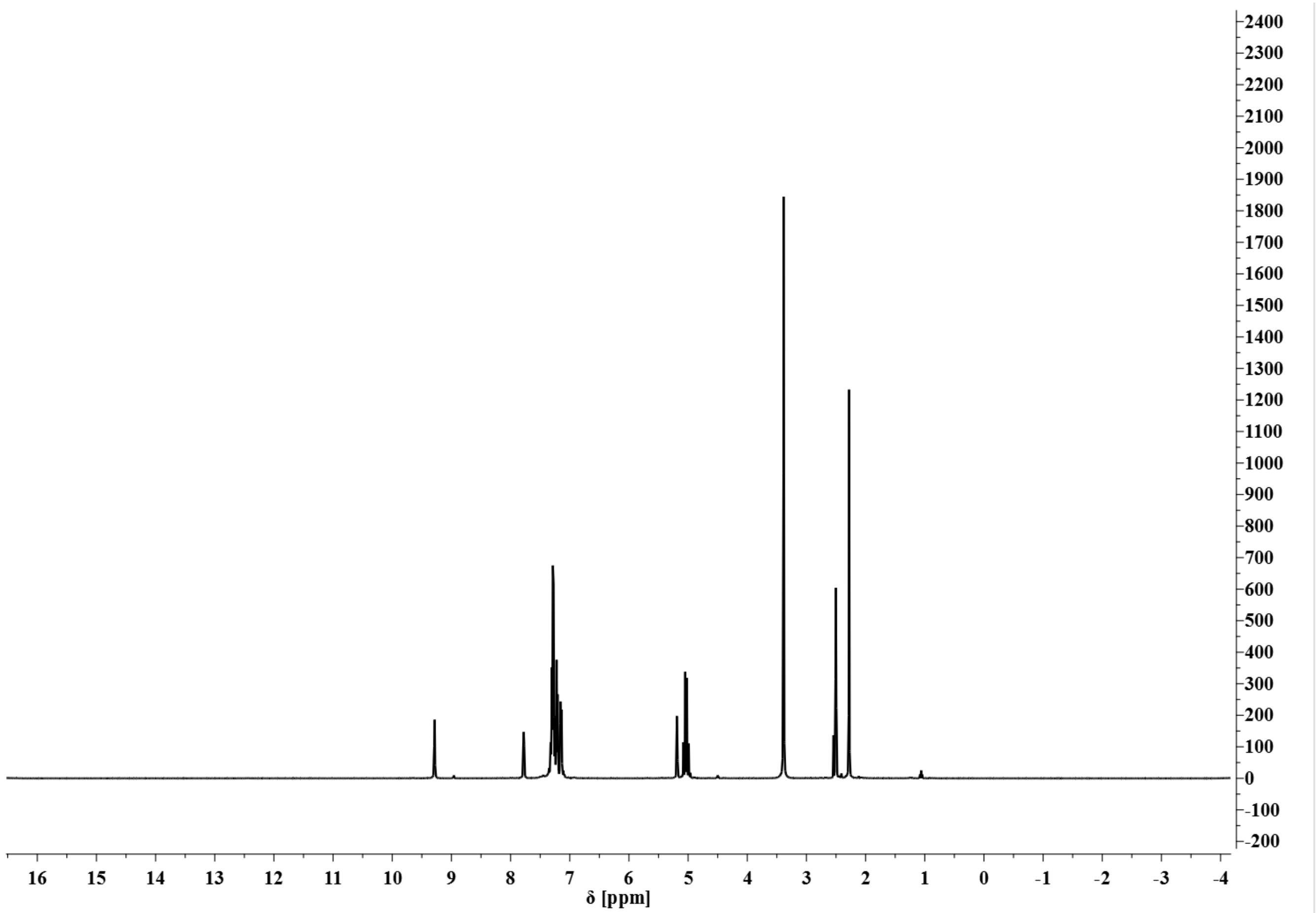
δ [ppm]

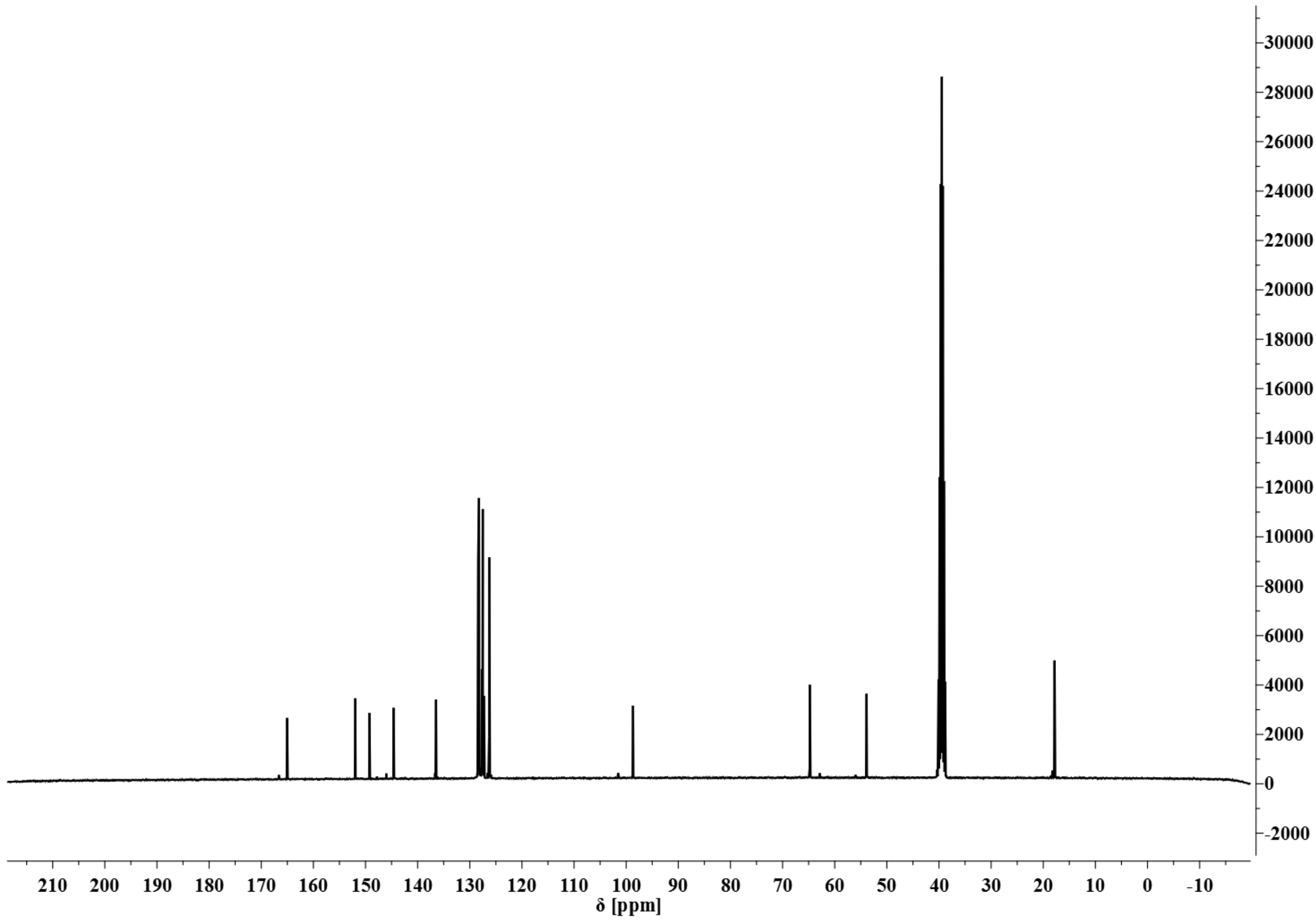
δ [ppm]

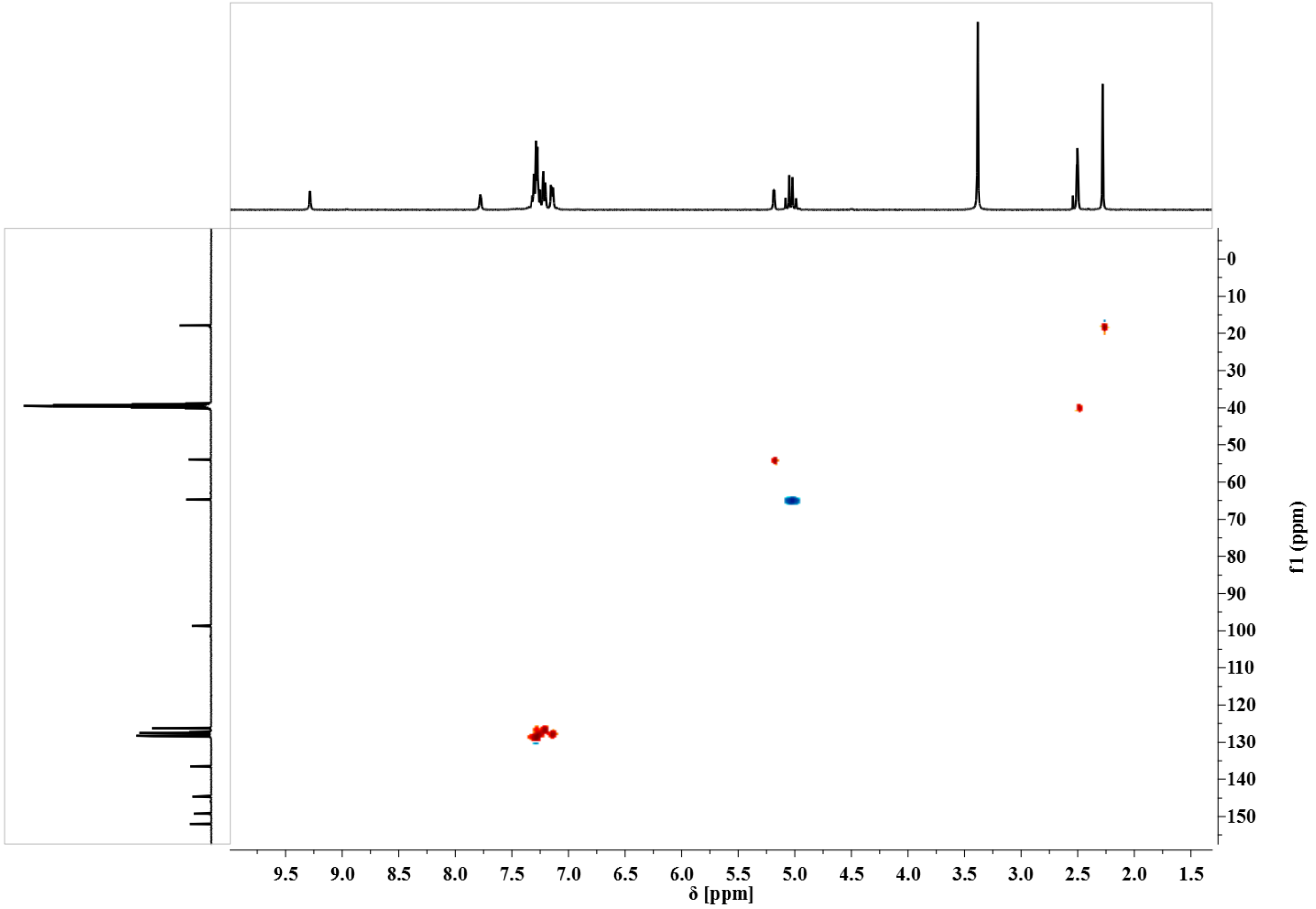

0
10
20
30
40
50
60
70
80
90
100
110
120
130
140
150
f1 (ppm)
9.5 9.0 8.5 8.0 7.5 7.0 6.5 6.0 5.5 5.0 4.5 4.0 3.5 3.0 2.5 2.0 1.5
δ [ppm]

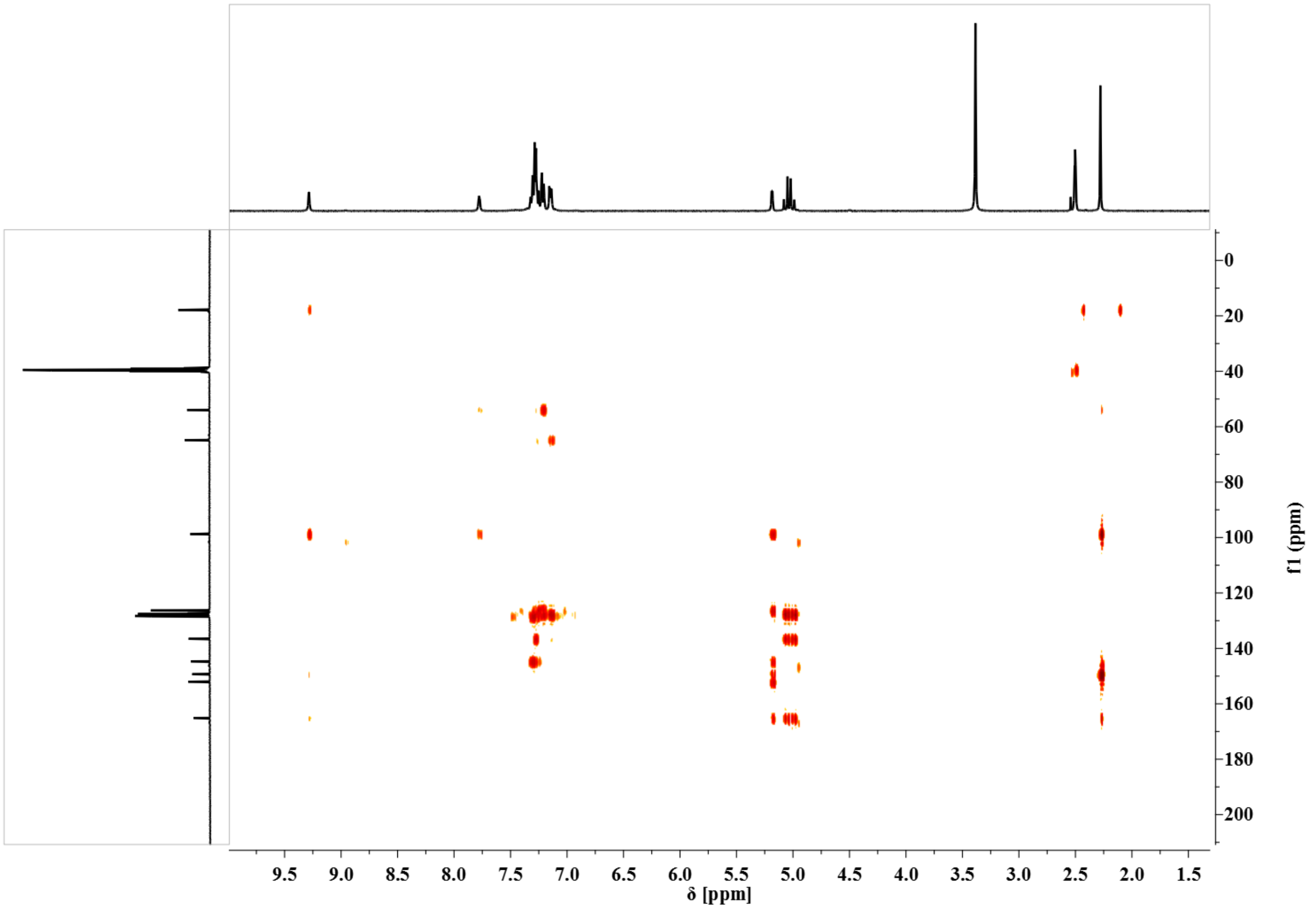

0
20
40
60
80
100
120
140
160
180
200
f1 (ppm)
9.5 9.0 8.5 8.0 7.5 7.0 6.5 6.0 5.5 5.0 4.5 4.0 3.5 3.0 2.5 2.0 1.5
δ [ppm]

Biginelli acid 14 derived from Biginelli-benzyl ester 13

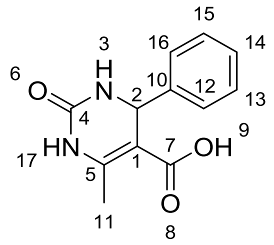

In a tube vial equipped with a magnetic stir bar, the Biginelli-benzyl ester **13** (800 mg, 2.48 mmol, 1.00 equiv) was dissolved in 8.00 mL acetic acid/ethanol (1:3). Subsequently, palladium on activated charcoal (10 wt % Pd, 80.0 mg) was added to the solution and the vial was placed inside an autoclave. Hydrogen gas (20 bar) was applied and the reaction was stirred for 15 h at 50 °C. The crude reaction mixture was concentrated under reduced pressure and stirred with 10 mL 1 M sodium hydroxide solution for 20 min. The suspension was filtered and the yellow filtrate was acidified with hydrochloric acid (pH 1). The white precipitate was filtered off, washed with water (2 × 30 mL), diethyl ether (3 × 50 mL) and was subsequently dried under reduced pressure: The Biginelli acid **14** was obtained as a colorless solid (533 mg, 2.29 mmol, 92.5%).

IR (ATR): ν [cm^{-1}] = 3214,7 (br, ν(N-H)), 3087.0 (br, ν(N-H)), 2975.3 (w, ν(C-H), 1700.7 (s, ν(C=O)), 1643.2 (s, ν(C=O)), 1477.9 (m), 1421.7 (m), 1381.7 (w), 1325.1 (m), 1294.5 (w), 1266.7 (w), 1227.9 (vs), 1217.9 (vs), 1106.6 (w), 889.1 (w), 834.0 (w),753.3 (m), 692.1 (m), 651.8 (s), 613.9 (s), 565.5 (m), 522.6 (w), 483.3 (m), 393.4 (w).

^{1}H NMR (300 MHz, DMSO-d_6): δ [ppm] = 11.80 (br s, 1 H, CO_2H^{9}), 9.09 (s, 1 H, NH^{17}), 7.68 (s, 1 H, NH^{3}), 7.53 – 7.00 (m, 5 H, $CH_{Ar}^{12\text{-}16}$), 5.12 (d, J = 3.0 Hz, 2 H, CH^{2}), 2.24 (s, 3 H, CH_3^{11}).

^{13}C NMR (101 MHz, DMSO-d_6): δ [ppm] = 167.20 (s, CO_2H^{7}), 152.46 (s, CO^{4}), 147.78 (s, C^{5}), 144.85 (s, C_{Ar}^{10}), 128.40 (s, $CH_{Ar}^{12,16}$), 127.20 (s, CH_{Ar}^{14}), 126.28 (s, $CH_{Ar}^{13,15}$), 99.85 (s, C^{1}), 54.00 (s, CH^{2}), 17.79 (s, CH_3^{11}).

FAB – MS [m/z] (relative intensity): 233.0 (40%) $[M + H]^+$, 155.0 (100%) $[M - C_6H_5]^+$.

HRMS – FAB [m/z]: $[M + H]^+$ calculated for $^{12}C_{12}{}^{1}H_{13}{}^{16}O_3{}^{14}N_2$, 233.0921; found, 233.0922; Δ = 0.17 mmu.

^{1}H NMR data is in accordance to those reported by Kappe *et al.*[3]

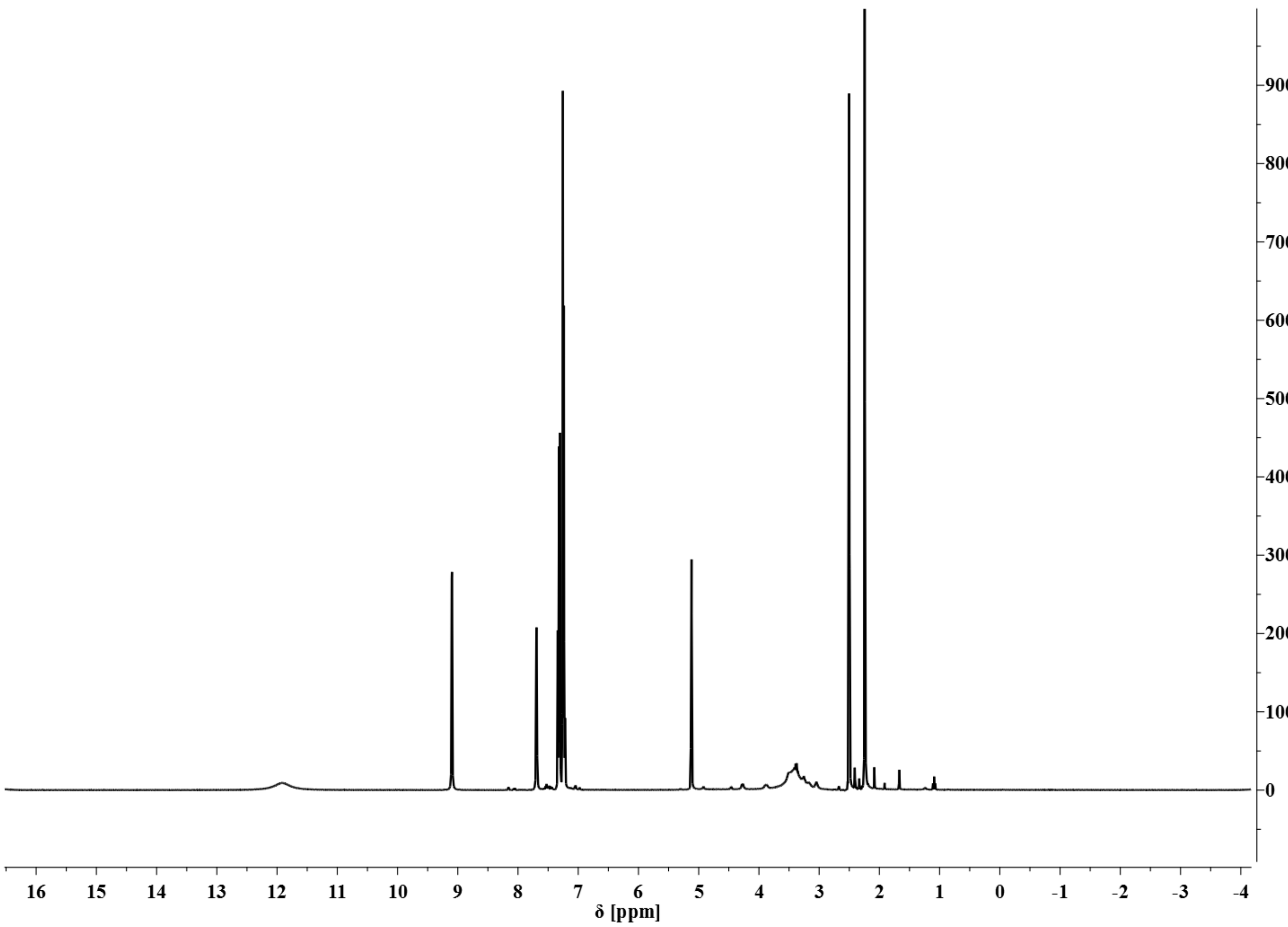
δ [ppm]

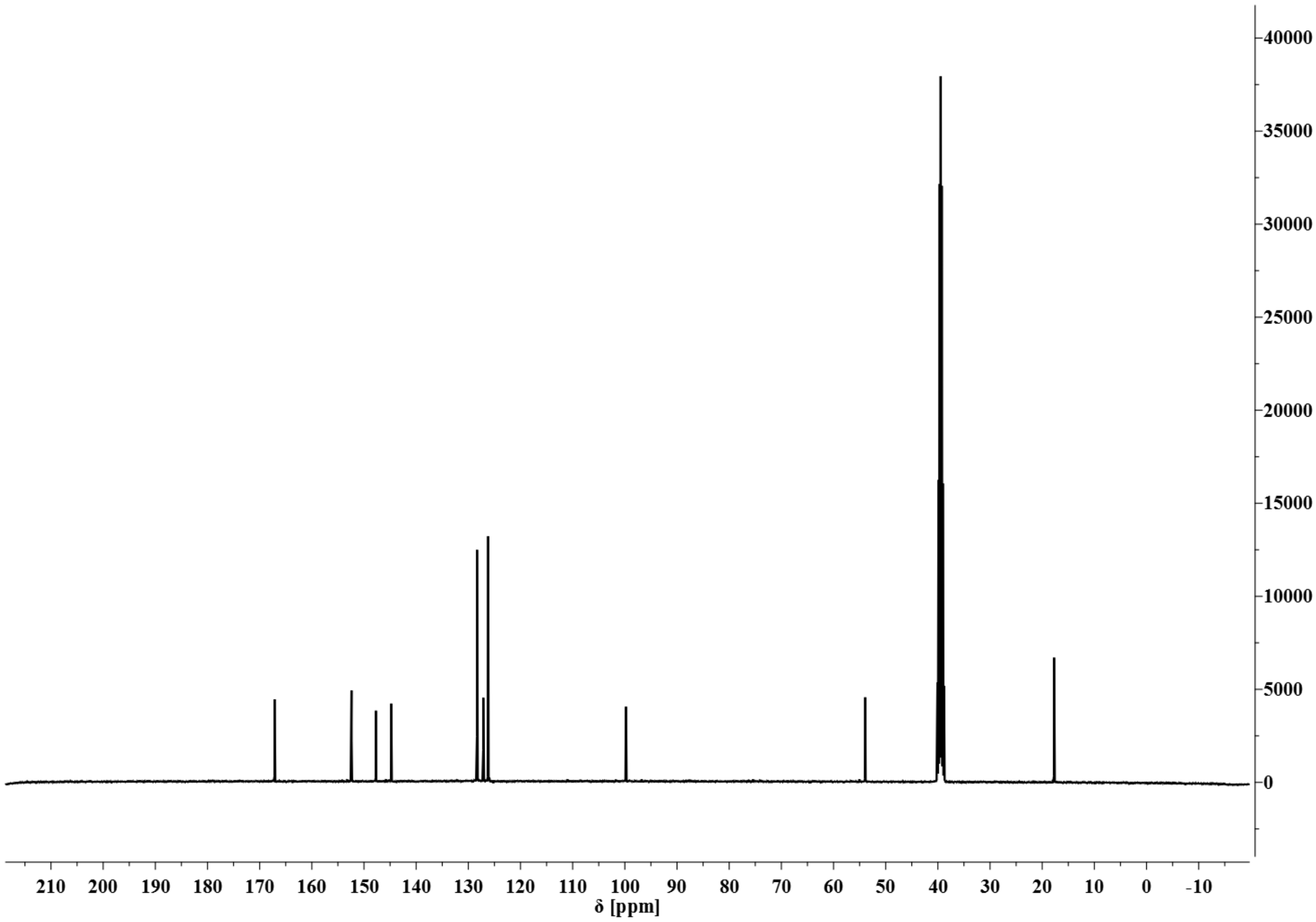
δ [ppm]

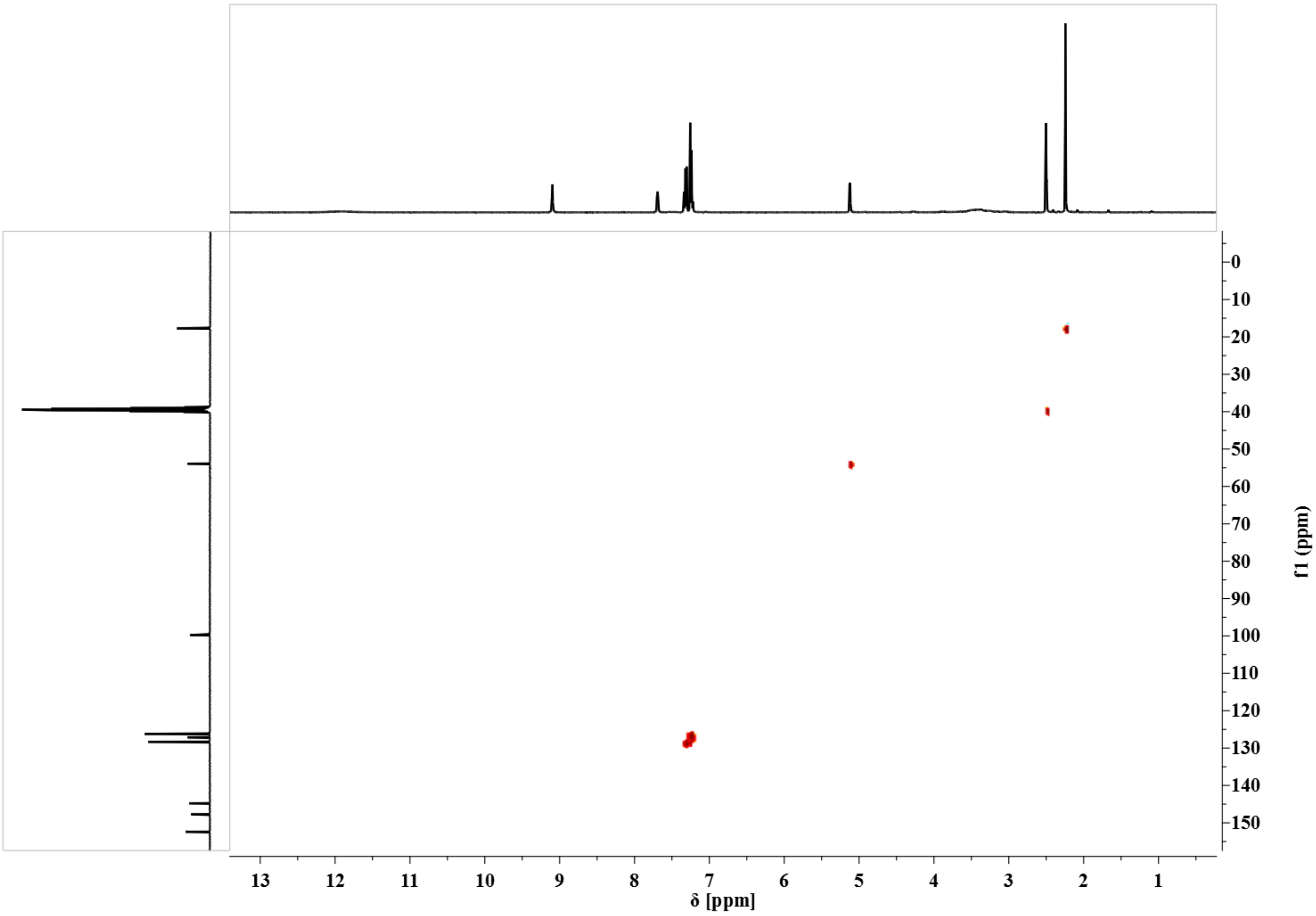

0
10
20
30
40
50
60
70
80
90
100
110
120
130
140
150
f1 (ppm)
13
12
11
10
9
8
7
6
5
4
3
2
1
δ [ppm]

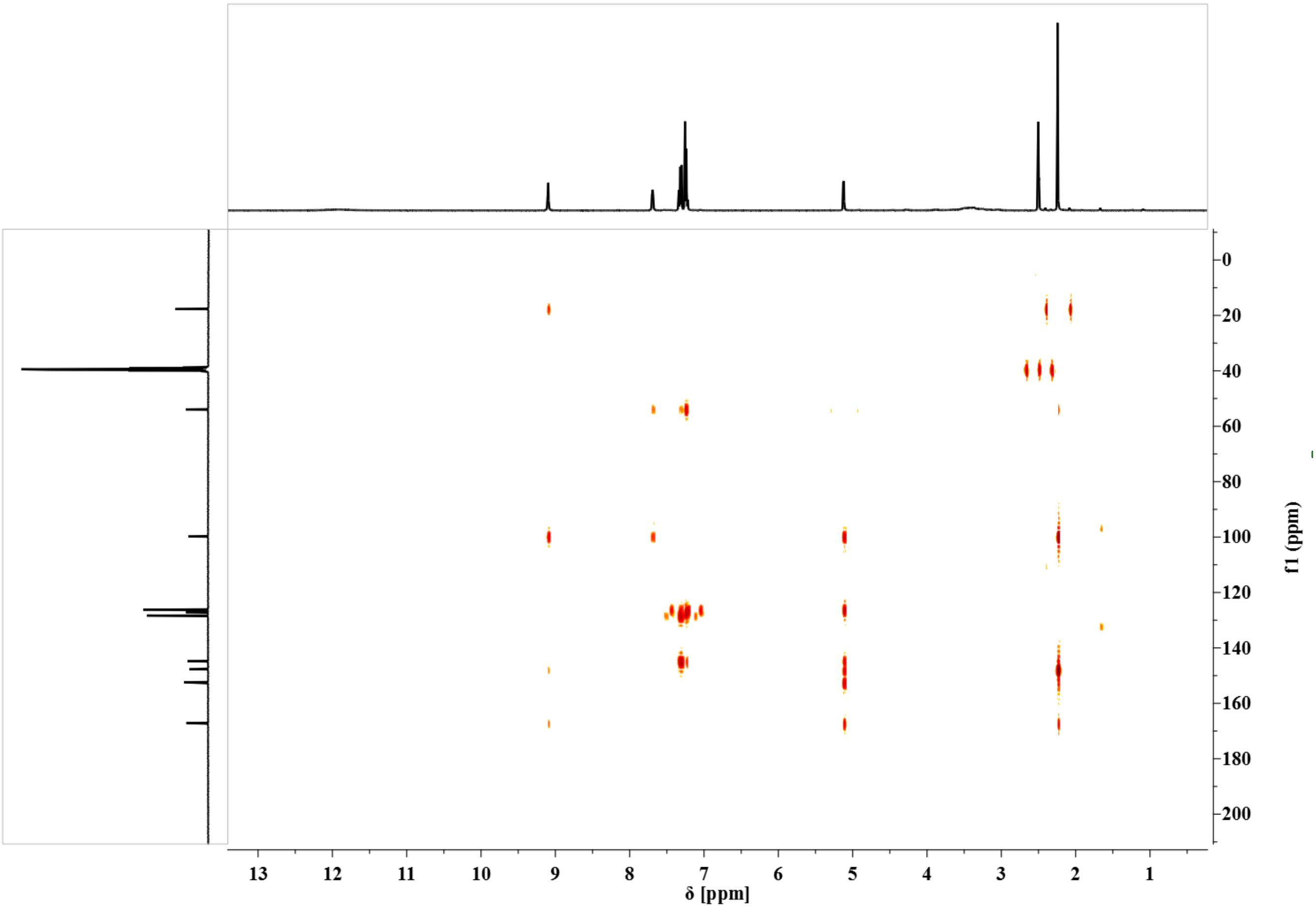

0
20
40
60
80
100
120
140
160
180
200
f1 (ppm)
13
12
11
10
9
8
7
6
5
4
3
2
1
δ [ppm]

Biginelli compound 15 derived from *N*-carbamoylglycine, benzaldehyde and ethyl acetoacetate

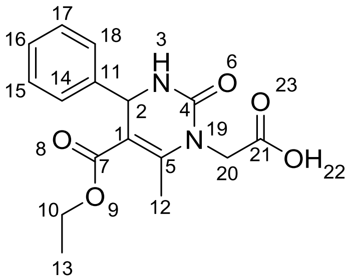

In a 50 mL round-bottomed flask, finely powdered *N*-carbamoylglycine (1.00 g, 8.46 mmol, 1.20 equiv) and benzaldehyde (748 mg, 7.06 mmol, 1.00 equiv) were suspended in 2.50 mL acetic acid/ethanol (3:1) (2.80 M for 1.00 equiv). Ethyl acetoacetate (1.10 g, 8.46 mmol, 1.08 mL, 1.20 equiv) and 4-methylbenzenesulfonic acid (*p*-TSA, 84.7 mg, 706 µmol, 0.10 equiv) were then added. The resulting suspension was stirred at 40 °C for 1 h and then slowly heated to 80 °C and stirred for 4 h to obtain a yellow solution. After TLC indicated complete conversion of benzaldehyde the crude reaction mixture was added dropwise into 100 mL of water while stirring. The slurry was stirred for 1 h until a precipitate was formed, which was filtered off, crushed, washed with water (3 × 20 mL), dried and washed with *n*-hexane (3 × 20 mL). The crude product was dried under reduced pressure yielding Biginelli product **15** as a colorless solid (1.42 g, 5.33 mmol, 63.1%).

IR (ATR): ν [cm^{-1}] = 3290.5 (br , ν(N-H)), 2979.6 (w, ν(C-H), 1725.4 (s, ν(C=O)), 1701.0 (s, ν(C=O)), 1631.2 (s, ν(C=O)), 1453.2 (m), 1402.1 (m), 1364.6 (w), 1311.5 (w), 1279.6 (w), 1233.7 (m), 1213.9 (m), 1181.0 (s), 1120.9 (m), 1058.7 (m), 945.1 (m), 830.0 (m), 783.9 (m), 747.3 (m), 701.6 (s), 679.9 (m), 623.3 (s), 522.3 (m), 483.3 (m), 419.1 (w).

^{1}H NMR (300 MHz, DMSO-d_6): δ [ppm] = 12.90 (s, 1 H, CO_2H^{22}), 8.06 (d, J = 3.4 Hz, 1 H, NH^3), 7.40 – 7.17 (m, 5 H, $CH_{Ar}^{14\text{-}18}$), 5.19 (d, J = 3.1 Hz, 1 H, CH^2), AB-signal (δ A = 4.49, δ B = 4.35, J_{AB} = 18.0 Hz, CH_2^{20}), 4.03 (q, J = 7.1 Hz, 2 H, CH_2^{10}), 2.40 (s, 3 H, CH_3^{12}), 1.10 (t, J = 7.1 Hz, 3 H, CH_3^{13}).

^{13}C NMR (100 MHz, DMSO-d_6): δ [ppm] = 171.06 (s, CO_2H^{21}), 165.57 (s, CO_2R^7), 152.33 (s, CO^4), 149.00 (s, C^5), 144.18 (s, C_{Ar}^{11}), 128.39 (s, $CH_{Ar}^{14,18}$), 127.43 (s, CH_{Ar}^{16}), 126.54 (s, $CH_{Ar}^{15,17}$), 102.92 (s, C^1), 59.66 (s, CH_2^{10}), 53.13 (s, CH^2), 43.95 (s, CH_2^{20}), 15.64 (s, CH_3^{12}), 14.01 (s, CH_3^{13}).

FAB – MS [*m/z*] (relative intensity): 319.1 (100%) $[M + H]^+$, 241.0 (25%) $[M - C_6H_5]^+$.

HRMS – FAB [*m/z*]: $[M + H]^+$ calculated for $^{12}C_{16}{}^{1}H_{19}{}^{16}O_5{}^{14}N_2$, 319.1288; found, 319.1290; Δ = 0.15 mmu.

NMR Spectral data is in accordance to those reported by Ryabukhin *et al.*[4]

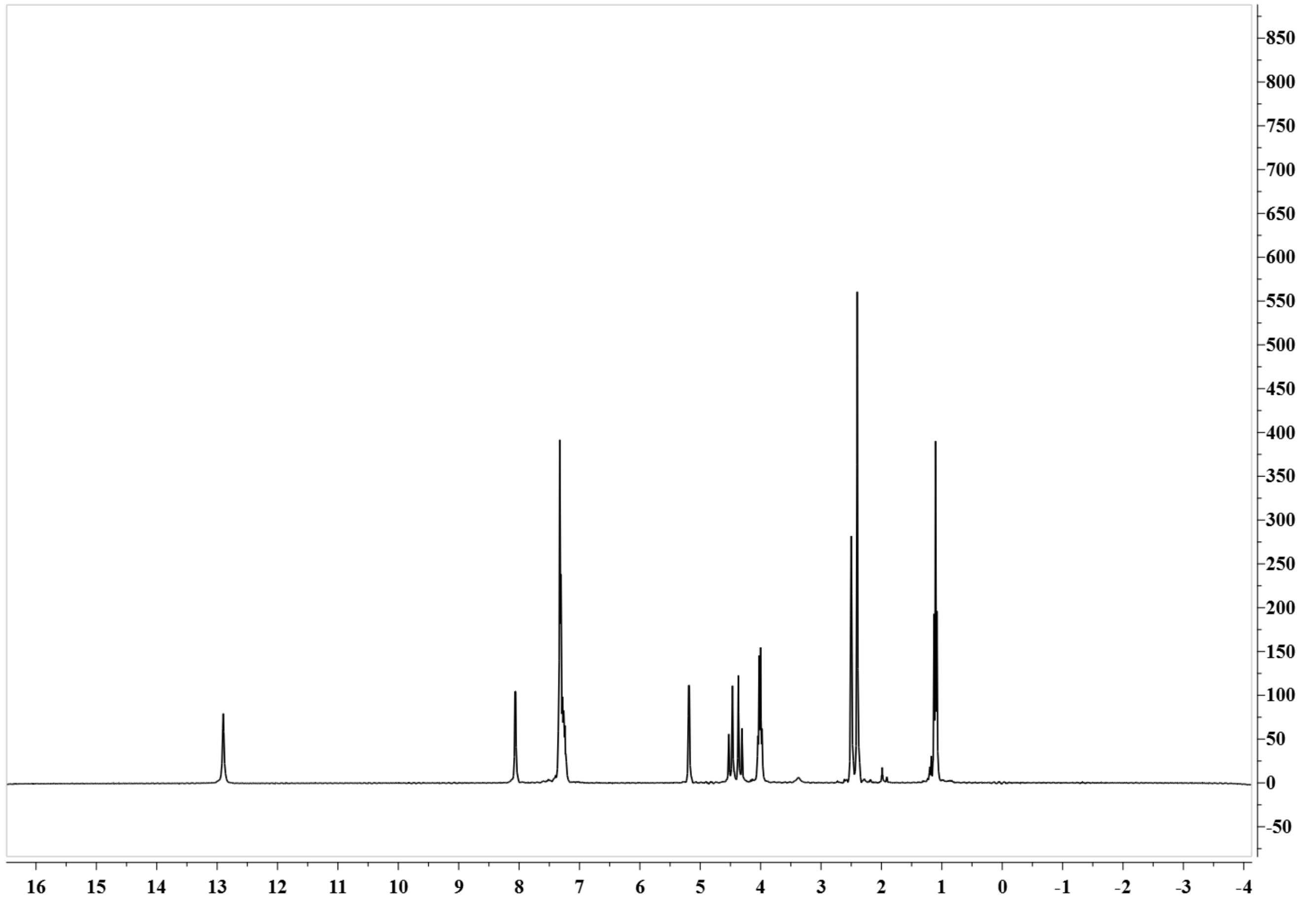
δ [ppm]

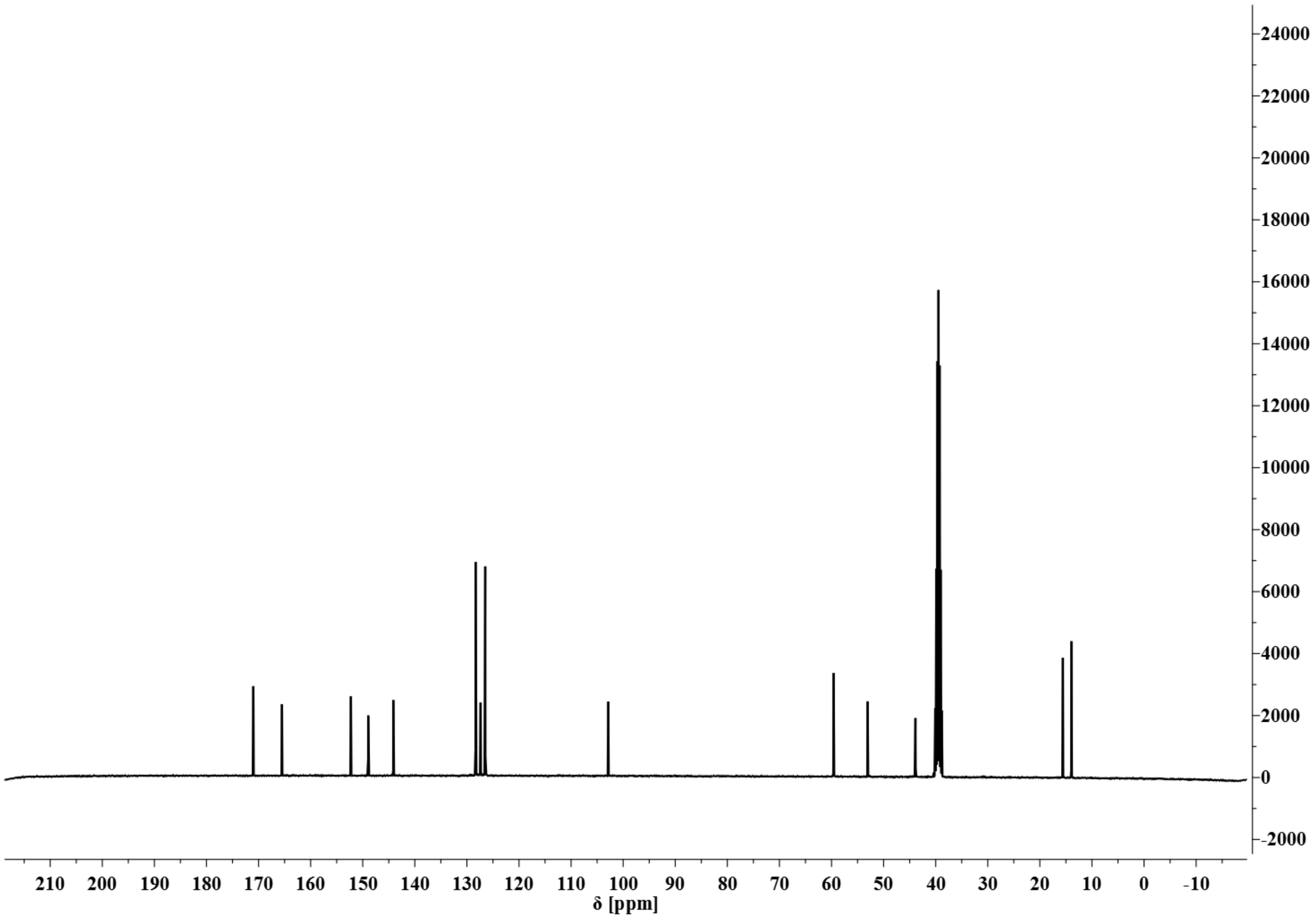
δ [ppm]

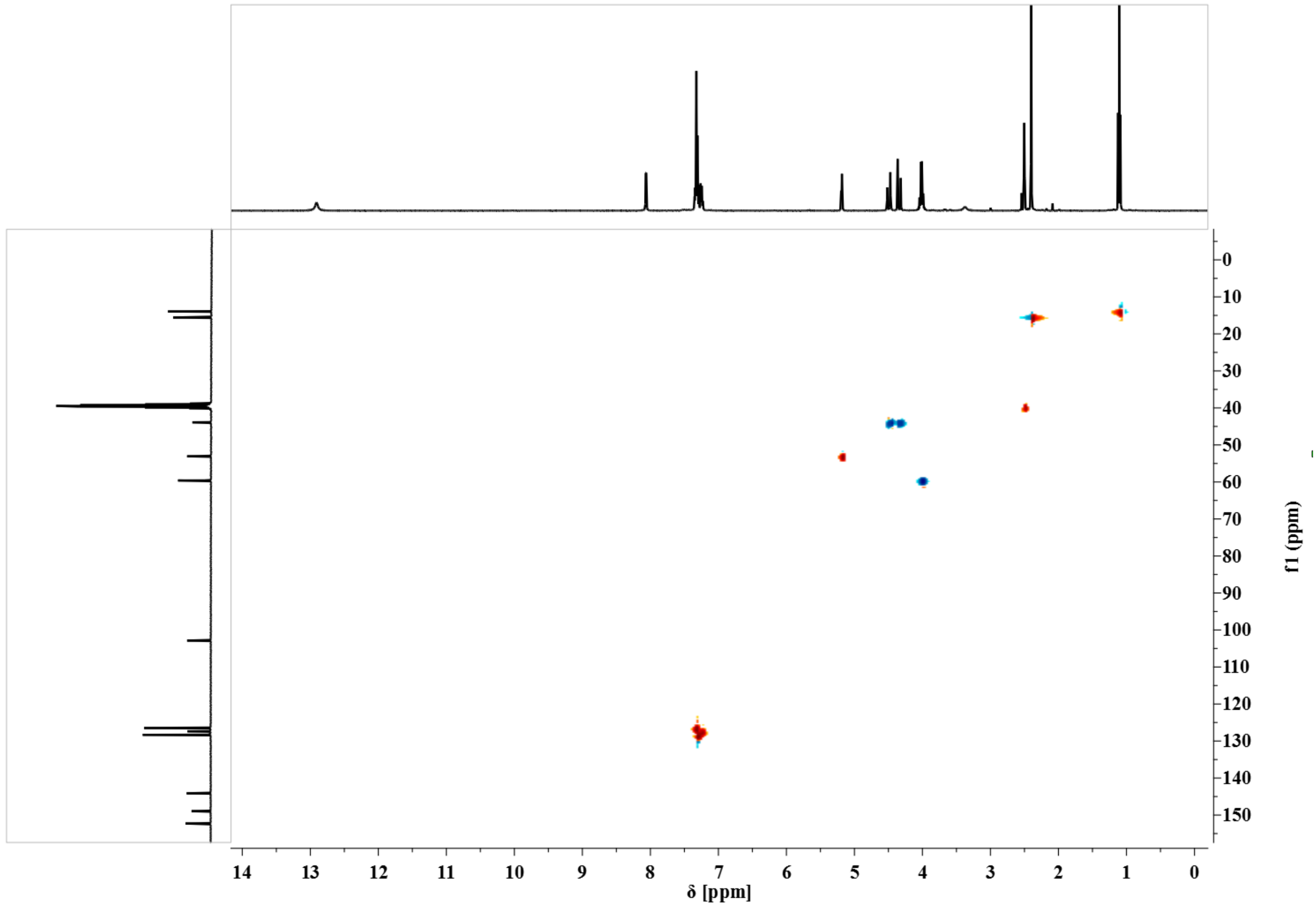
δ [ppm]
f1 (ppm)

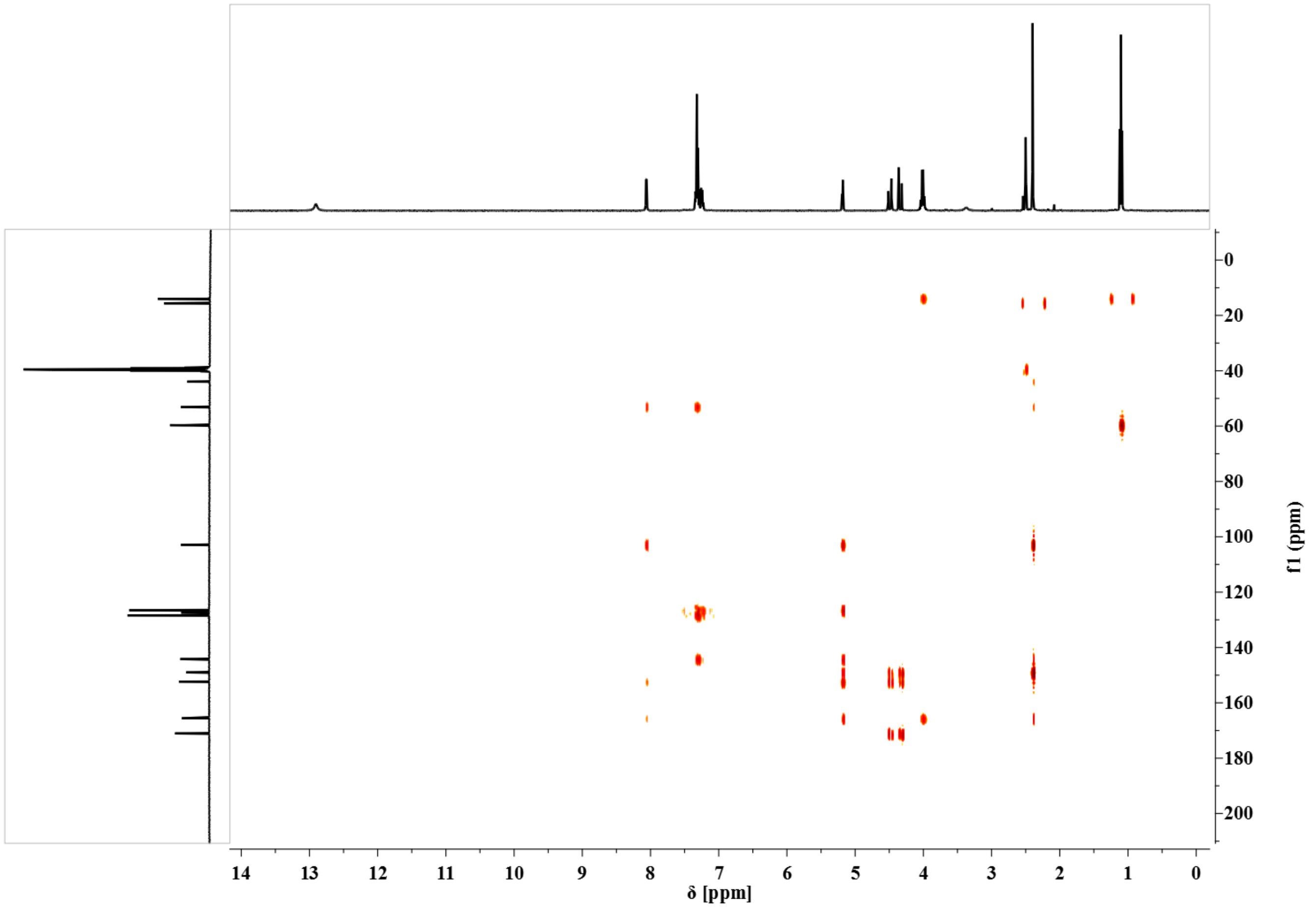
δ [ppm]
f1 (ppm)

Biginelli compound 16 derived from *N*-carbamoylglycine, benzaldehyde and benzyl acetoacetate

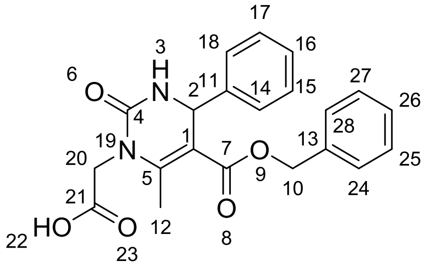

In a 25 mL round bottom flask, finely powdered *N*-carbamoylglycine (4.00 g, 33.8 mmol, 1.20 equiv) and benzaldehyde (2.99 g, 28.2 mmol, 2.88 mL, 1.00 equiv) were suspended in 6 mL dimethyl sulfoxide (4.7 M for 1.00 equiv). Subsequently, benzyl acetoacetate (4.40 g, 33.8 mmol, 4.32 mL) and 4-methylbenzenesulfonic acid (*p*-TSA) (169 mg, 1.42 mmol, 0.05 equiv) were added. The resulting mixture was stirred at 110 °C for 48 h. Subsequently, the crude reaction mixture was added dropwise into 300 mL water while stirring. The suspension was stirred for 4 h until a precipitate was formed. The precipitate was filtered off, crushed and washed with water (4 × 50 mL), dried and washed again with *n*-hexane/ethyl acetate (9.1) (3 × 100 mL) and dried under reduced pressure in a desiccator over calcium chloride to yield the Biginelli product **16** as a colorless solid (8.35 g, 21.9 mmol, 77.7%).

IR (ATR): ν [cm^{-1}] = 3258.6 (br, ν(CO_2H)), 3077.5 (br, ν(N-H)), 2938.8 (w, ν(C-H), 1709.2 (s, ν(C=O)), 1649.3 (s, ν(C=O)), 1430.7 (m), 1403.4 (m), 1386.8 (m), 1356.9 (w), 1315.2 (w), 1277.7 (m), 1239.1 (m), 1222.4 (m), 1205.4 (s), 1181.7 (vs), 1114.2 (s), 1055.9 (m), 977.8 (w), 830.5 (w), 782.8 (m), 733.0 (w), 694.9 (s), 641.8 (m), 592.4 (w), 502.6 (w), 461.1 (w).

^{1}H NMR (300 MHz, DMSO-d_6): δ [ppm] = 12.95 (br s, 1 H, OH^{22}), 8.08 (d, J = 3.5 Hz, 1 H, NH^{3}), 7.76 – 6.88 (m, 10 H, CH_{Ar}), 5.21 (d, J = 3.5 Hz, 1 H, CH^{2}), 5.06 (s, 2 H, CH_2^{10}), AB-signal (δ A = 4.50, δ B = 4.34, J_{AB} = 18.1 Hz, CH_2^{20}), 2.43 (s, 3 H, CH_3^{12}).

^{13}C NMR (100 MHz, DMSO-d_6): δ [ppm] = 171.00 (s, CO_2H^{21}), 165.31 (s, CO_2R^{7}), 152.23 (s, CO^{4}), 149.97 (s, C^{5}), 143.95 (s, C_{Ar}^{11}), 136.29 (s, C_{Ar}^{13}), 128.40 (s, CH_{Ar}), 128.32 (s, CH_{Ar}), 127.83 (s, CH_{Ar}), 127.70 (s, CH_{Ar}), 127.45 (s, CH_{Ar}), 126.57 (s, CH_{Ar}), 102.38 (s, C^{1}), 65.27 (s, CH_2^{10}), 53.04 (s, CH^{2}), 43.99 (s, CH_2^{20}), 15.70 (s, CH_3^{12}).

FAB – MS [*m/z*] (relative intensity): 381.2 (35%) $[M + H]^+$, 321.2 (10%) [Fragment B]$^+$, 289.1 (25%) [Fragment A]$^+$.

HRMS – FAB [*m/z*]: $[M + H]^+$ calculated for $^{12}C_{21}{}^{1}H_{21}{}^{16}O_5{}^{14}N_2$, 381.1445; found, 381.1146; Δ = 0.15 mmu.

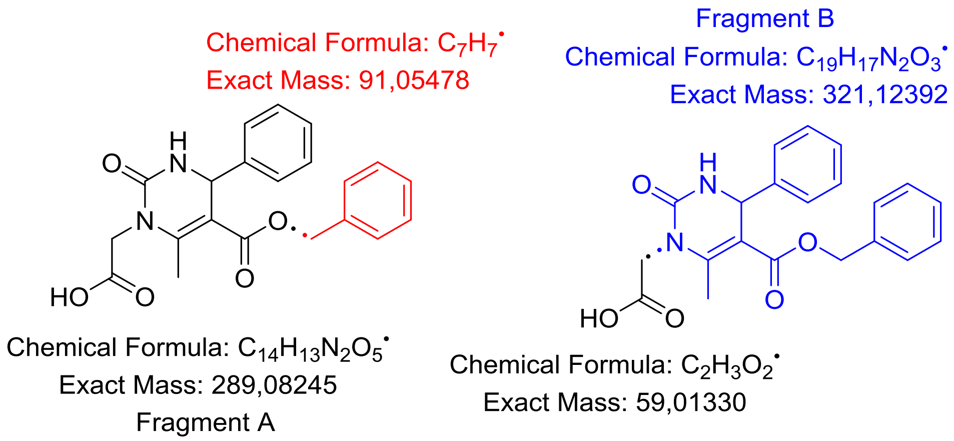
Chemical Formula: $C_7H_7^{\bullet}$
Exact Mass: 91,05478
Fragment B
Chemical Formula: $C_{19}H_{17}N_2O_3^{\bullet}$
Exact Mass: 321,12392
Chemical Formula: $C_{14}H_{13}N_2O_5^{\bullet}$
Exact Mass: 289,08245
Fragment A
Chemical Formula: $C_2H_3O_2^{\bullet}$
Exact Mass: 59,01330

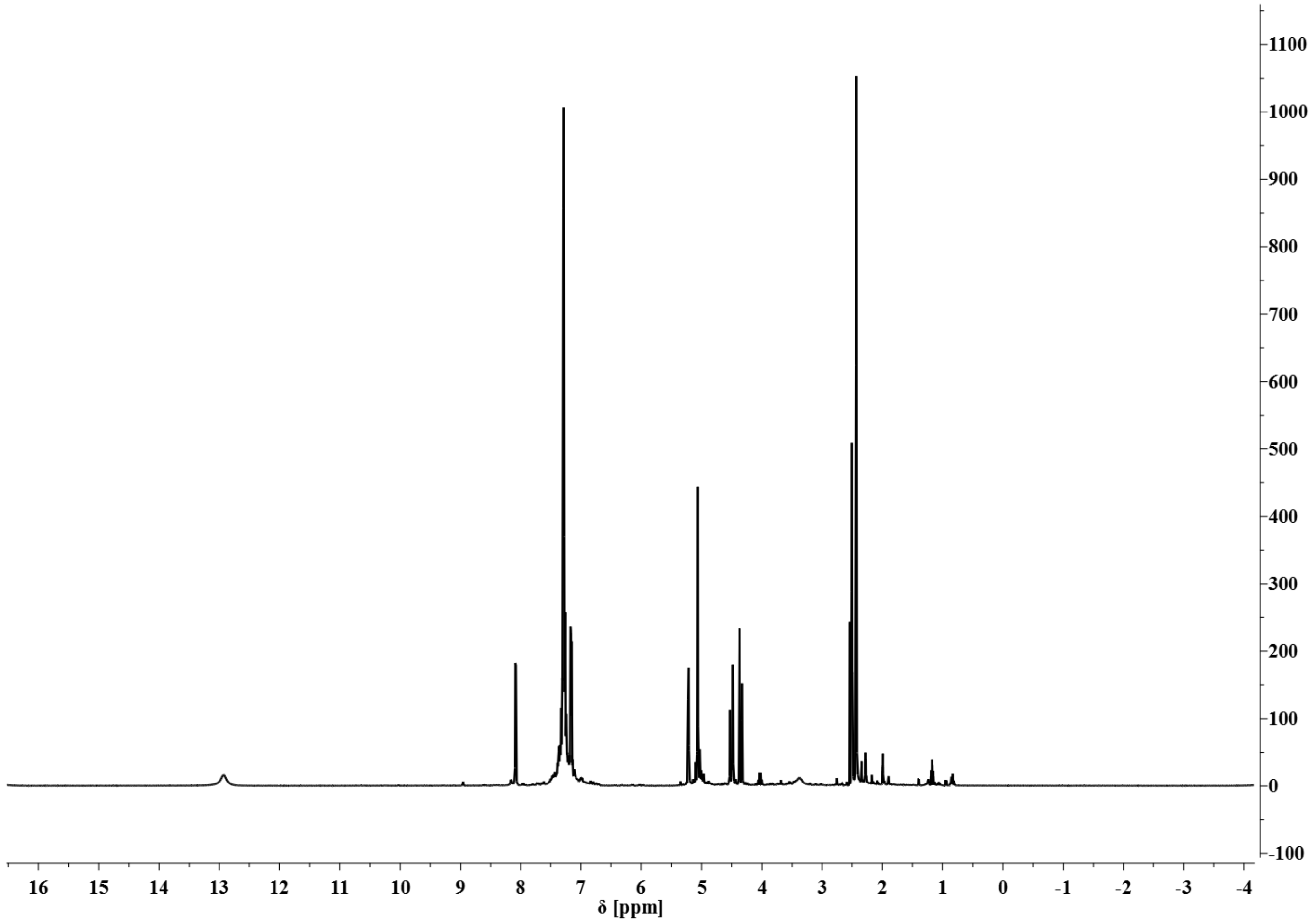
δ [ppm]

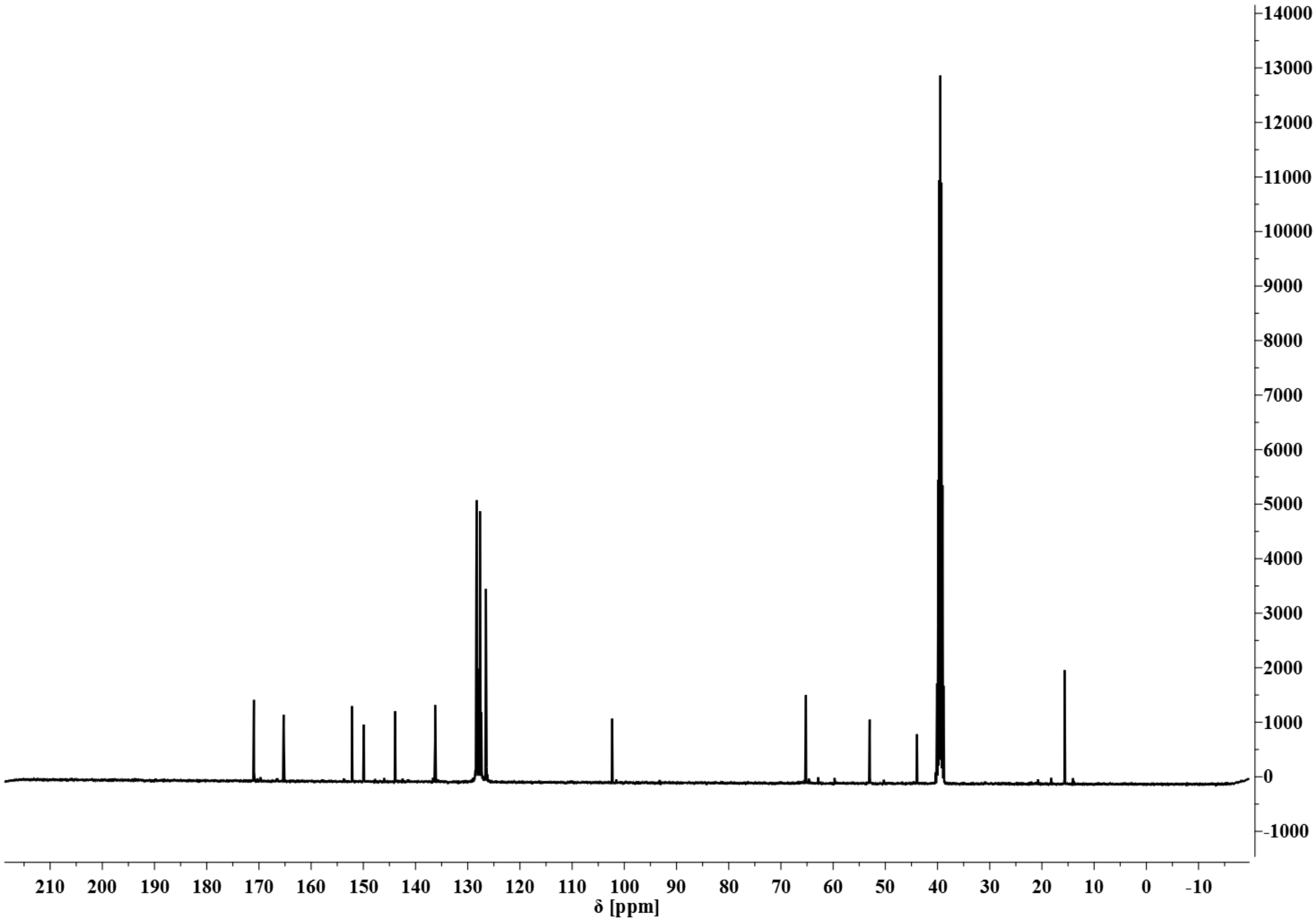

14000
13000
12000
11000
10000
9000
8000
7000
6000
5000
4000
3000
2000
1000
0
-1000
210 200 190 180 170 160 150 140 130 120 110 100 90 80 70 60 50 40 30 20 10 0 -10
δ [ppm]

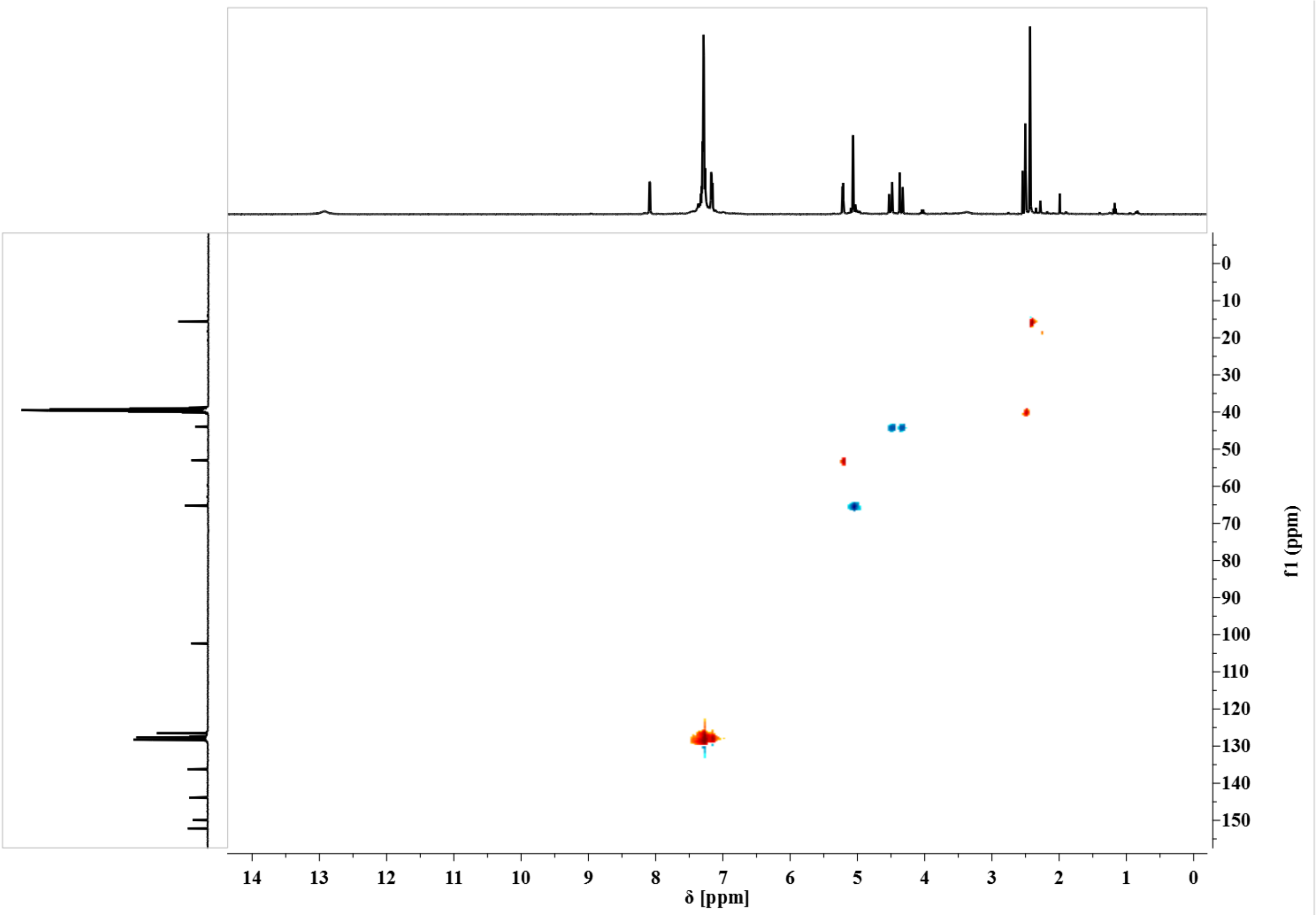

0 10 20 30 40 50 60 70 80 90 100 110 120 130 140 150
f1 (ppm)
14 13 12 11 10 9 8 7 6 5 4 3 2 1 0
δ [ppm]

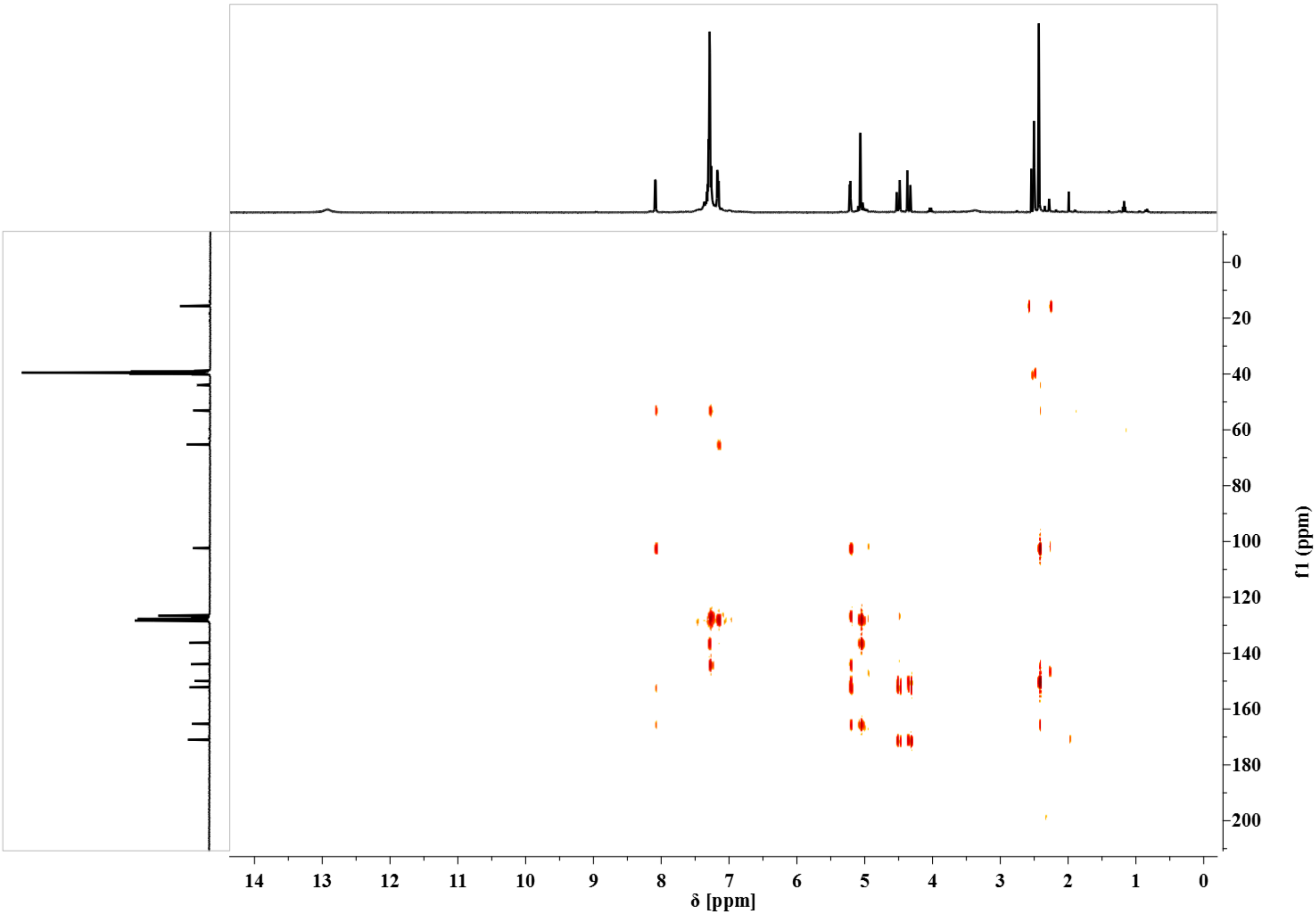

f1 (ppm)
0
20
40
60
80
100
120
140
160
180
200
14
13
12
11
10
9
8
7
6
5
4
3
2
1
0
δ [ppm]

Biginelli acid 17 derived from 4-formylbenzoic acid, urea and ethyl acetoacetate

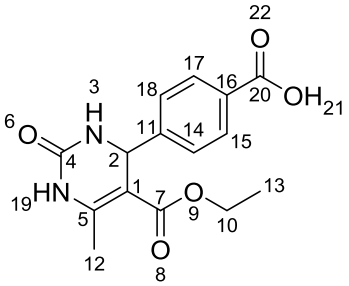

In a 25 mL round-bottomed flask, finely powdered urea (1.50 g, 24.9 mmol, 1.50 equiv) and 4-formylbenzoic acid (2.50 g, 16.6 mmol, 1.00 equiv) were suspended in 4 mL DMSO. Subsequently, ethyl acetoacetate (3.25 g, 24.9 mmol, 1.50 equiv) and 4-methylbenzenesulfonic acid (*p*-TSA) (200 mg, 1.65 mmol, 0.10 equiv) were added. The resulting mixture was stirred at 110 °C for 48 h and subsequently at 40 °C for 24 h. Subsequently, the crude reaction mixture was diluted with ethanol and added dropwise into 300 mL water. The suspension was stirred for 1 h until a precipitate was formed. The precipitate was filtered off, crushed, washed with water (3 × 20 mL) and dried. Afterwards, the precipitate was washed with and *n*-hexane (3 × 20 mL). After drying under reduced pressure in a desiccator over calcium chloride, the Biginelli acid **17** was obtained as a colorless solid (4.57 g, 15.1 mmol, 90.1%).

IR (ATR): ν [cm^{-1}] = 3309.4 (br, ν(O-H)), 3206.7 (br, ν(N-H)), 3084.8 (br, ν(N-H)), 2971.9 (br, ν(C-H), 1723.3 (s, ν(C=O)), 1702.5 (s, ν(C=O)), 1649.1 (s, ν(C=O)), 1608.9 (m), 1474.3 (m), 1419.7 (w), 1379.8 (w), 1317.7 (w), 1288.1 (w), 1225.0 (vs), 1170.0 (m), 1087.6 (s), 1018.3 (m), 875.7 (w), 843.2 (w), 792.4 (m), 754.5 (s), 700.5 (m), 667.9 (w), 638.1 (s), 568.9 (w), 527.2 (w), 501.4 (w), 466.9 (w), 408.5 (w).

^{1}H NMR (300 MHz, DMSO-d_6): δ [ppm] = 12.90 (s, 1 H, CO_2H^{21}), 9.27 (s, 1 H, NH^{19}), 7.91 (d, J = 7.8 Hz, 2 H, $CH_{Ar}^{15,17}$), 7.81 (s, 1 H, NH^{3}), 7.35 (d, J = 7.9 Hz, 2 H, $CH_{Ar}^{14,18}$), 5.21 (d, J = 2.8 Hz, 1 H, CH^{2}), 3.97 (q, J = 6.9 Hz, 2 H, CH_2^{10}), 2.25 (s, 3 H, CH_3^{12}), 1.08 (t, J = 7.1 Hz, 3 H, CH_3^{13}).

^{13}C NMR (75 MHz, DMSO-d_6): δ [ppm] = 167.13 (s, CO_2H^{20}), 165.24 (s, CO_2R^{7}), 152.00 (s, CO^{4}), 149.57 (s, C^{5}), 148.91 (s, C_{Ar}^{11}), 129.84 (s, C_{Ar}^{16}), 129.66 (s, $CH_{Ar}^{15,\ 17}$), 126.56 (s, $CH_{Ar}^{14,18}$), 98.71 (s, C^{1}), 59.32 (s, CH_2^{10}), 53.93 (s, CH^{2}), 17.88 (s, CH_3^{12}), 14.10 (s, CH_3^{13}).

FAB – MS [*m/z*] (relative intensity): 305.1 (100%) $[M + H]^+$.

HRMS – FAB [*m/z*]: $[M + H]^+$ calculated for $^{12}C_{15}{}^{1}H_{17}{}^{16}O_5{}^{14}N_2$, 305.1132; found, 305.1131; Δ = 0.12 mmu.

NMR Spectral data is in accordance to those reported by Fernandes *et al.*[5]

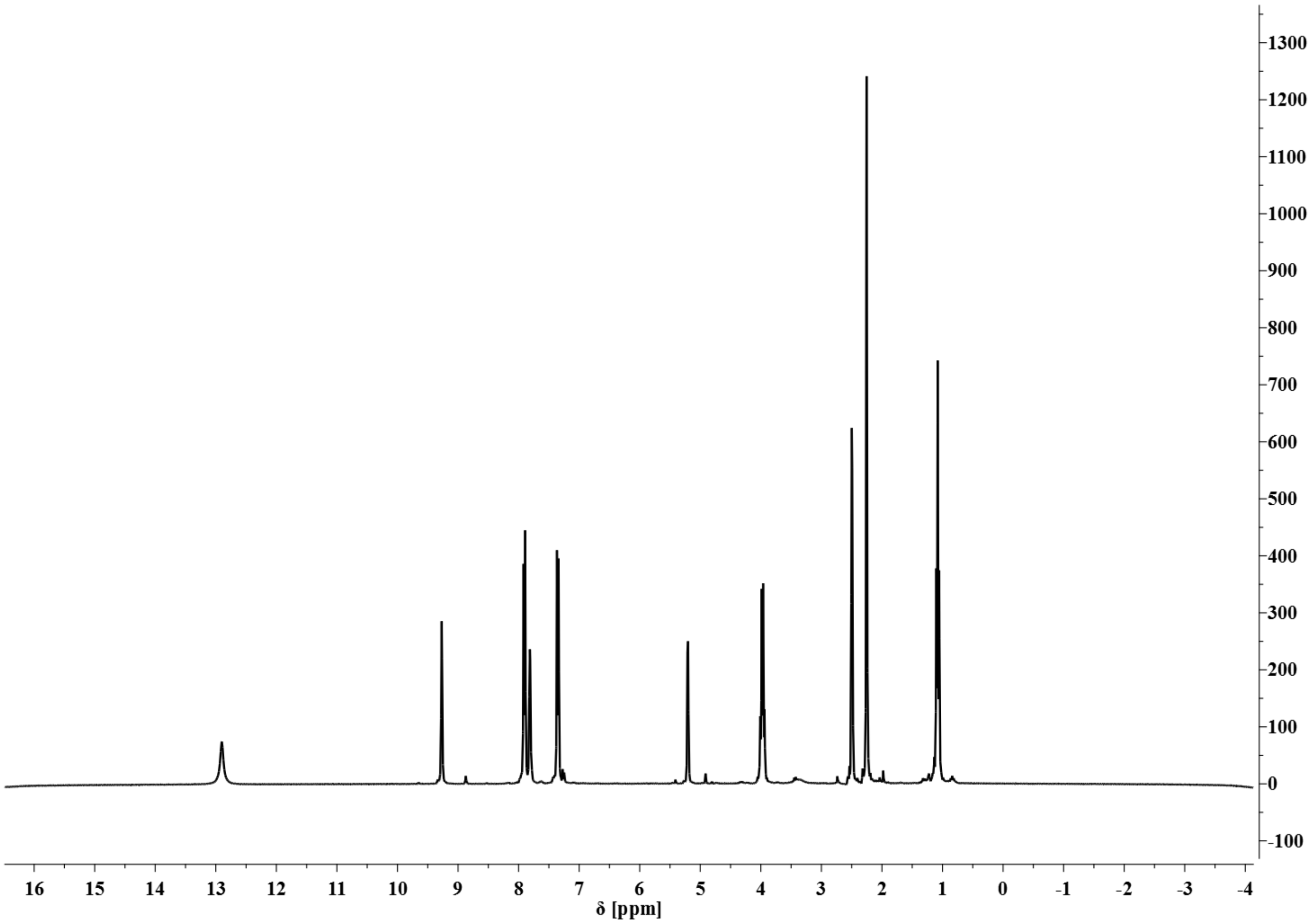
δ [ppm]

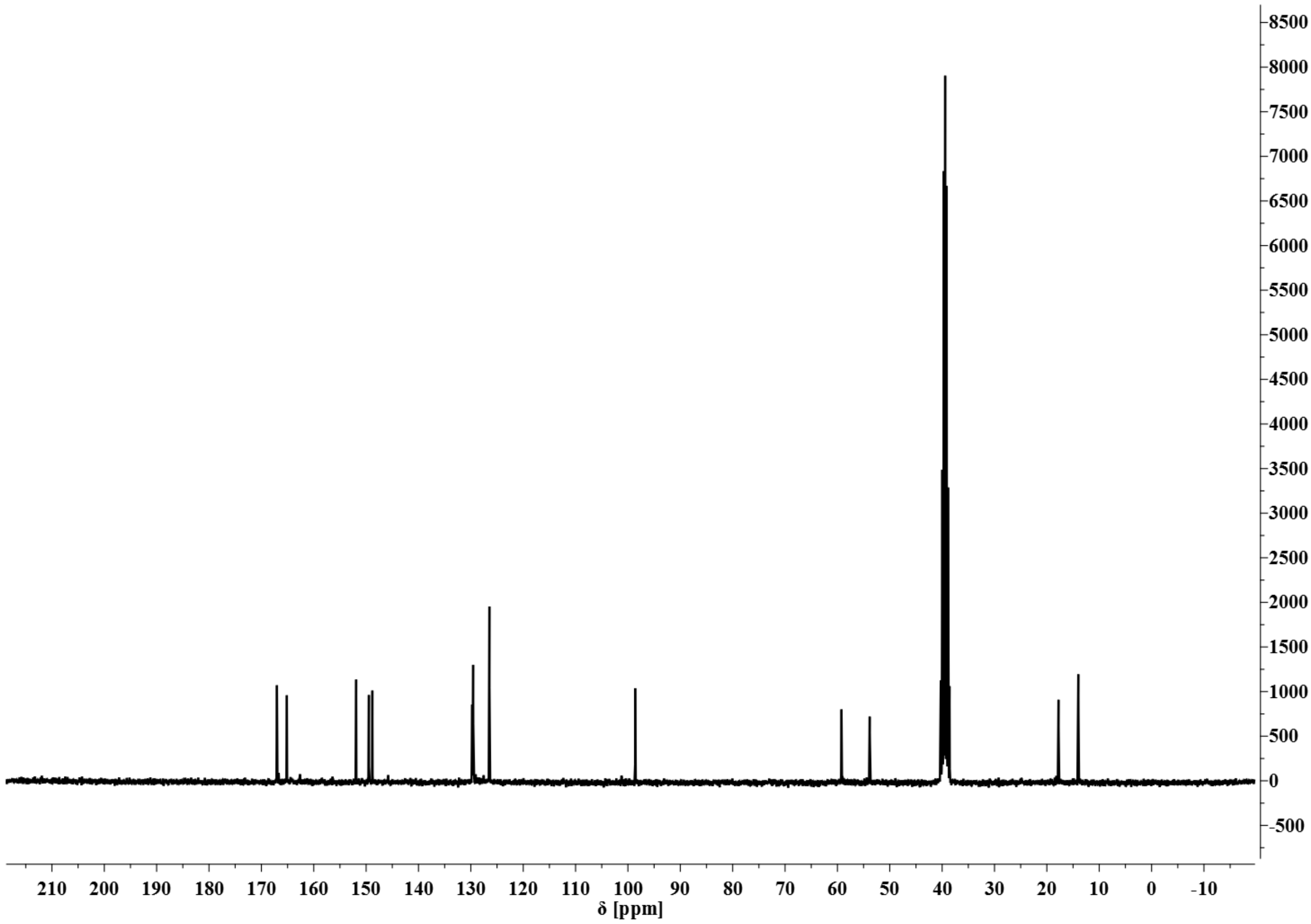
δ [ppm]

Biginelli acid 18 derived from 4-formylbenzoic acid, urea and benzyl acetoacetate

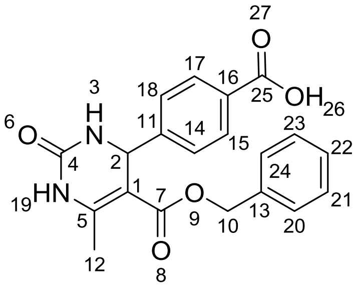

In a 25 mL round-bottomed flask, finely powdered urea (1.50 g, 24.9 mmol, 1.50 equiv) and 4-formylbenzoic acid (2.50 g, 16.6 mmol, 1.00 equiv) were suspended in 4 mL dimethyl sulfoxide. Subsequently, benzyl acetoacetate (3.84 g, 20.0 mmol, 1.20 equiv) and 4-methylbenzenesulfonic acid (*p*-TSA) (200 mg, 1.65 mmol, 0.10 equiv) were added. The resulting mixture was stirred at 110 °C for 48 h and subsequently at 40 °C for 24 h. Subsequently, the crude reaction mixture was diluted with ethanol and added dropwise into 300 mL water. The suspension was stirred for 1 h until a precipitate was formed. The precipitate was skimmed, collected, washed with water (3 × 20 mL) and dried. Afterwards the precipitate was washed with *n*-hexane (3 × 20 mL). After drying under reduced pressure in a desiccator over calcium chloride acid **18** was obtained as a colorless solid (5.55 g, 15.2 mmol, 91.0%).

IR (ATR): ν [cm^{-1}] = 3273.7 (br, ν(N-H)), 3068.9 (br, ν(N-H)), 2931.0 (w, ν(C-H)), 1678.3 (s, ν(C=O)), 1642.6 (s, ν(C=O)), 1608.6 (m), 1466.5 (m), 1381.5 (m), 1325.2 (m), 1275.3 (m), 1228.5 (s), 1090.8 (s), 1017.7 (w), 953.9 (w), 909.7 (w), 860.3 (w), 773.3 (m), 738.2 (m), 694.8 (m), 665.2 (m), 642.2 (w), 577.3 (w), 518.3 (m), 504.9 (m), 452.8 (w), 394.7 (w).

^{1}H NMR (400 MHz, DMSO-d_6): δ [ppm] = 12.92 (s, 1 H, CO_2H^{26}), 9.35 (d, J = 1.4 Hz, 1 H, NH^{19}), 7.88 (d, J = 8.3 Hz, 3 H, CH_{Ar}), 7.85 – 7.83 (m, 1 H, NH^{3}), 7.32 (d, J = 8.3 Hz, 2 H, $CH_{Ar}{}^{14,18}$), 7.30 – 7.25 (m, 3 H, CH_{Ar}), 7.19 – 7.08 (m, 2 H, CH_{Ar}), 5.24 (d, J = 3.2 Hz, 1 H, CH^{2}), 5.15 – 4.90 (m, 2 H, $CH_2{}^{10}$), 2.28 (s, 3 H, $CH_3{}^{12}$).

^{13}C NMR (101 MHz, DMSO-d_6): δ [ppm] = 167.10 (s, CO_2H^{25}), 164.96 (s, CO_2R^{7}), 151.83 (s, CO^{4}), 149.76 (s, C^{5}), 149.33 (s, $C_{Ar}{}^{16}$), 136.46 (s, $C_{Ar}{}^{11}$), 129.86 (s, $C_{Ar}{}^{13}$), 129.66 (s, CH_{Ar}), 128.28 (s, CH_{Ar}), 127.79 (s, CH_{Ar}), 127.66 (s, CH_{Ar}), 126.57 (s, CH_{Ar}), 98.20 (s, C^{1}), 64.91 (s, $CH_2{}^{10}$), 53.87 (s, CH^{2}), 17.95 (s, $CH_3{}^{12}$).

FAB – MS [*m/z*] (relative intensity): 367.1 (100%) $[M + H]^+$.

HRMS – FAB [*m/z*]: $[M + H]^+$ calculated for $^{12}C_{20}{}^{1}H_{19}{}^{16}O_5{}^{14}N_2$, 367.1288; found, 367.1289; Δ = 0.05 mmu.

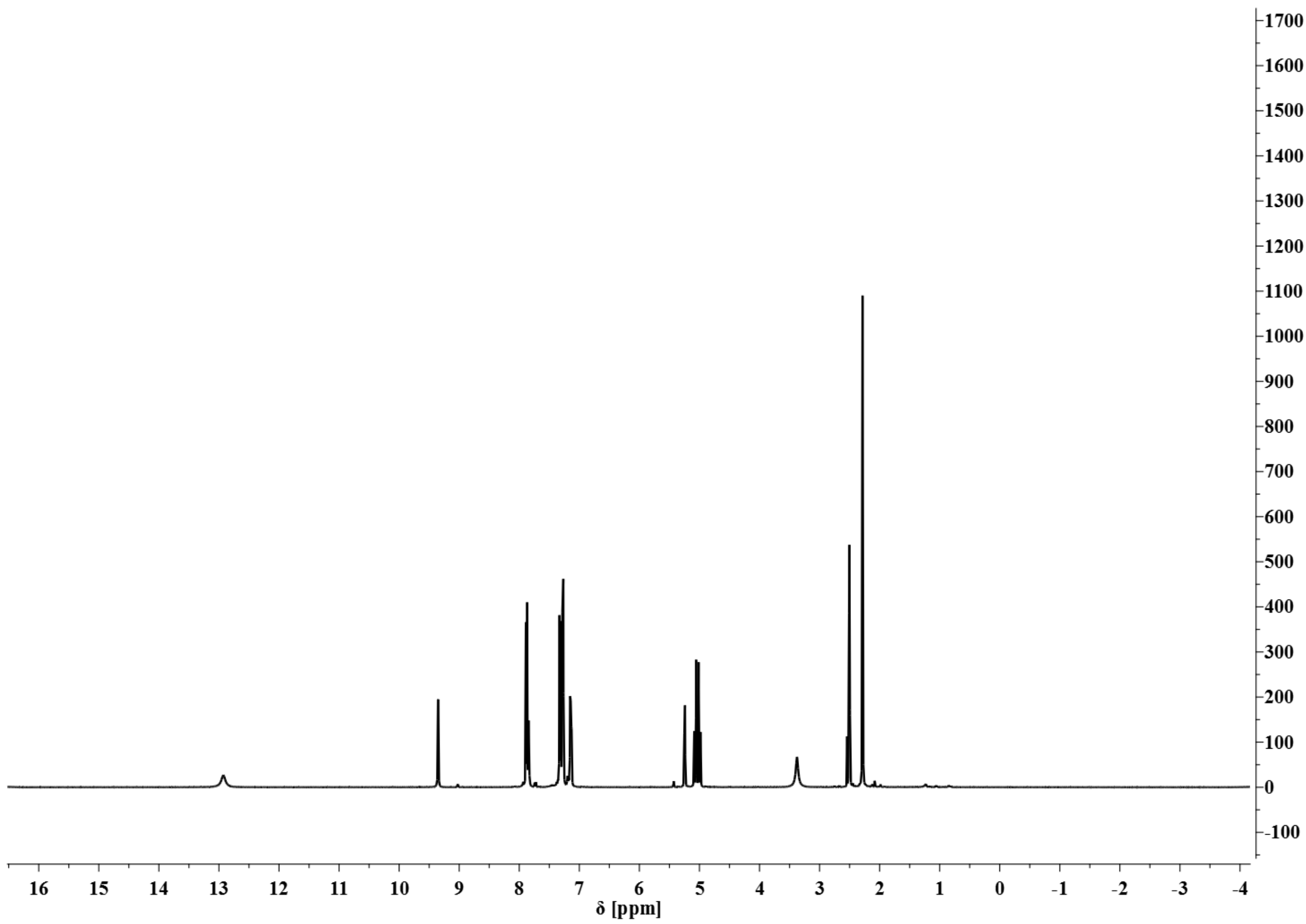

1700
1600
1500
1400
1300
1200
1100
1000
900
800
700
600
500
400
300
200
100
0
-100
16 15 14 13 12 11 10 9 8 7 6 5 4 3 2 1 0 -1 -2 -3 -4
δ [ppm]

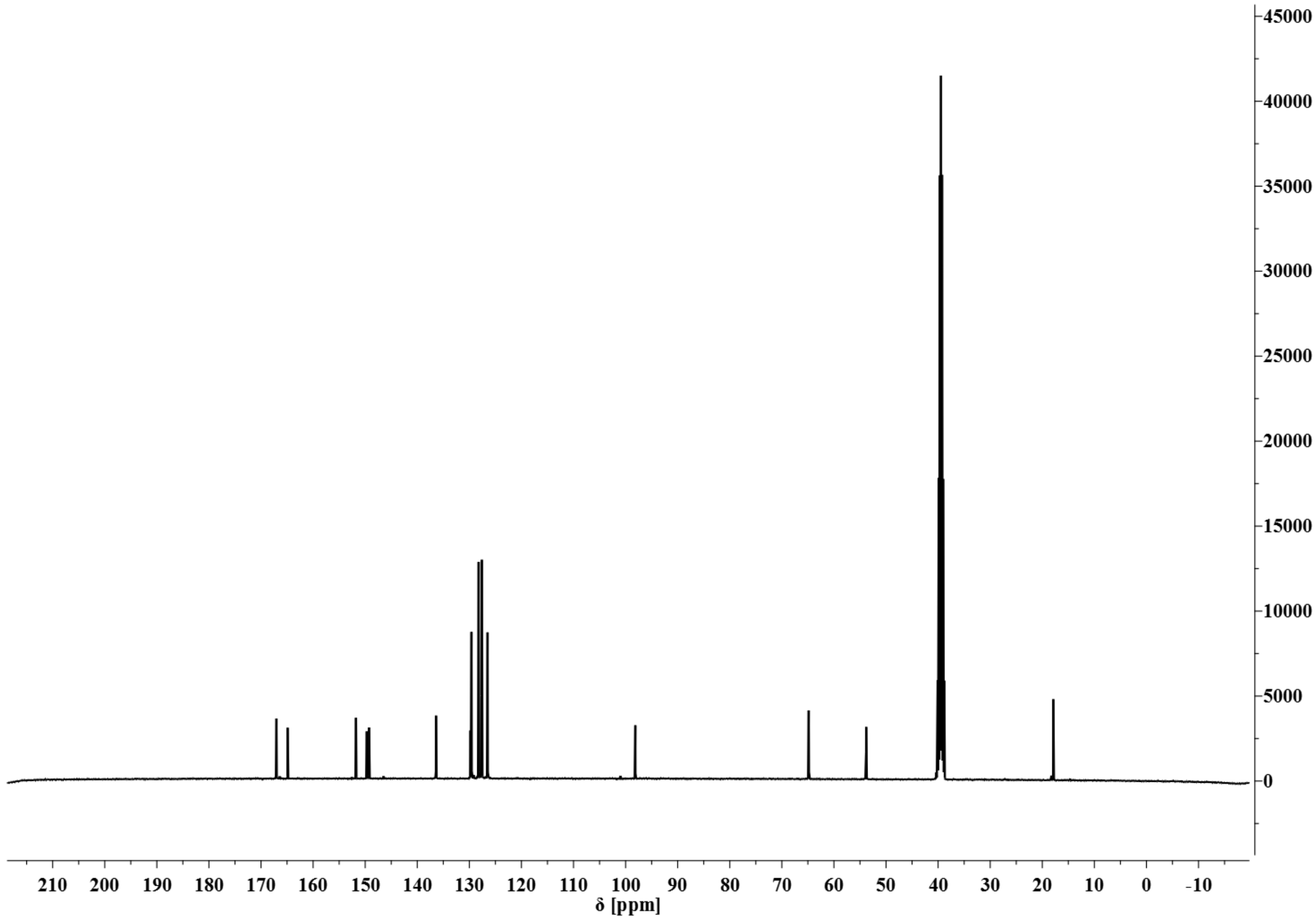

45000
40000
35000
30000
25000
20000
15000
10000
5000
0
210 200 190 180 170 160 150 140 130 120 110 100 90 80 70 60 50 40 30 20 10 0 -10
δ [ppm]

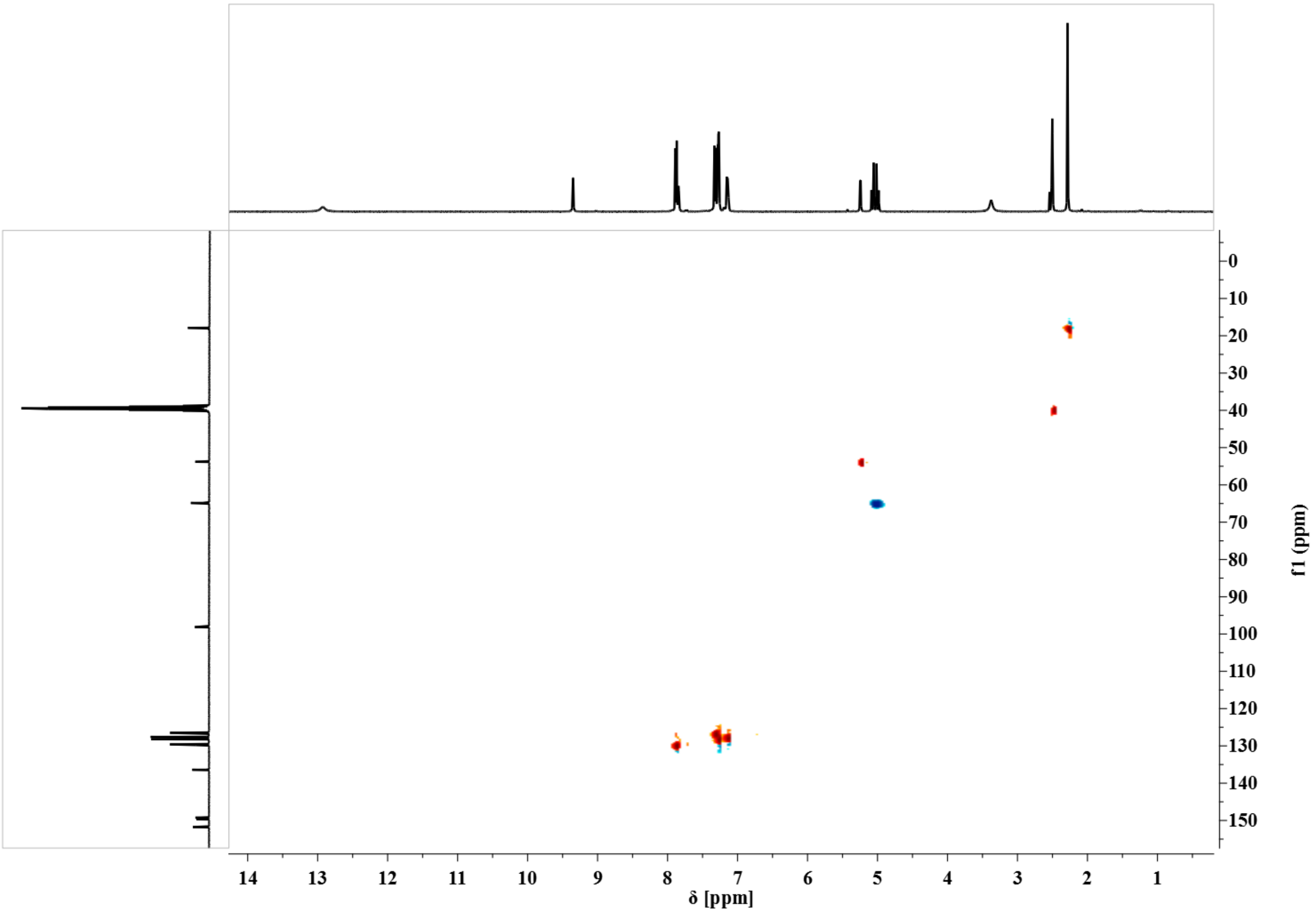

0
10
20
30
40
50
60
70
80
90
100
110
120
130
140
150
f1 (ppm)
14
13
12
11
10
9
8
7
6
5
4
3
2
1
δ [ppm]

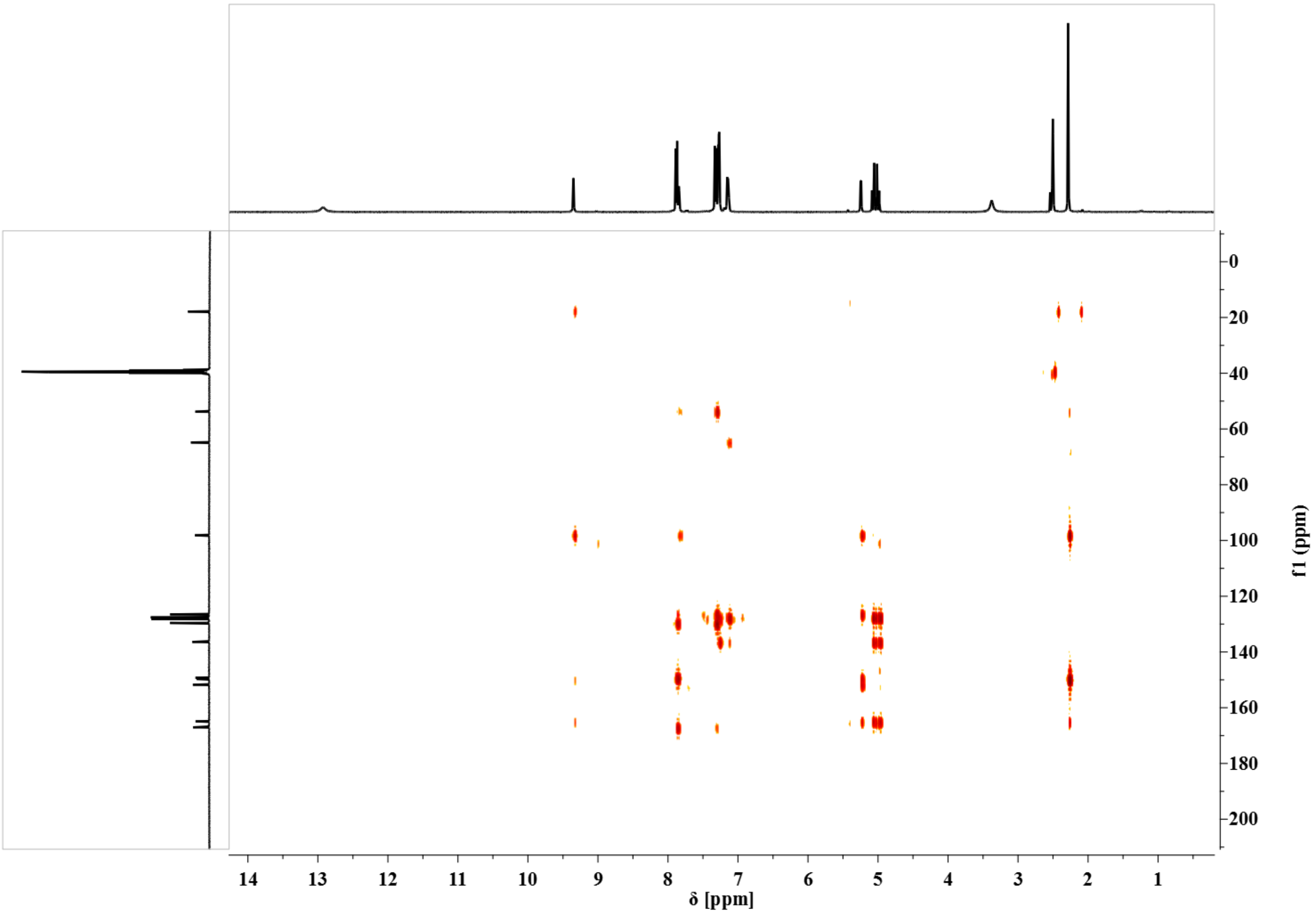

0
20
40
60
80
100
120
140
160
180
200
f1 (ppm)
14
13
12
11
10
9
8
7
6
5
4
3
2
1
δ [ppm]

Passerini product 19 derived from Biginelli acid 17, *tert*-butyl isocyanide and heptanal

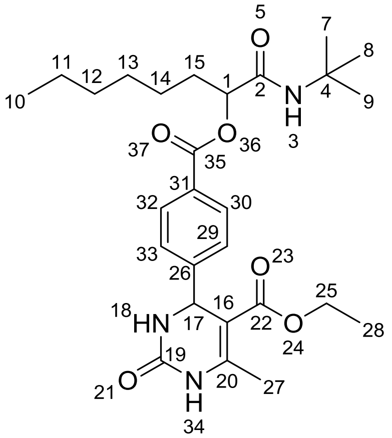

In a tube vial, finely powdered Biginelli acid **17** (300 mg, 986 µmol, 1.00 equiv) was dissolved in 0.5 mL dimethyl sulfoxide and diluted with 0.9 mL dichloromethane. Subsequently, heptanal (169 mg, 1.48 mmol, 205 µL, 1.50 equiv) and *tert*-butylisocyanide (123 mg, 1.48 mmol, 142 µL, 1.50 equiv) were added. The mixture was stirred at room temperature for 2 d. Subsequently, the crude reaction mixture was added dropwise into a stirred mixture of 10 mL water and 10 mL dichloromethane. The aqueous phase was extracted with 10 mL dichloromethane three times. The combined organic phases were concentrated under reduced pressure. The concentrate was added dropwise into 30 mL of *n*-hexane/ethyl acetate (4:1) while stirring. After stirring for 1 h, a precipitate was formed, which was separated, washed with 30 mL *n*-hexane three times and dried under reduced pressure. The Passerini product **19** was obtained as a colorless solid (332 mg, 661 µmol, 67.1%).

IR (ATR): ν [cm^{-1}] = 3292.8 (br, ν(N-H)), 3086.2 (br, ν(N-H)), 2926.6 (m, ν(C-H), 1704.6 (vs, ν(C=O)), 1645.3 (s, ν(C=O)), 1607.6 (w), 1554.3 (w), 1453.5 (m), 1426.2 (m), 1407.4 (w), 1364.5 (w), 1312.9 (m), 1262.7 (m), 1219.1 (vs), 1179.6 (w), 1099.6 (s), 1017.2 (w), 949.2 (w), 868.2 (w), 825.6 (w), 763.5 (m), 706.7 (m), 654.5 (m), 605.5 (w), 522.6 (w), 456.2 (w).

^{1}H NMR (300 MHz, DMSO-d_6): δ [ppm] = 9.29 (s, 1 H, NH^{34}), 7.94 (d, J = 7.8 Hz, 2 H, $CH_{Ar}^{32,30}$), 7.84 (s, 1 H, NH^{18}), 7.63 (s, 1 H, NH^{3}), 7.39 (d, J = 7.8 Hz, 2 H, $CH_{Ar}^{29,33}$), 5.22 (s, 1 H, CH^{17}), 4.98 (s, 1 H, CH^{1}), 3.98 (q, J = 6.9 Hz, 2 H, CH^{25}), 2.26 (s, 3 H, CH_3^{27}), 1.86 – 1.60 (m, 2 H, CH_2^{15}), 1.43 – 1.16 (m, 19 H, CH_2+CH_3^{7-9}), 1.09 (t, J = 6.9 Hz, 4 H, CH_3^{28}), 0.93 – 0.74 (m, 3 H, CH_3^{10}).

^{13}C NMR (75 MHz, DMSO-d_6): δ [ppm] = 168.51 (s, $CONR^{2}$), 165.16 (s, CO_2R^{22}), 164.92 (s, CO_2R^{35}), 151.92 (s, CO^{19}), 150.10 (s, C^{20}), 148.97 (s, C_{Ar}^{26}), 129.62 (s, $CH_{Ar}^{30,32}$), 128.67 (s, C_{Ar}^{31}), 126.66 (s, $CH_{Ar}^{29,33}$), 98.57 (s, C^{16}), 74.04 (s, CH^{1}), 59.29 (s, CH_2^{25}), 53.90 (s, CH^{17}), 50.22 (s, C^{4}), 31.65 (s, CH_2), 31.12 (s, CH_2), 28.45 (s, CH_3^{7-9}), 28.36 (s, CH_2), 24.63 (s, CH_2), 22.00 (s, CH_2), 17.82 (s, CH_3^{27}), 14.08 (s, CH_3^{28}), 13.91 (s, CH_3^{10}).

FAB – MS [*m/z*] (relative intensity): 502.3 (30%) $[M + H]^+$, 456.3 (10%) $[M - C_2H_5O]^+$, 287.1 (100%) $[\text{Fragment A}]^+$.

HRMS – FAB [*m/z*]: $[M + H]^+$ calculated for $^{12}C_{27}{}^{1}H_{40}{}^{16}O_6{}^{14}N_3$, 502.2912; found, 502.2911; Δ = 0.08 mmu.

Chemical Formula: $C_{12}H_{24}NO_2^{\bullet}$
Exact Mass: 214,18070

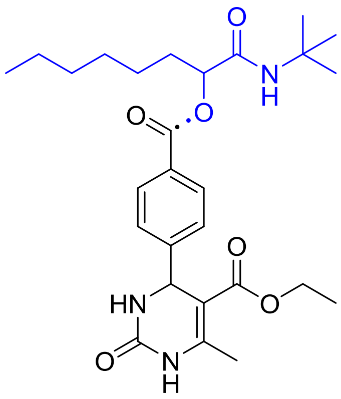

Chemical Formula: $C_{15}H_{15}N_2O_4^{\bullet}$
Exact Mass: 287,10318
Fragment A

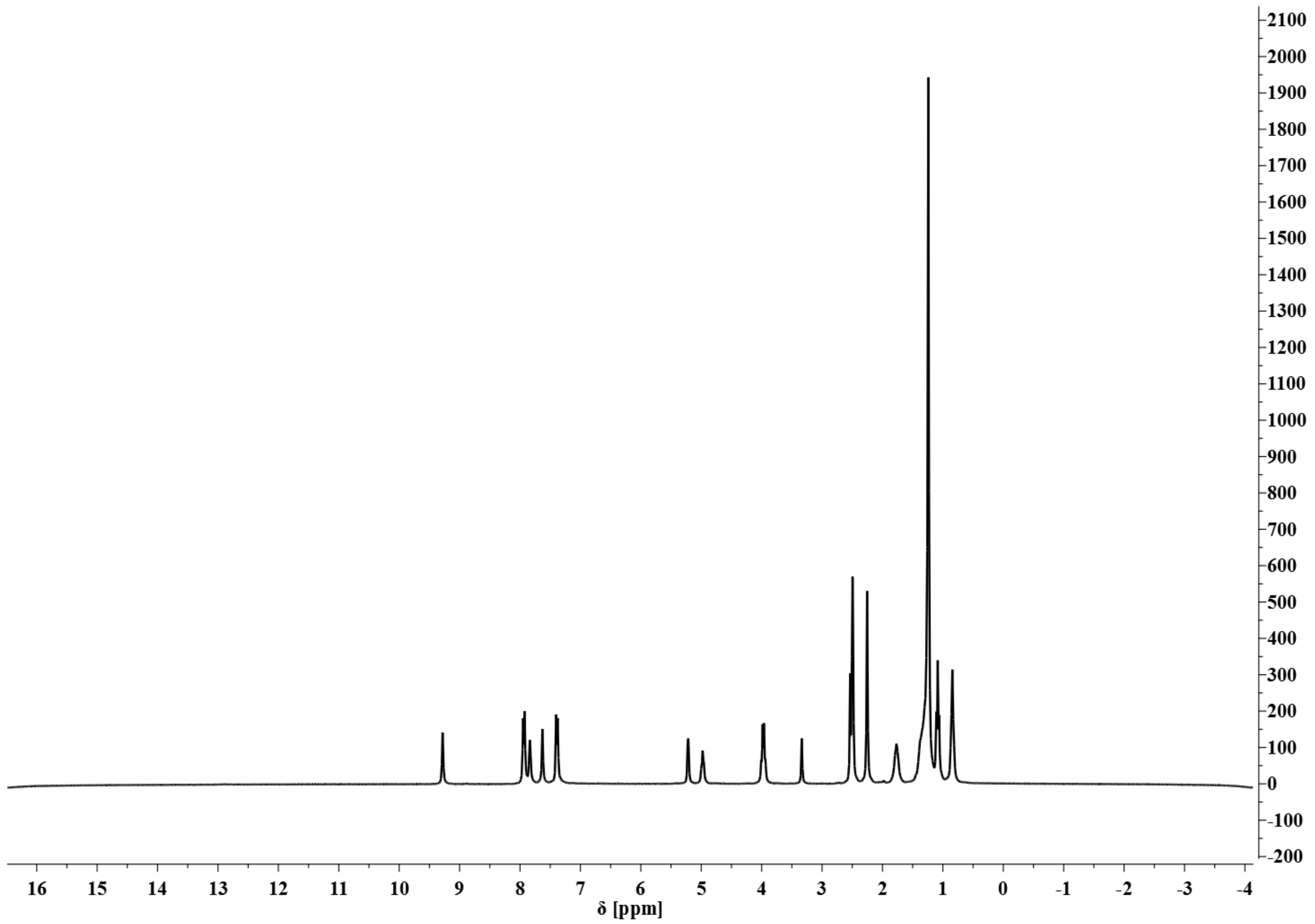
δ [ppm]

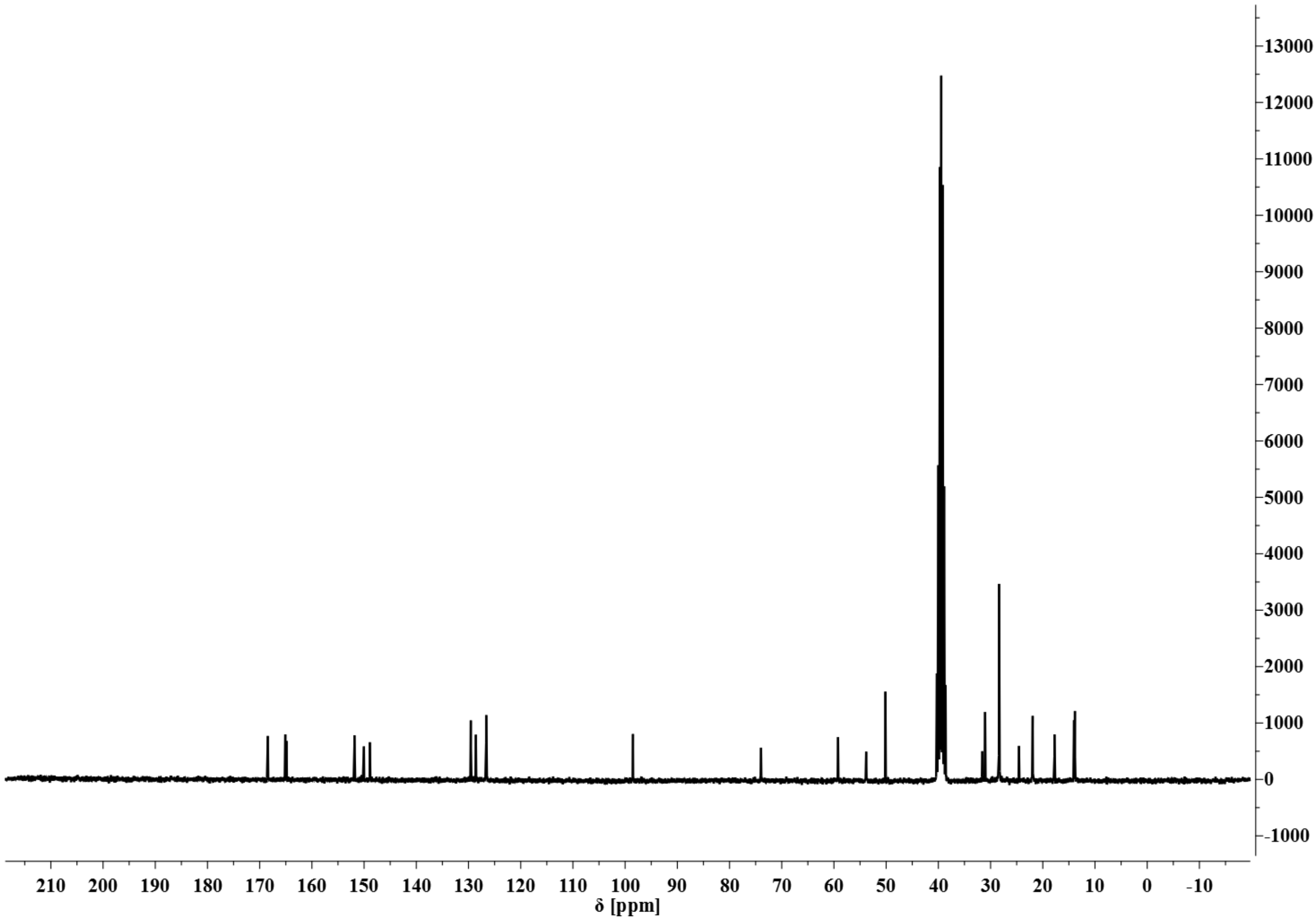
δ [ppm]

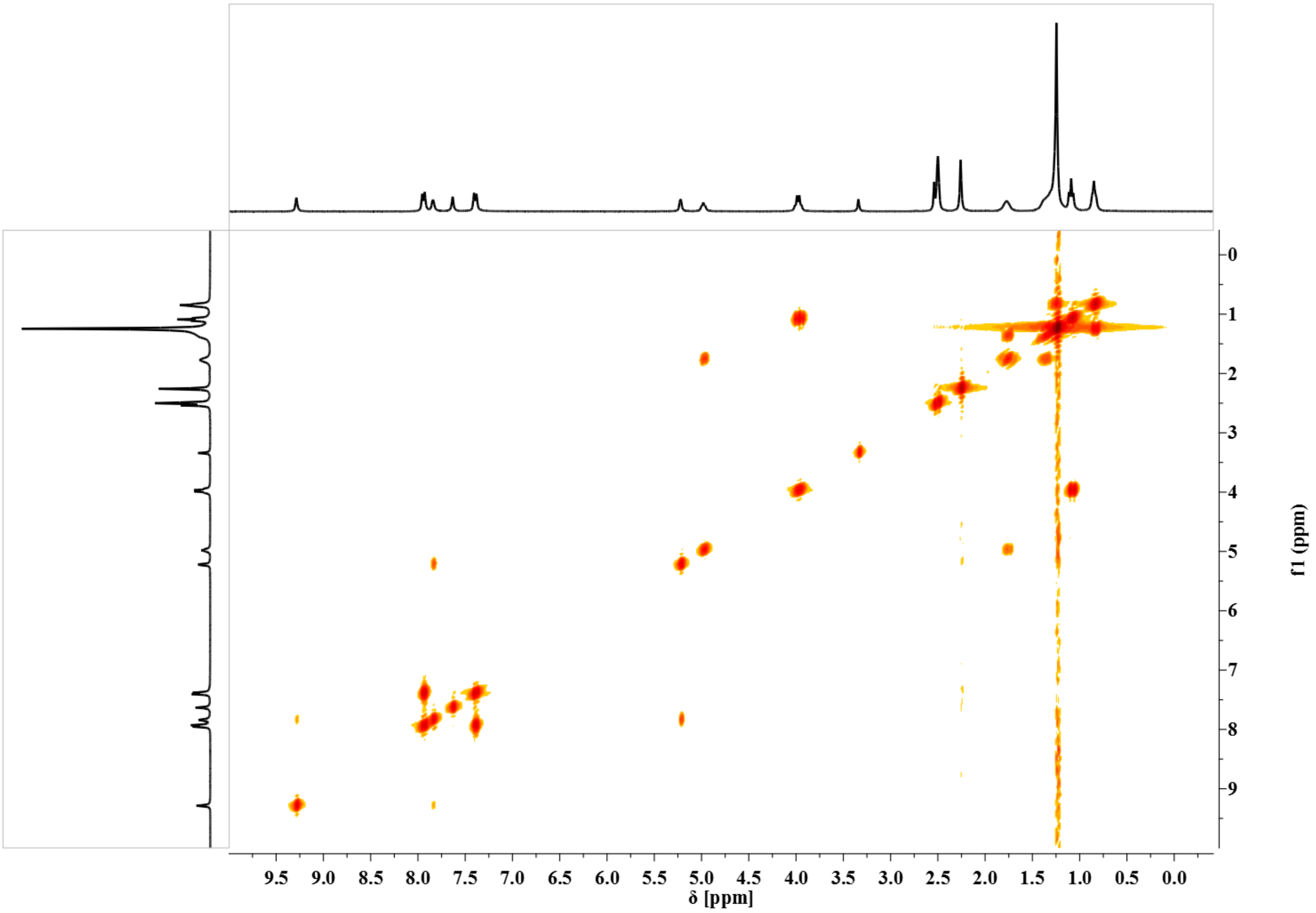

Passerini product 20 derived from Biginelli acid 18, *tert*-butyl isocyanide and heptanal

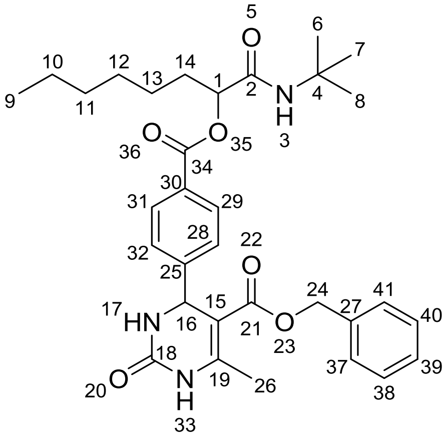

In a tube vial, finely powdered Biginelli acid **18** (300 mg, 819 µmol, 1.00 equiv) was dissolved in 1.5 mL dimethyl sulfoxide and diluted with 1.5 mL dichloromethane. Subsequently, heptanal (140 mg, 1.23 mmol, 171 µL, 1.50 equiv) and *tert*-butylisocyanide (102 mg, 1.23 mmol, 118 µL, 1.50 equiv) were added. The mixture was stirred at room temperature for 4 d. Subsequently, the crude reaction mixture was added dropwise into a stirred mixture of 10 mL water and 10 mL ethyl acetate. The aqueous phase was extracted with 10 mL ethyl acetate three times. The combined organic phases were concentrated under reduced pressure. The concentrate was purified via column chromatography employing silica gel and eluting with a gradual solvent

mixture of ethyl acetate/*c*-hexane (1:3 → 1:0). The Passerini product **20** was obtained as a colorless solid (103 mg, 182 µmol, 22.3%).

IR (ATR): ν [cm^{-1}] = 3233.8 (br, ν(N-H)), 3089.1 (br, ν(N-H)), 2926.9 (m, ν(C-H), 2858.2 (w, ν(C-H), 1697.2 (s, ν(C=O)), 1639.5 (s, ν(C=O)), 1523.2 (w), 1453.7 (m), 1380.0 (w), 1314.7 (w), 1261.5 (s), 1219.1 (vs), 1074.3 (s), 1017.6 (w), 859.8 (w), 825.0 (w), 794.6 (w), 753.0 (w), 696.2 (m), 651.6 (w), 586.2 (w), 524.7 (w), 491.7 (w), 438.9 (w).

^{1}H NMR (300 MHz, DMSO-d_6): δ [ppm] = 9.35 (s, 1 H, NH^{33}), 7.90 (d, J = 7.4 Hz, 2 H, $CH_{Ar}^{29,31}$), 7.85 (s, 1 H, NH^{17}), 7.64 (s, 1 H, NH^{3}), 7.34 (d, J = 8.0 Hz, 2 H, $CH_{Ar}^{28,32}$), 7.26 (d, J = 4.4 Hz, 3 H, CH_{Ar}), 7.17 – 7.08 (m, 2 H, CH_{Ar}), 5.25 (d, J = 2.8 Hz, 1 H, CH^{16}), 5.07 – 4.94 (m, 3 H, CH^{1} + CH_2^{24}), 2.28 (s, 3 H, CH_3^{26}), 1.91 – 1.66 (m, 2 H, CH_2^{14}), 1.45 – 1.13 (m, 19 H, $CH_3^{6\text{-}8}$ + CH_2), 0.84 (d, J = 6.4 Hz, 3 H, CH_3^{9}).

^{13}C NMR (75 MHz, DMSO-d_6): δ [ppm] = 168.50 (s, $CONR^{2}$), 164.91 (s, CO_2R^{34}), 151.71 (s, CO^{18}), 149.85 (s, C_{Ar}^{25}), 149.80 (s, CO_2R^{21}), 136.45 (s, C_{Ar}^{27}), 129.65 (s, $CH_{Ar}^{29,31}$), 128.69 (s, C_{Ar}^{30}), 128.26 (s, CH_{Ar}), 127.70 (s, CH_{Ar}), 127.63 (s, CH_{Ar}), 126.70 (s, $CH_{Ar}^{28,32}$), 98.05 (s, C^{15}), 74.02 (s, CH^{1}), 64.84 (s, CH_2^{24}), 53.89 (s, CH^{16}), 50.22 (s, C^{4}), 31.65 (s, CH_2), 31.13 (s, CH_2), 28.45 (s, $CH_3^{6\text{-}8}$), 28.34 (s, CH_2), 24.64 (s, CH_2), 21.99 (s, CH_2), 17.90 (s, CH_3^{26}), 13.92 (s, CH_3^{9}).

FAB – MS [*m/z*] (relative intensity): 564.3 (20%) [M + H]$^+$, 456.3 (15%) [M – C_7H_7O]$^+$, 349.1 (80%) [Fragment A]$^+$, 214.0 (10%) [Fragment B]$^+$.

HRMS – FAB [*m/z*]: [M + H]$^+$ calculated for $^{12}C_{32}{}^{1}H_{42}{}^{16}O_6{}^{14}N_3$, 564.3068; found, 564.3069; Δ = 0.09 mmu.

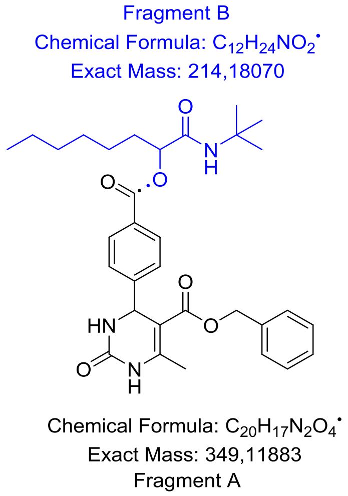

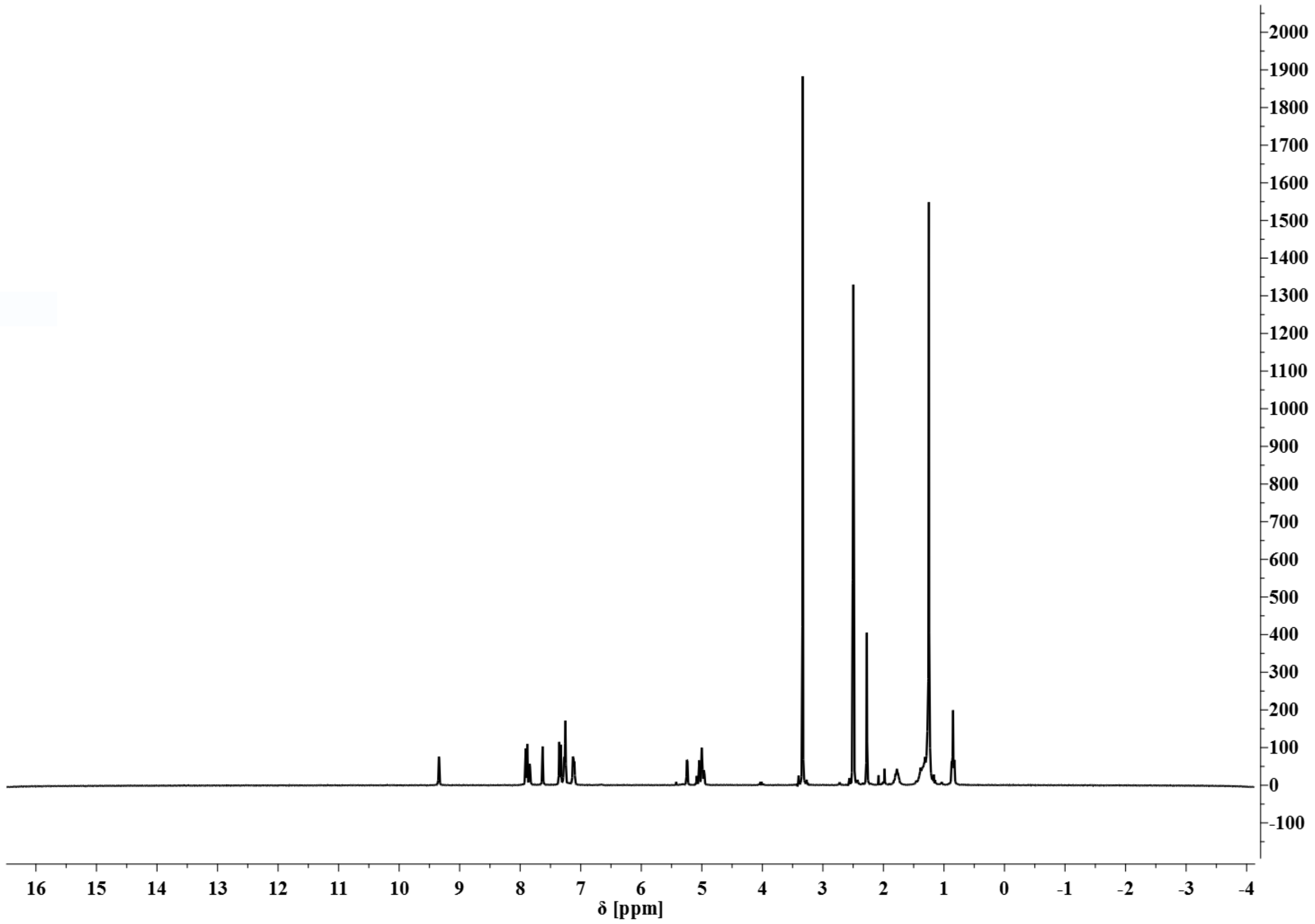

2000
1900
1800
1700
1600
1500
1400
1300
1200
1100
1000
900
800
700
600
500
400
300
200
100
0
-100
16 15 14 13 12 11 10 9 8 7 6 5 4 3 2 1 0 -1 -2 -3 -4
δ [ppm]

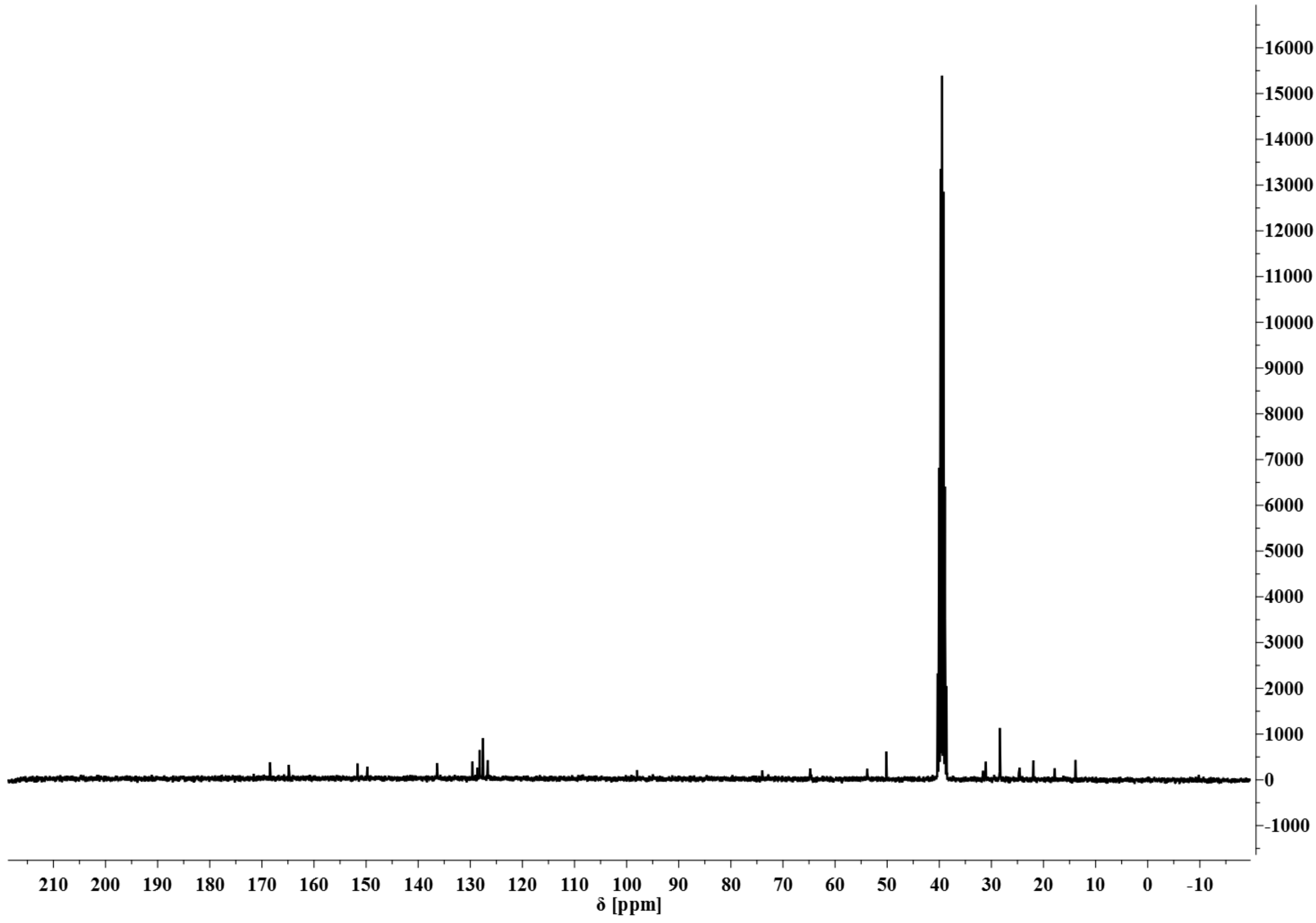

16000
15000
14000
13000
12000
11000
10000
9000
8000
7000
6000
5000
4000
3000
2000
1000
0
-1000
210 200 190 180 170 160 150 140 130 120 110 100 90 80 70 60 50 40 30 20 10 0 -10
δ [ppm]

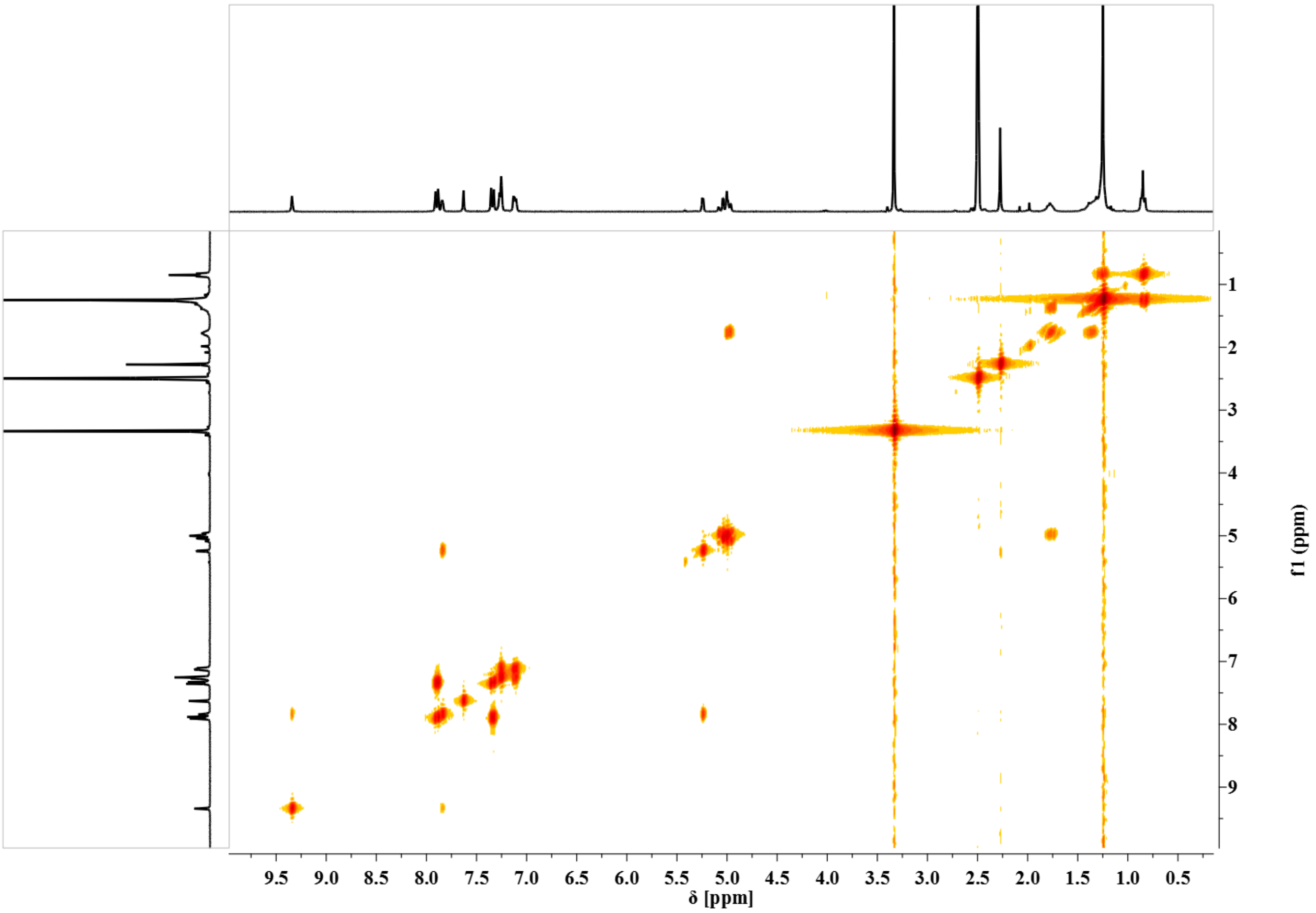

Passerini product 21 derived from Biginelli acid 17, cyclohexyl isocyanide and 2-phenylpropanal

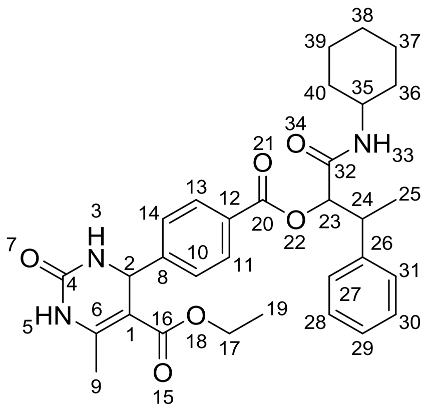

In a tube vial, finely powdered Biginelli acid **17** (300 mg, 986 µmol, 1.00 equiv) was dissolved in 0.3 mL dimethyl sulfoxide with 2.00 mL dichloromethane. Subsequently, 2-phenylpropanal (172 mg, 1.28 mmol, 1.30 equiv) and then cyclohexyl isocyanide (139 mg, 1.28 mmol, 1.30 equiv) were added. The mixture was stirred at room temperature for 3 d. Subsequently, the crude reaction mixture was concentrated under reduced pressure and purified via column chromatography employing silica gel and eluting with a gradual solvent mixture of ethyl

acetate/*c*-hexane (1:2 → 1:0). The Passerini product **21** was obtained as a colorless solid (536 mg, 969 µmol, 98.3%).

R_f = 0.37 in ethyl acetate

IR (ATR): ν [cm^{-1}] = 3270.2 (br, ν(N-H)), 2929.4 (w, ν(C-H)), 2853.0 (w, ν(C-H)), 1701.9 (vs, ν(C=O)), 1642.7 (vs, ν(C=O)), 1536.9 (w), 1449.3 (w), 1367.3 (w), 1266.6 (m), 1220.8 (vs, ν(COOR)), 1085.3 (vs, ν(COOR)), 1017.3 (m), 761.3 (m), 699.4 (s), 652.2 (w), 526.0 (w), 459.2 (w).

^{1}H NMR (600 MHz, DMSO-d_6): δ [ppm] = 9.26 (d, J = 14.0 Hz, 1 H, NH^5), 8.03 (t, J = 7.1 Hz, 0.5 H, NH^{33a}), 7.95 (dd, J = 8.3, 1.8 Hz, 1 H, $CH_{Ar}^{13,11a}$), 7.89 (d, J = 7.9 Hz, 0.5 H, NH^{33b}), 7.83 – 7.77 (m, 2 H, $CH_{Ar}^{13,11b}$ + NH^3), 7.38 (dd, J = 16.3, 7.9 Hz, 2 H, CH_{Ar}), 7.33 (d, J = 8.2 Hz, 2 H, CH_{Ar}), 7.31 – 7.25 (m, 2 H, CH_{Ar}), 7.20 – 7.16 (m, 1 H, CH_{Ar}), 5.24 – 5.17 (m, 1 H, CH^2), 5.14 (dd, J = 11.0, 3.6 Hz, 1 H, CH^{23}), 3.98 (dq, J = 14.0, 7.1 Hz, 2 H, CH_2^{17}), 3.58 – 3.45 (m, 1 H, CH^{35}), 3.43 – 3.28 (m, 1 H, CH^{24}), 2.28 – 2.21 (m, 3 H, CH_3^9), 1.63 (m, 5 H, $CH_2^{c\text{-}hex}$), 1.31 (dd, J = 46.8, 6.5 Hz, 3 H, CH_3^{25}), 1.20 (dt, J = 14.2, 7.7 Hz, 5 H, $CH_2^{c\text{-}hex}$), 1.13 – 1.04 (m, 3 H, CH_3^{19}).

^{13}C NMR (75 MHz, DMSO-d_6): δ [ppm] = 166.91 (s, CONR^{32}), 166.72 (s, CONR^{32}), 165.15 (s, CO_2R^{20}), 164.76 (s, CO_2R^{20}), 164.68 (s, CO_2R^{16}), 151.88 (s, CO^4), 151.83 (s, CO^4), 150.14 (s, C_{Ar}^{12}), 148.93 (s, C^6), 142.60 (s, C_{Ar}^{26}), 142.27 (s, C_{Ar}^{26}), 129.69 (s, CH_{Ar}), 129.52 (s, CH_{Ar}), 128.44 (s, CH_{Ar}), 128.30 (s, CH_{Ar}), 128.13 (s, CH_{Ar}), 128.10 (s, CH_{Ar}), 127.78 (s, CH_{Ar}), 126.64 (s, CH_{Ar}), 126.56 (s, CH_{Ar}), 98.57 (s, C^1), 98.51 (s, C^1), 78.18 (s, CH^{23}), 77.65 (s, CH^{23}), 59.26 (s, CH_2^{17}), 53.87 (s, CH^2), 47.46 (s, CH^{35}), 41.15 (s, CH^{24}), 40.98 (s, CH^{24}), 32.25 (s, $CH_2^{c\text{-}hex}$), 32.13 (s, $CH_2^{c\text{-}hex}$), 25.14 (s, $CH_2^{c\text{-}hex}$), 24.56 (s, $CH_2^{c\text{-}hex}$), 24.47 (s, $CH_2^{c\text{-}hex}$), 17.79 (s, CH_3^9), 17.70 (s, CH_3^{25}), 17.66 (s, CH_3^{25}), 14.87 (s, CH_3^{25}), 14.80 (s, CH_3^{25}), 14.06 (s, CH_3^{19}), 14.04 (s, CH_3^{19}).

FAB – MS [*m/z*] (relative intensity): 548.3 (40%) $[M + H]^+$, 502.2 (5%) $[M - C_2H_5O]^+$, 303.1 (5%) [Fragment C]$^+$, 287.1 (100%) [Fragment A – H]$^+$, 244.1 (20%) [Fragment B]$^+$, 183.1 (20%) [Fragment D]$^+$.

HRMS – FAB [*m/z*]: $[M + H]^+$ calculated for $^{12}C_{31}{}^{1}H_{38}{}^{16}O_6{}^{14}N_3$, 548.2755; found, 548.2755; Δ = 0.05 mmu.

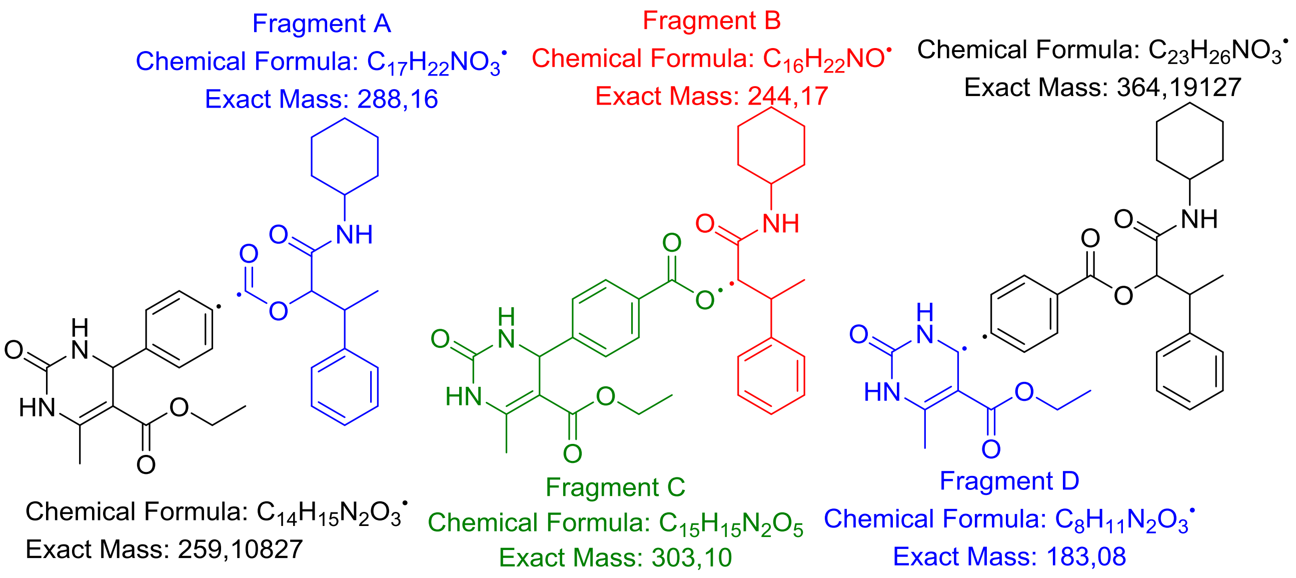
Fragment A
Chemical Formula: C17H22NO3•
Exact Mass: 288,16
Fragment B
Chemical Formula: C16H22NO•
Exact Mass: 244,17
Chemical Formula: C23H26NO3•
Exact Mass: 364,19127
Chemical Formula: C14H15N2O3•
Exact Mass: 259,10827
Fragment C
Chemical Formula: C15H15N2O5
Exact Mass: 303,10
Fragment D
Chemical Formula: C8H11N2O3•
Exact Mass: 183,08

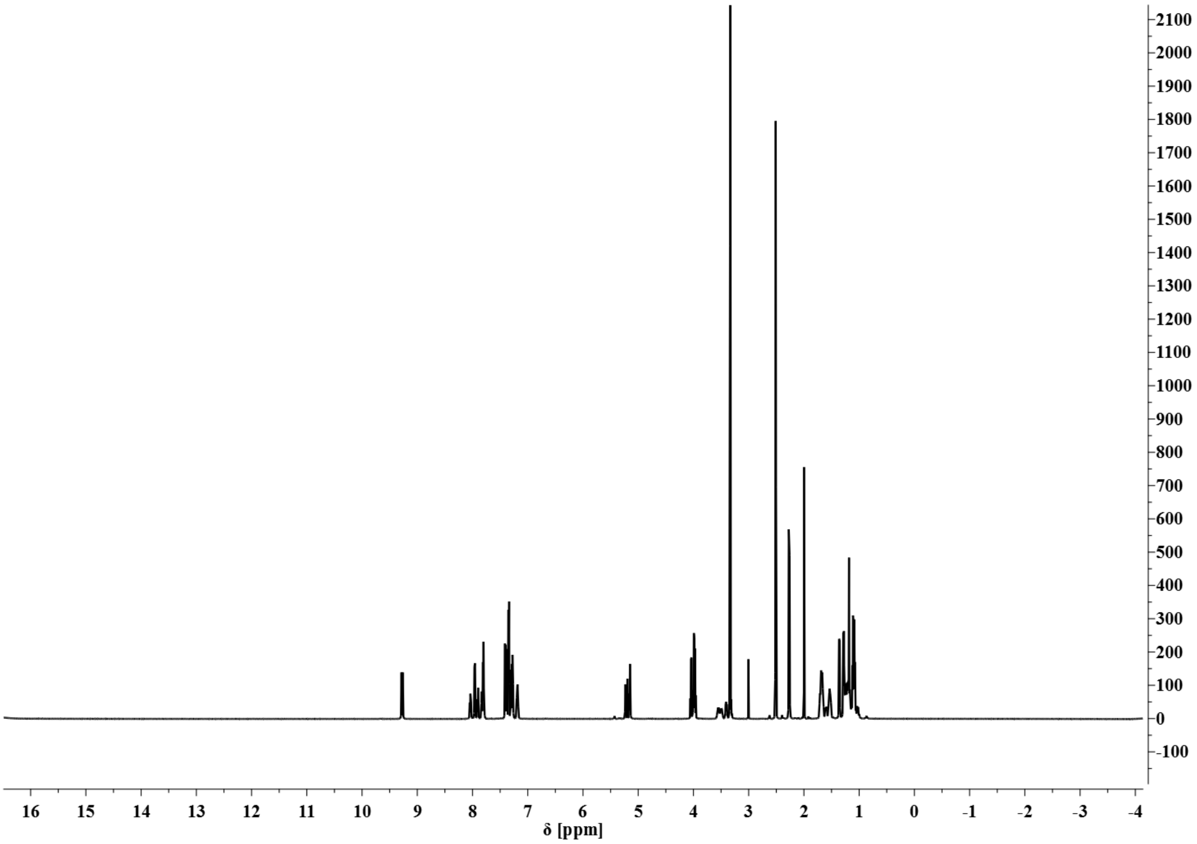
δ [ppm]

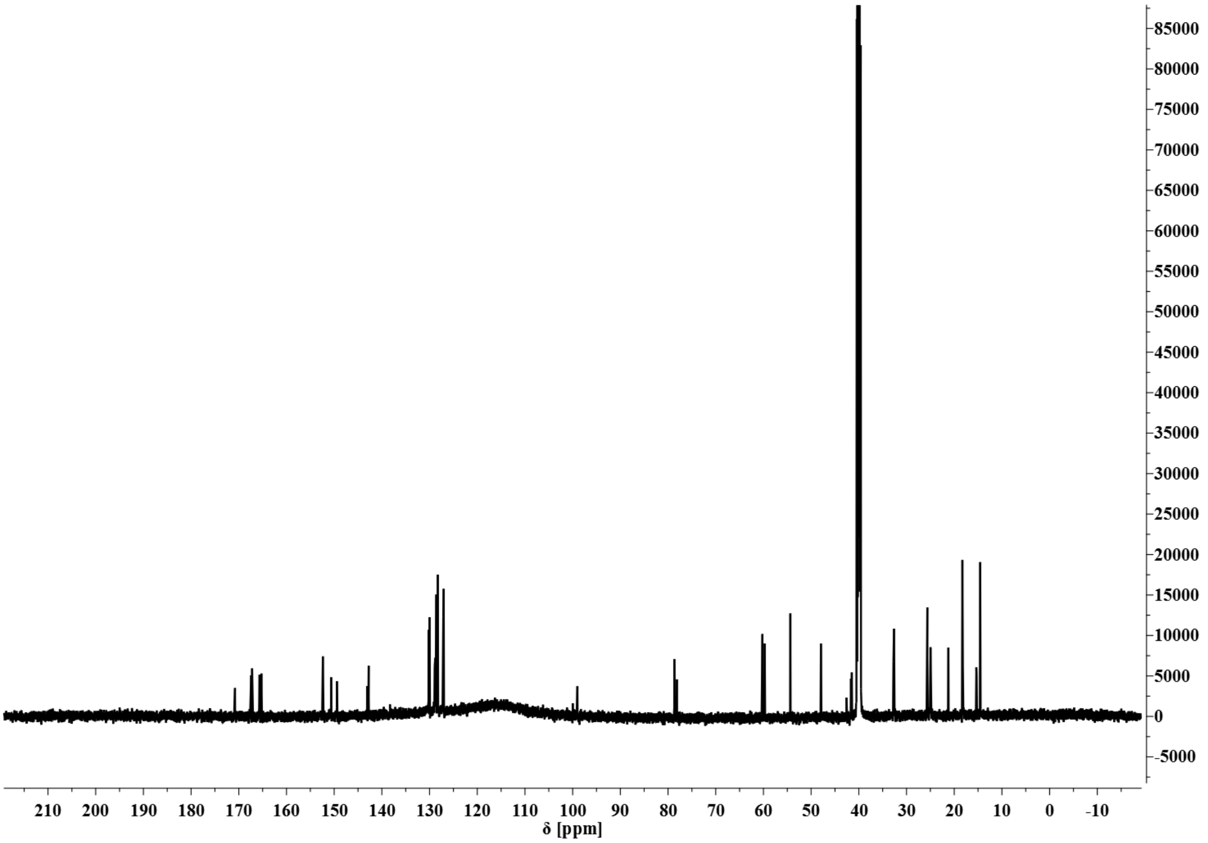

85000
80000
75000
70000
65000
60000
55000
50000
45000
40000
35000
30000
25000
20000
15000
10000
5000
0
-5000
210 200 190 180 170 160 150 140 130 120 110 100 90 80 70 60 50 40 30 20 10 0 -10
δ [ppm]

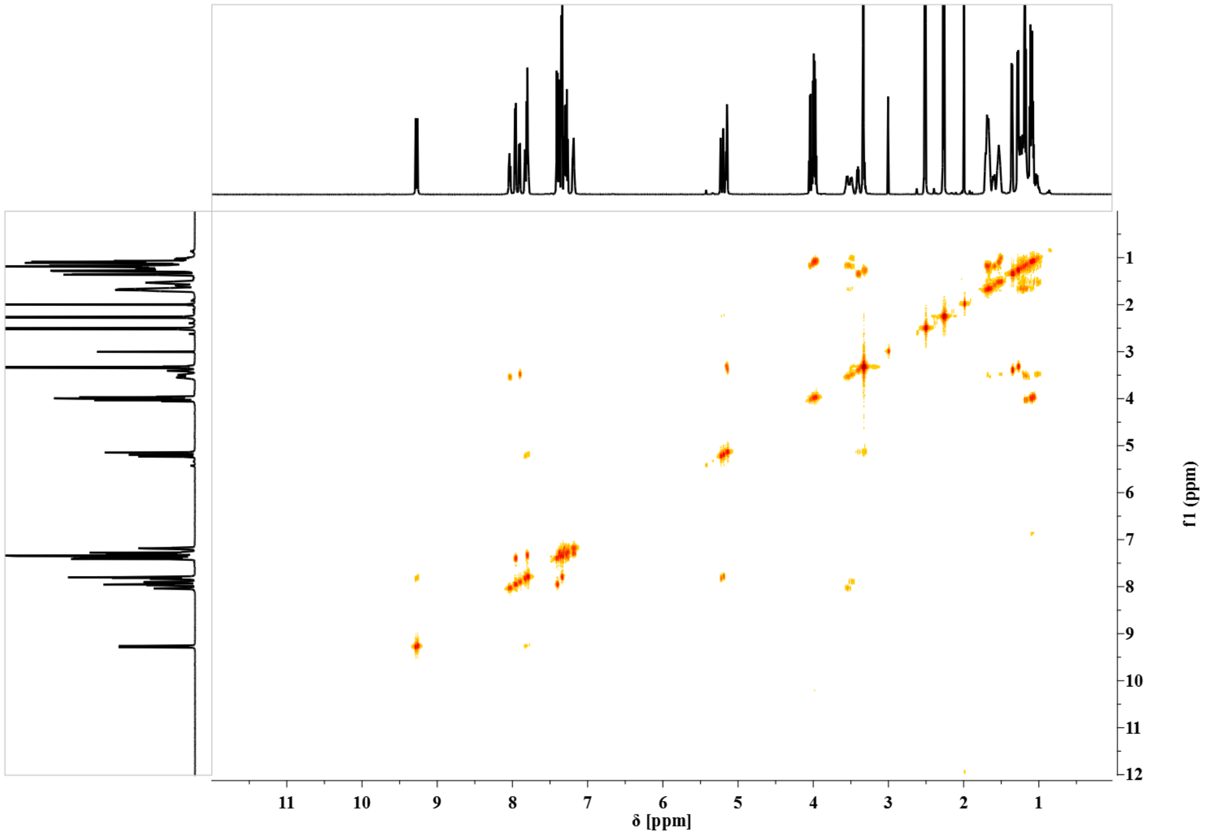

1 2 3 4 5 6 7 8 9 10 11 12
f1 (ppm)
11 10 9 8 7 6 5 4 3 2 1
δ [ppm]

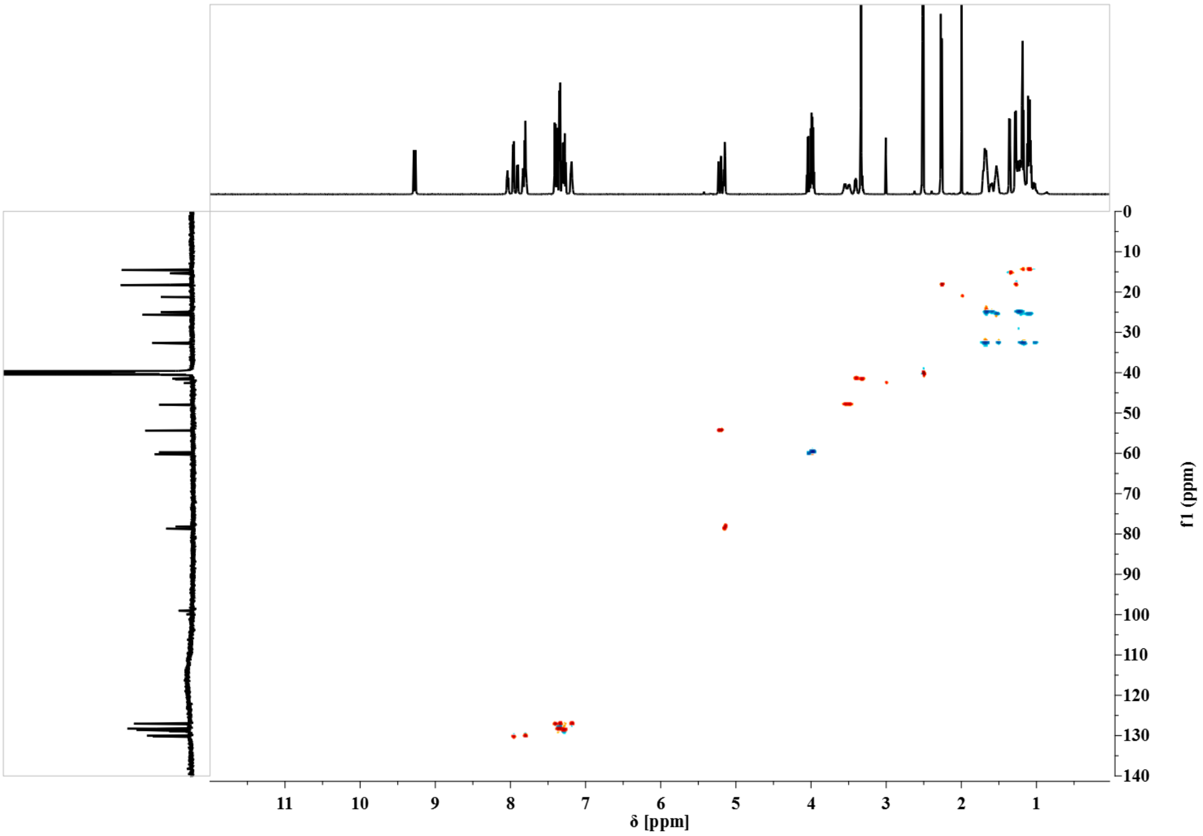
δ [ppm]
f1 (ppm)

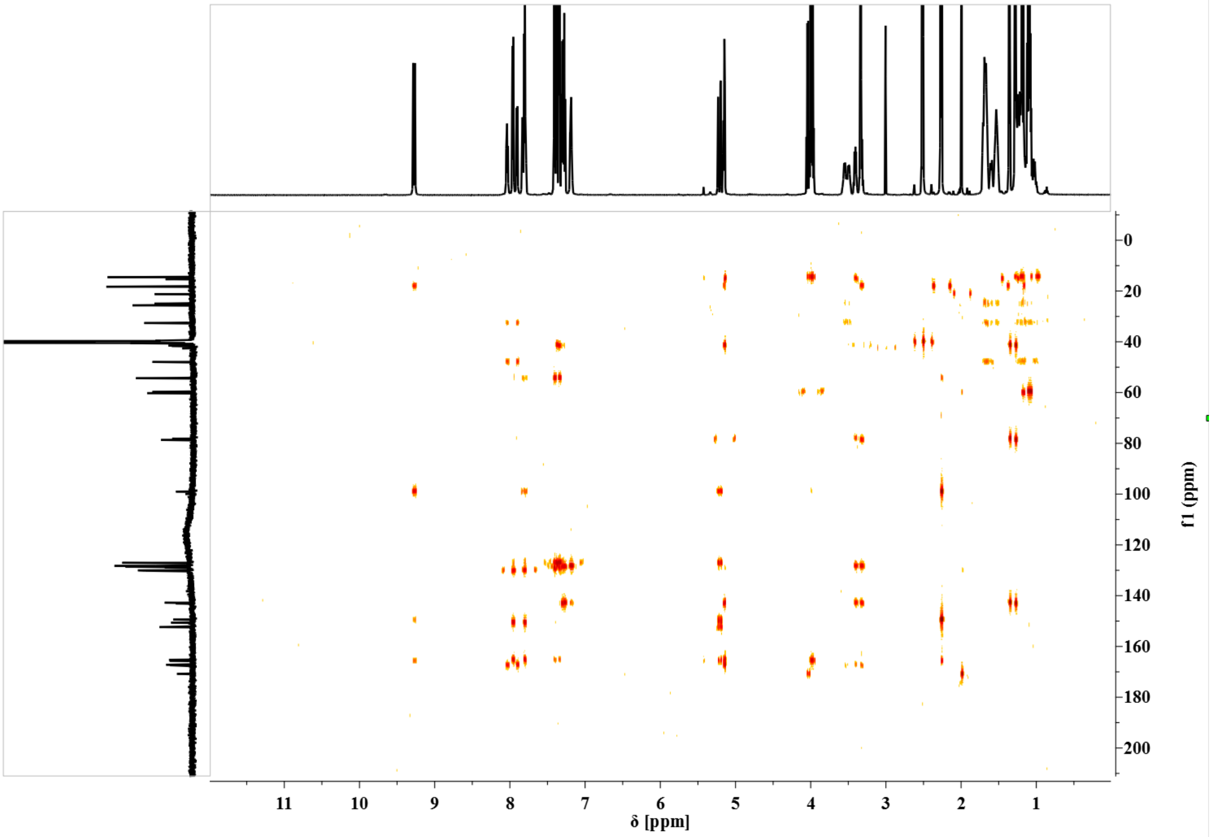
δ [ppm]
f1 (ppm)

Passerini product 22 derived from Biginelli acid 15, isobutylaldehyde and *tert*-butyl isocyanide

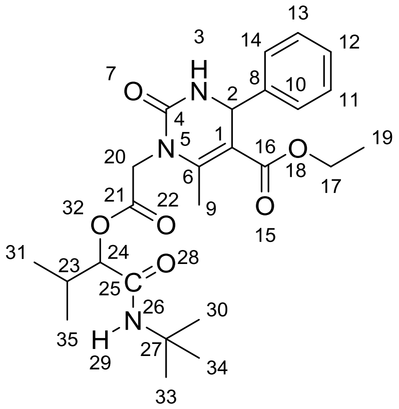

In a 5 mL round-bottomed flask, finely powdered Biginelli acid **15** (500 mg, 1.57 mmol, 1.20 equiv) was dissolved in a minimal amount of dimethyl sulfoxide (0.5 mL). Subsequently, *iso*-butyraldehyde (123 mg, 1.73 mmol, 157 µL, 1.10 equiv) and *tert*-butyl isocyanide (131 mg, 1.57 mmol, 150 µL) were added. The mixture was diluted with dichloromethane (0.5 mL) and stirred at room temperature for 6 h. Subsequently, a second portion of aldehyde (56.6 mg, 0.50 equiv) and isocyanide (65.3 mg, 0.50 equiv) were added. The resulting mixture was stirred for 3 d at room temperature. The crude reaction mixture was added dropwise into a stirred mixture of 50 mL water and 50 mL dichloromethane. The organic phase was separated, dried over sodium sulfate and concentrated under reduced pressure. The solid residue was purified via column chromatography on silica gel eluting with a gradual solvent mixture of *c*-hexane and diethyl ether (1:0 → 0:1). The Passerini product **22** was obtained as a colorless solid (563 mg, 1.18 µmol, 75.6%).

IR (ATR): ν [cm^{-1}] = 3316.3 (br, ν(O-H)), 2964.6 (m, ν(C-H)), 2161.3 (w), 1751.9 (w, ν(C=O)), 1681.3 (vs, ν(C=O)), 1528.6 (s), 1454.1 (s), 1386.6 (m), 1309.8 (w), 1178.0 (vs), 1104.3 (s), 1056.6 (m), 1015.2 (m), 940.6 (w), 760.3 (w), 698.2 (m).

^{1}H NMR (400 MHz, DMSO-d_6): δ [ppm] = 8.18 (dd, J = 15.1, 3.7 Hz, 1 H, NH^{3}), 7.42 (d, J = 32.9 Hz, 1 H, NH^{29}), 7.34 – 7.22 (m, 5 H, $CH_{Ar}^{10\text{-}14}$), 5.20 (t, J = 3.8 Hz, 1 H, CH^{2}), 4.71 – 4.40 (m, 3 H, CH^{24} + CH_2^{20}), 4.02 (q, J = 7.0, 2 H, CH_2^{17}), 2.41 (s, 3 H, CH_3^{9}), 2.15 – 1.90 (m, 1 H, CH^{23}), 1.28 – 1.24 (m. 10 H, $CH_3^{30,33,34}$), 1.10 (t, J = 7.1 Hz, 3 H, CH_3^{19}), 0.91 – 0.82 (m, 6 H, $CH_3^{31,35}$), 0.74 (d, J = 6.8 Hz, 1 H).

^{13}C NMR (75 MHz, DMSO-d_6): δ [ppm] = 172.59 (s), 169.00 (s, CO_2R^{21}), 168.98 (s, CO_2R^{21}), 167.45 (s, $CONR^{25}$), 165.50 (s, CO_2R^{16}), 165.44 (s, CO_2R^{16}), 152.60 (s, CO^{4}), 152.43 (s, CO^{4}), 149.06 (s, C^{6}), 148.80 (s, C^{6}), 143.98 (s, C_{Ar}^{8}), 143.83 (s, C_{Ar}^{8}), 128.41 (s, $CH_{Ar}^{10,14}$), 128.40 (s, $CH_{Ar}^{10,14}$), 127.46 (s, CH_{Ar}^{12}), 127.44 (s, CH_{Ar}^{12}), 126.43 (s, $CH_{Ar}^{11,13}$), 126.38 (s, $CH_{Ar}^{11,13}$),

103.25 (s, C^{1}), 103.12 (s, C^{1}), 78.52 (s, CH^{24}), 78.17 (s, CH^{24}), 59.72 (s, CH_2^{17}), 59.68 (s, CH_2^{17}), 53.02 (s, CH^{2}), 52.93 (s, CH^{2}), 50.39 (s, C^{27}), 50.30 (s, C^{27}), 49.74 (s, C), 44.18 (s, CH_2^{20}), 31.24 (s), 30.04 (s, CH^{23}), 29.88 (s, CH^{23}), 28.46 (s, $CH_3^{30,33,34}$), 28.37 (s, $CH_3^{30,33,34}$), 28.35 (s, $CH_3^{30,33,34}$), 19.21 (s, $CH_3^{31,35}$), 18.59 (s, $CH_3^{31,35}$), 18.55 (s, $CH_3^{31,35}$), 16.95 (s, $CH_3^{31,35}$), 16.78 (s, $CH_3^{31,35}$), 15.54 (s, CH_3^{9}), 15.50 (s, CH_3^{9}), 13.95 (s, CH_3^{19}).

FAB – MS [*m/z*] (relative intensity): 474.3 (100%) $[M + H]^+$, 428.2 (30%) $[M – C_2H_5O]^+$. HRMS – FAB [*m/z*]: $[M + H]^+$ calculated for $^{12}C_{25}{}^{1}H_{36}{}^{16}O_{6}{}^{14}N_{3}$, 474.2599; found, 474.2600; Δ = 0.11 mmu.

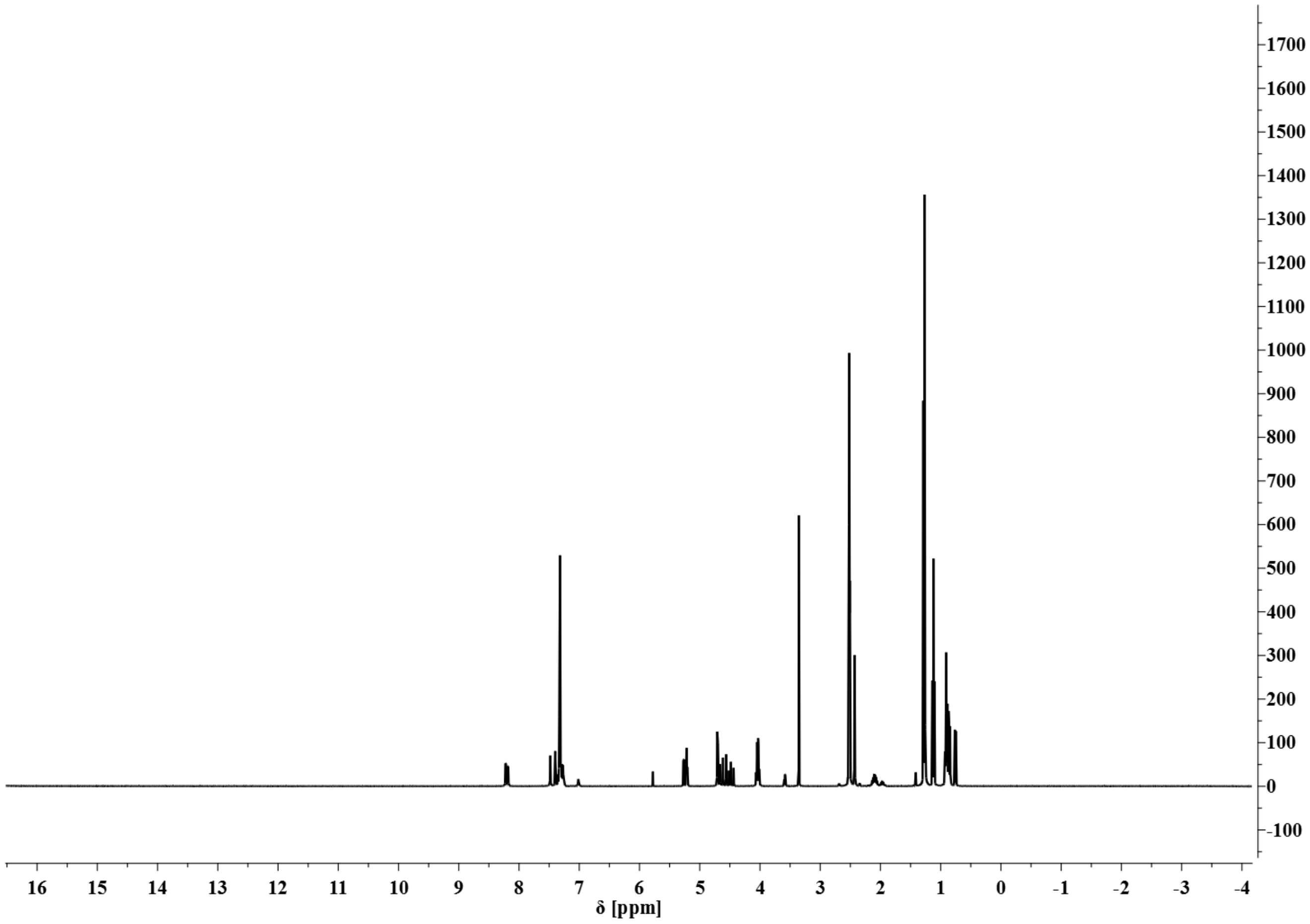

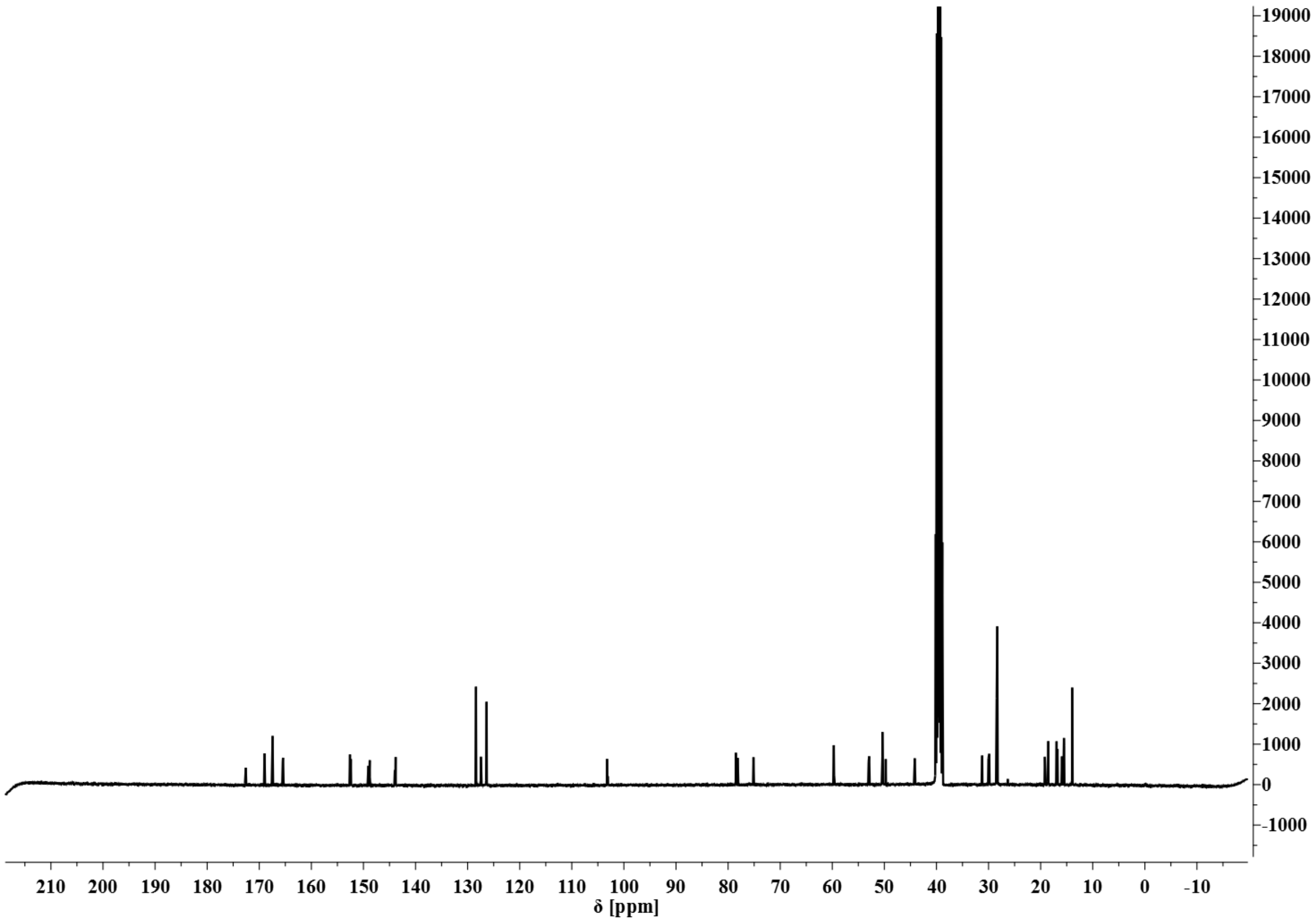
δ [ppm]

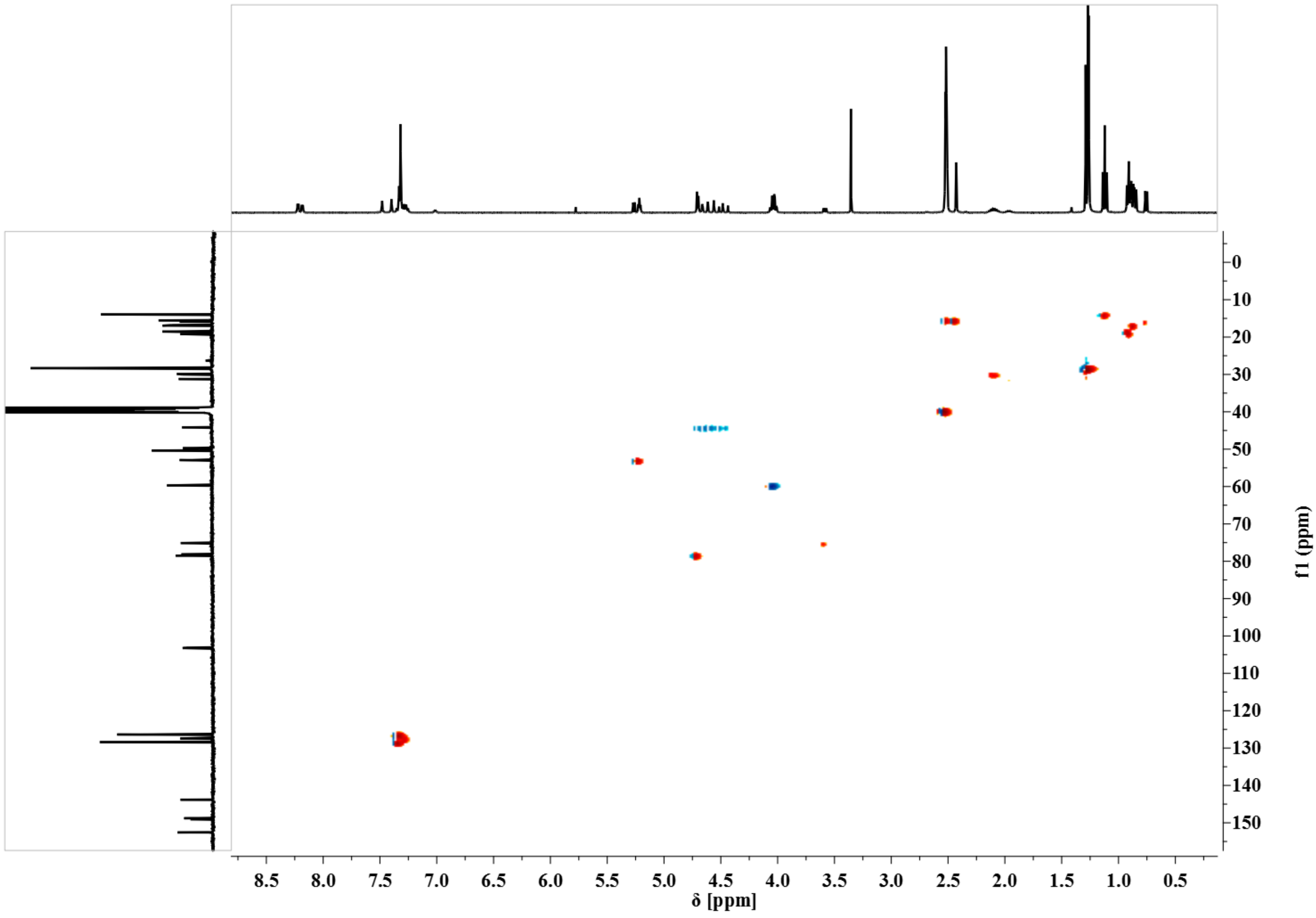
δ [ppm]
f1 (ppm)

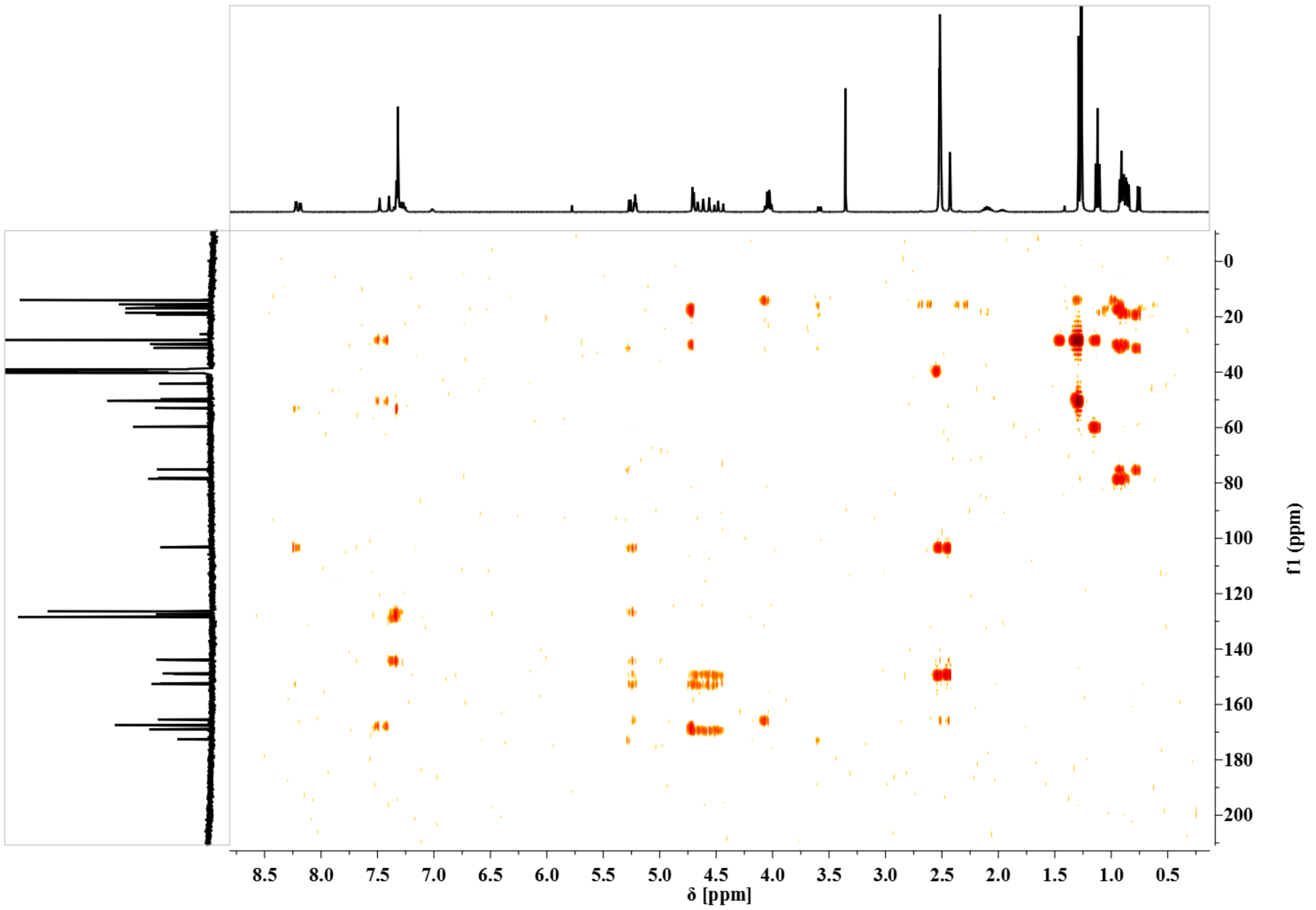

Passerini product 23 derived from Biginelli acid 15, undec-10-enal and *tert*-butyl isocyanide

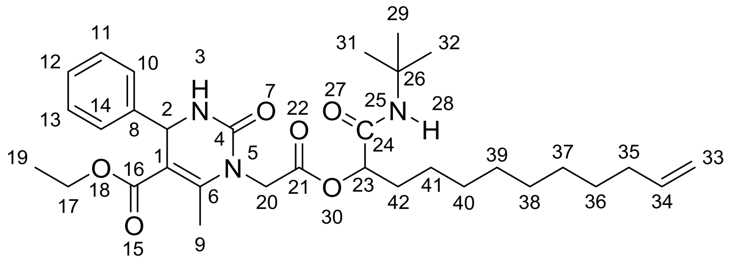

In a 5 mL round-bottomed flask, finely powdered Biginelli acid **15** (500 mg, 1.57 mmol, 1.20 equiv) was dissolved in 1 mL dimethyl sulfoxide/dichloromethane (1:1). Subsequently, undec-10-enal (344 mg, 2.04 mmol, 409 µL, 1.30 equiv) and *tert*-butylisocyanide (170 mg, 2.04 mmol, 196 µL) were added. The mixture was diluted with dichloromethane (0.5 mL) and stirred at room temperature for 8 h. Subsequently, a second portion of aldehyde (79.3 mg, 0.30 equiv) and ioscyanide (39.2 mg, 0.30 equiv) were added to the mixture and stirred at room temperature for 3 d. The crude reaction mixture was added dropwise into a stirred emulsion of 50 mL water and 50 mL dichloromethane. The organic phase was separated, dried over sodium sulfate and dried under reduced pressure. The oily residue was purified via column

chromatography on silica gel eluting with a gradual solvent mixture of *c*-hexane and diethyl ether (0:1 → 1:0). The Passerini product **23** was obtained as a colorless oil (893 mg, 1.56 mmol, 99.7%).

IR (ATR): ν [cm^{-1}] = 3323.2 (br, ν(N-H)), 2924.7 (m, ν(C-H)), 2853.1 (w, ν(C-H)), 1752.5 (w, ν(C=O)), 1681.5 (vs, ν(C=O)), 1529.2 (m), 1453.7 (m), 1385.8 (m), 1364.8 (m), 1309.4 (w), 1278.1 (w), 1257.7 (w), 1176.3 (vs), 1103.6 (s), 1056.2 (m), 941.7 (w), 909.0 (w), 861.3 (w), 828.5 (w), 759.6 (m), 697.5 (s), 651.6 (w).

^{1}H NMR (400 MHz, DMSO-d_6): δ [ppm] = 8.16 (dd, J = 16.9, 3.6 Hz, 1 H, NH^{3}), 7.43 (d, J = 11.2 Hz, 1 H, NH^{28}), 7.34 – 7.21 (m, 5 H, $CH_{Ar}^{10\text{-}14}$), 5.84 – 5.73 (m, 1 H, CH^{34}), 5.19 (t, J = 3.5 Hz, 1 H, CH^{2}), 5.02 – 4.91 (m, 2 H, CH_2^{33}), 4.84 (t, J = 6.3 Hz, 1 H, CH^{23}), 4.74 – 4.40 (m, 2 H, CH_2^{20}), 4.06 – 3.96 (m, 2 H, CH_2^{17}), 2.43 (d, J = 17.4 Hz, 3 H, CH_3^{9}), 2.00 (q, J = 6.9 Hz, 2 H, CH_2^{35}), 1.69 – 1.61 (m, 2 H, CH_2^{42}), 1.27 – 1.21 (m, 21 H, $CH_3^{29,31,32}$ + $CH_2^{36\text{-}41}$), 1.14 – 1.07 (t, J = 7.1 Hz, 3 H, CH_3^{19}).

^{13}C NMR (100 MHz, DMSO-d_6): δ [ppm] = 168.95 (s, CO_2R^{21}),), 168.88 (s, CO_2R^{21}),), 168.14 (s, $CONR^{24}$), 165.43 (s, CO_2R^{16}), 165.41 (s, CO_2R^{16}), 152.49 (s, CO^{4}), 152.27 (s, CO^{4}), 148.84 (s, C^{6}), 148.76 (s, C^{6}), 144.05 (s, C_{Ar}^{8}), 143.87 (s, C_{Ar}^{8}), 138.79 (s, CH^{34}), 128.38 (s, $CH_{Ar}^{10,14}$), 128.34 (s, $CH_{Ar}^{10,14}$), 127.41 (s, CH_{Ar}^{12}), 126.49 (s, $CH_{Ar}^{11,13}$), 126.38 (s, $CH_{Ar}^{11,13}$), 114.60 (s, CH_2^{33}), 103.21 (s, C^{1}), 103.19 (s, C^{1}), 74.31 (s, CH^{23}), 74.16 (s, CH^{23}), 59.65 (s, CH_2^{17}), 53.15 (s, CH^{2}), 52.95 (s, CH^{2}), 50.30 (s, C^{26}), 50.26 (s, C^{26}), 44.03 (s, CH_2^{20}), 33.17 (s, CH_2^{35}), 31.37 (s, CH_2^{42}), 31.28 (s, CH_2^{42}), 28.79 (s, CH_2), 28.75 (s, CH_2), 28.65 (s, CH_2), 28.54 (s, CH_2), 28.51 (s, CH_2), 28.45 (s), 28.34 (s, $CH_3^{29,31,32}$), 28.24 (s, CH_2^{41}), 26.32 (s, CH_2^{41}), 24.31 (s,CH_2), 24.27 (s, CH_2^{41}), 15.53 (s, CH_3^{9}), 15.48 (s, CH_3^{9}), 13.93 (s, CH_3^{19}).

FAB – MS [*m/z*] (relative intensity): 570.4 (55%) $[M + H]^+$, 524.3 (35%) $[M - C_2H_5O]^+$, 492.3 (10%) $[M - C_6H_5]^+$, 317.1 (65%) [Fragment A]$^+$, 273.1 (35%) [Fragment B]$^+$.

HRMS – FAB [*m/z*]: $[M + H]^+$ calculated for $^{12}C_{32}{}^{1}H_{48}{}^{16}O_6{}^{14}N_3$, 570.3538; found, 570.3538; Δ = 0.04 mmu.

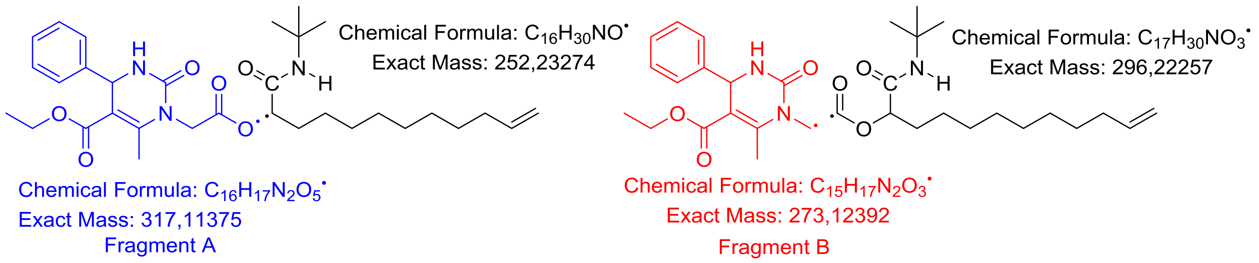

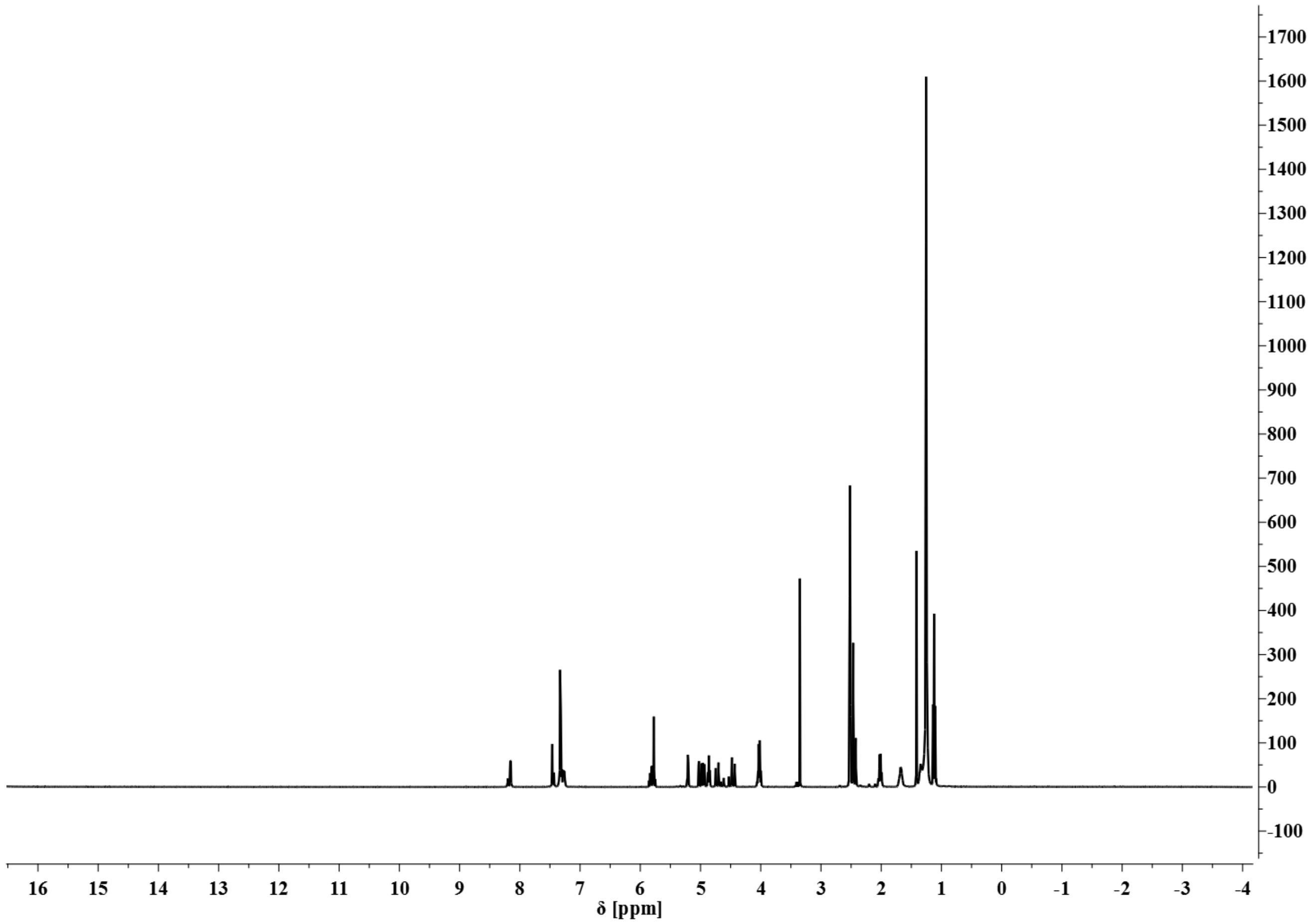
1700
1600
1500
1400
1300
1200
1100
1000
900
800
700
600
500
400
300
200
100
0
-100
16 15 14 13 12 11 10 9 8 7 6 5 4 3 2 1 0 -1 -2 -3 -4
δ [ppm]

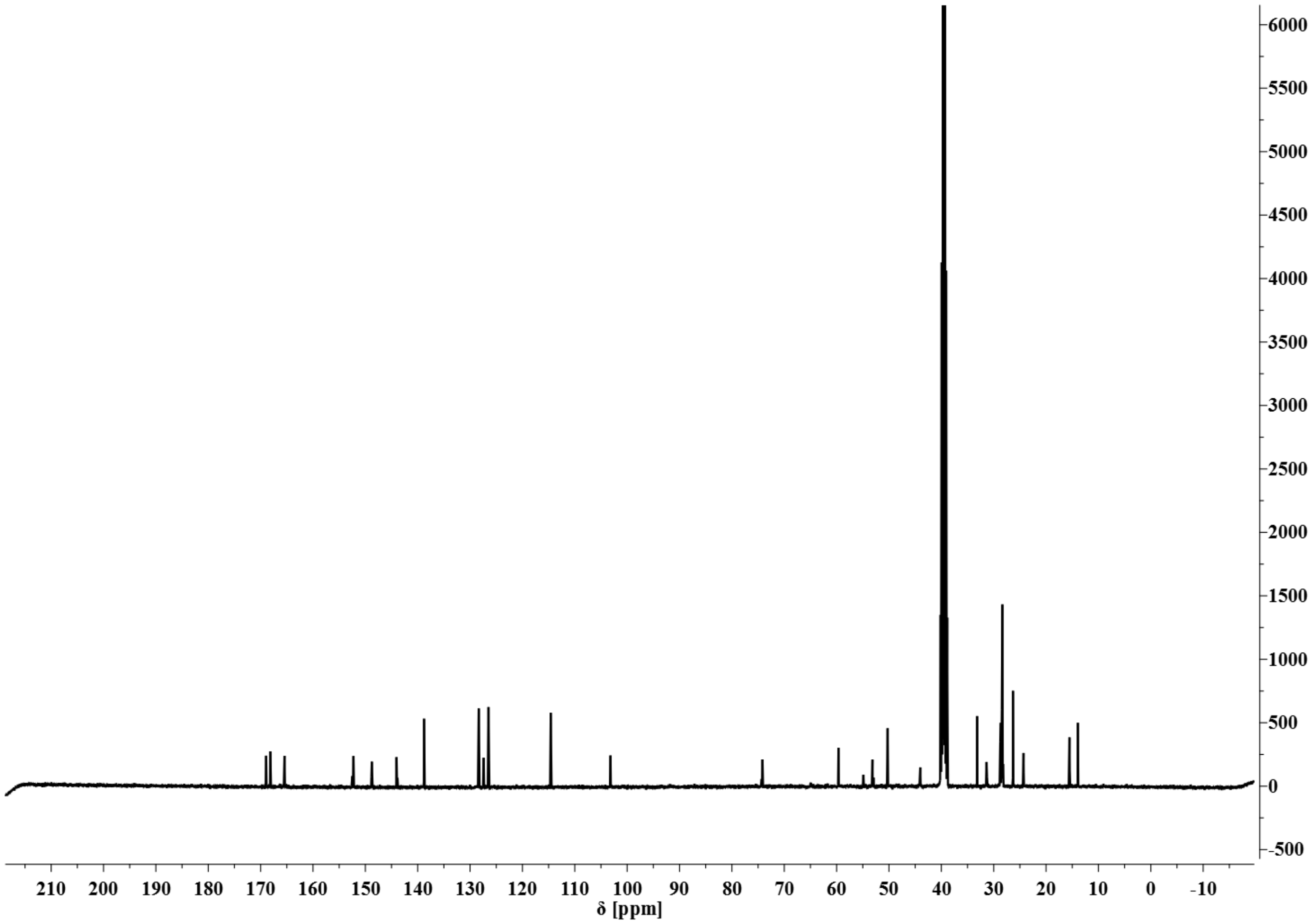
6000
5500
5000
4500
4000
3500
3000
2500
2000
1500
1000
500
0
-500
210 200 190 180 170 160 150 140 130 120 110 100 90 80 70 60 50 40 30 20 10 0 -10
δ [ppm]

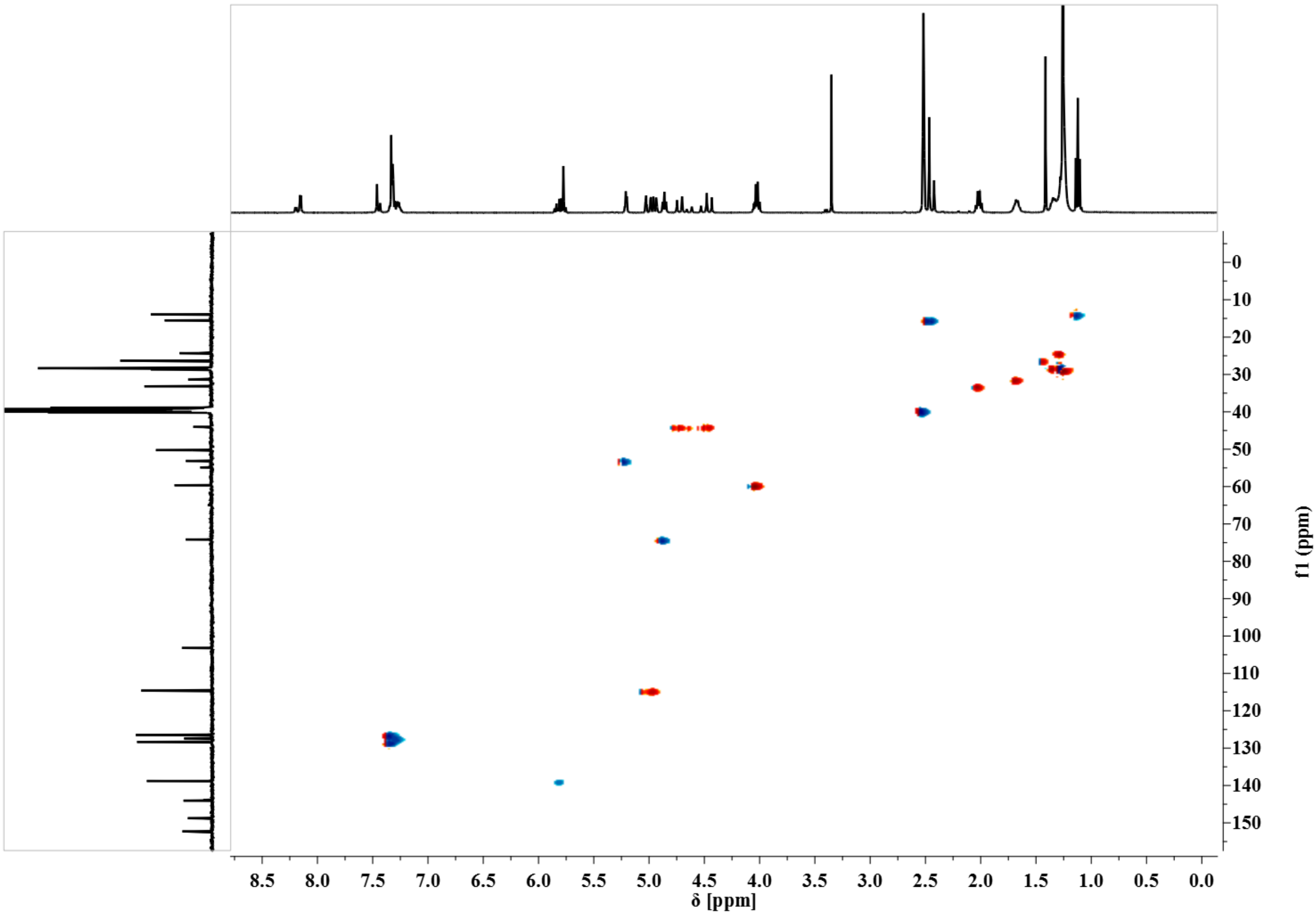
δ [ppm]
f1 (ppm)

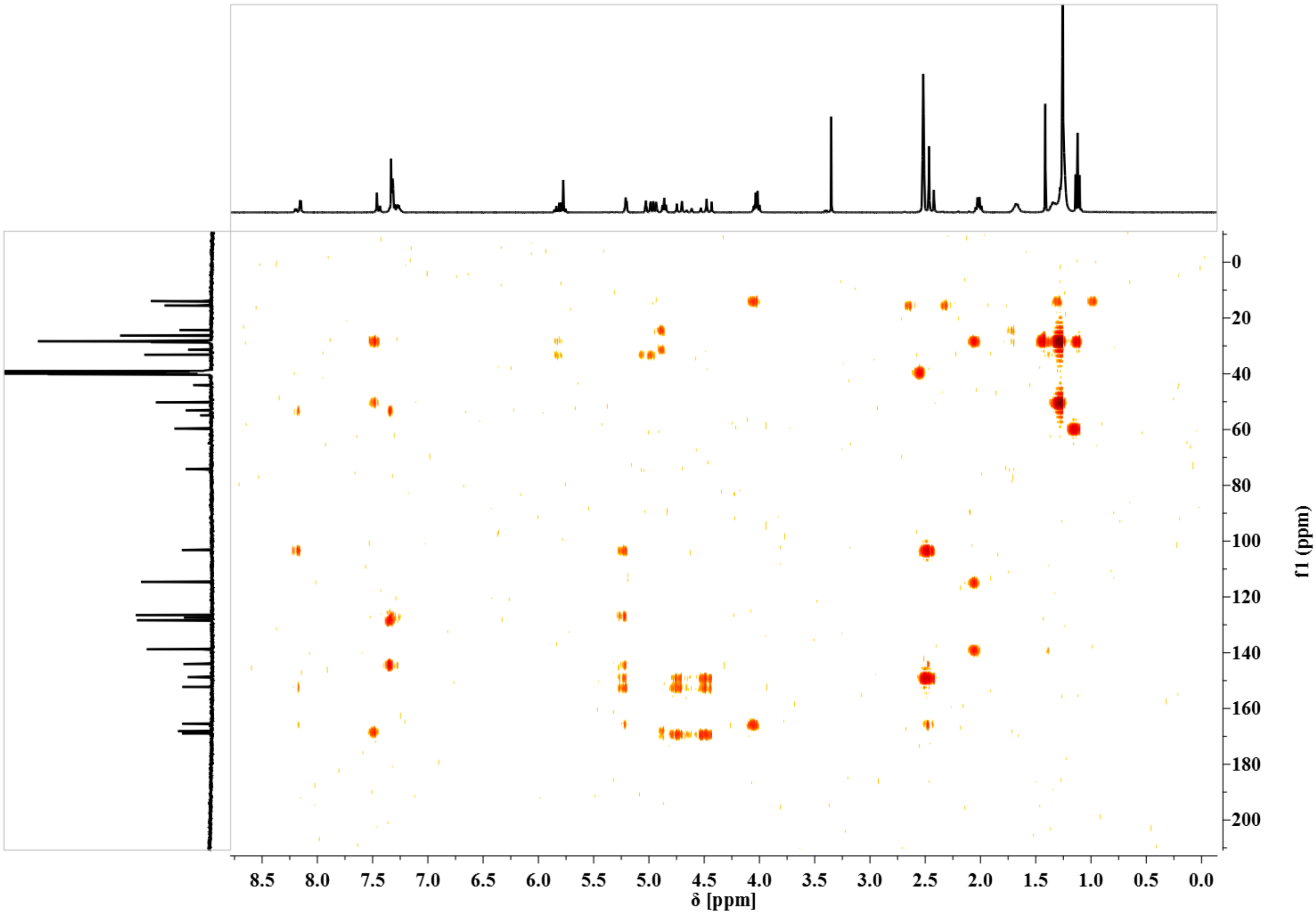
δ [ppm]
f1 (ppm)

Passerini compound 24 derived from Biginelli acid 15, octanal and benzyl isocyanide.

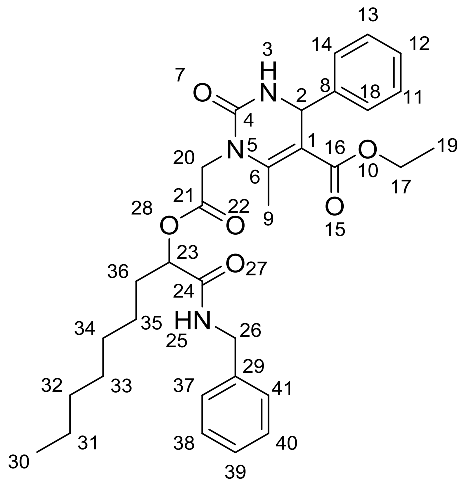

In a tube vial, finely powdered Biginelli acid **15** (302 mg, 949 µmol, 1.00 equiv) was dissolved in 0.3 mL dimethyl sulfoxide and diluted with 1.0 mL dichloromethane. Subsequently, octanal (178 mg, 1.38 mmol, 217 µL, 1.40 equiv) and benzyl isocyanide (150 mg, 1.28 mmol, 156 µL, 1.30 equiv) were added. The mixture was stirred at room temperature for 3 d. The crude product was purified via column chromatography employing silica gel and eluting with a gradual solvent mixture of ethyl acetate and *c*-hexane (1:9 → 1:0). The Passerini product **24** was obtained as a white solid (405 mg, 715 µmol, 76.2%).

IR (ATR): ν [cm^{-1}] = 3279.1 (br, ν(N-H)), 2922.5 (w, ν(C-H)), 2852.7 (w, ν(C-H)), 1737.0 (w, ν(C=O)), 1709.5 (m, ν(C=O), 1681.3 (vs, ν(C=O)), 1662.1 (vs, ν(C=O)), 1613.8 (s, ν(C=O)), 1541.4 (m), 1495.5 (w), 1453.4 (m), 1405.5 (m), 1373.3 (m), 1306.1 (m), 1278.8 (w), 1253.4 (w), 1189.9 (vs, ν(COOR), 1103.6 (m), 1080.3 (m), 1053.5 (s), 936.8 (m), 859.4 (w), 821.1 (m), 763.8 (m), 695.3 (s), 607.3 (w), 482.9 (w), 407.5 (w).

^{1}H NMR (500 MHz, DMSO-d_6): δ [ppm] = 8.55 (t, J = 5.9 Hz, 1 H, NH^{25}), 8.17 (d, J = 3.2 Hz, 1 H, NH^{3}), 7.33 – 7.28 (m, 6 H, CH_{Ar}), 7.27 – 7.22 (m, 4 H, CH_{Ar}), 5.21 (d, J = 3.2 Hz, 1 H, CH^{2}), 5.04 – 4.97 (m, 1 H, CH^{23}), AB-signal (δ A = 4.73, δ B = 4.51, J_{AB} = 17.5 Hz, 2 H, CH_2^{20}), 2 H, CH_2^{20}), 4.31 (d, J = 5.9 Hz, 2 H, CH_2^{26}), 4.02 (q, J = 7.0 Hz, 2 H, CH_2^{17}), 2.39 (s, 3 H, CH_3^{9}), 1.79 – 1.70 (m, 2 H, CH_2^{36}), 1.34 – 1.20 (m, 13 H, CH_2), 1.11 (t, J = 7.1 Hz, 3 H, CH_3^{19}), 0.85 (t, J = 6.8 Hz, 3 H, CH_3^{30}).

^{13}C NMR (126 MHz, DMSO-d_6): δ [ppm] = 169.24 (s, CO_2R^{21}), 169.01 (s, $CONR^{24}$), 165.43 (s, CO_2R^{16}), 152.44 (s, CO^{4}), 148.75 (s, C^{1}), 143.91 (s, C_{Ar}^{8}), 139.79 (s, C_{Ar}^{29}), 139.11 (s), 128.39 (s, CH_{Ar}), 128.21 (s, CH_{Ar}), 128.12 (s, CH_{Ar}), 127.42 (s, CH_{Ar}), 127.14 (s, CH_{Ar}), 126.98 (s, CH_{Ar}), 126.76 (s, CH_{Ar}), 126.60 (s, CH_{Ar}), 126.42 (s, CH_{Ar}), 103.26 (s, C^{1}), 74.13 (s, CH^{23}), 71.00 (s, CH), 59.69 (s, CH_2^{17}), 53.01 (s, CH^{2}), 44.05 (s, CH_2^{20}), 41.83 (s, CH_2^{26}), 31.31 (s,

CH_2), 31.19 (s, CH_2), 31.08 (s, CH_2), 28.85 (s, CH_2), 28.66 (s, CH_2), 28.49 (s, CH_2), 24.58 (s, CH_2), 24.37 (s, CH_2), 22.07 (s, CH_2), 22.04 (s, CH_2), 15.57 (s, CH_3^{9}), 13.95 (s, $CH_3^{30\ or\ 19}$), 13.90 (s, $CH_3^{30\ or\ 19}$).

FAB – MS [*m/z*] (relative intensity): 564.3 (90%) $[M + H]^+$, 518.3 (35%) $[M - C_2H_5O]^+$, 317.1 (50%) [Fragment A]$^+$, 301.1 (35%) [Fragment A – O]$^+$, 273.1 (15%) [Fragment C]$^+$, 259.1 (50%) [Fragment C – CH_2]$^+$, 246.2 (15%) [Fragment B]$^+$

HRMS – FAB [*m/z*]: $[M + H]^+$ calculated for $^{12}C_{23}{}^{1}H_{25}{}^{16}O_{6}{}^{14}N_{2}$, 425.1707; found, 425-1705; Δ = 0.05 mmu.

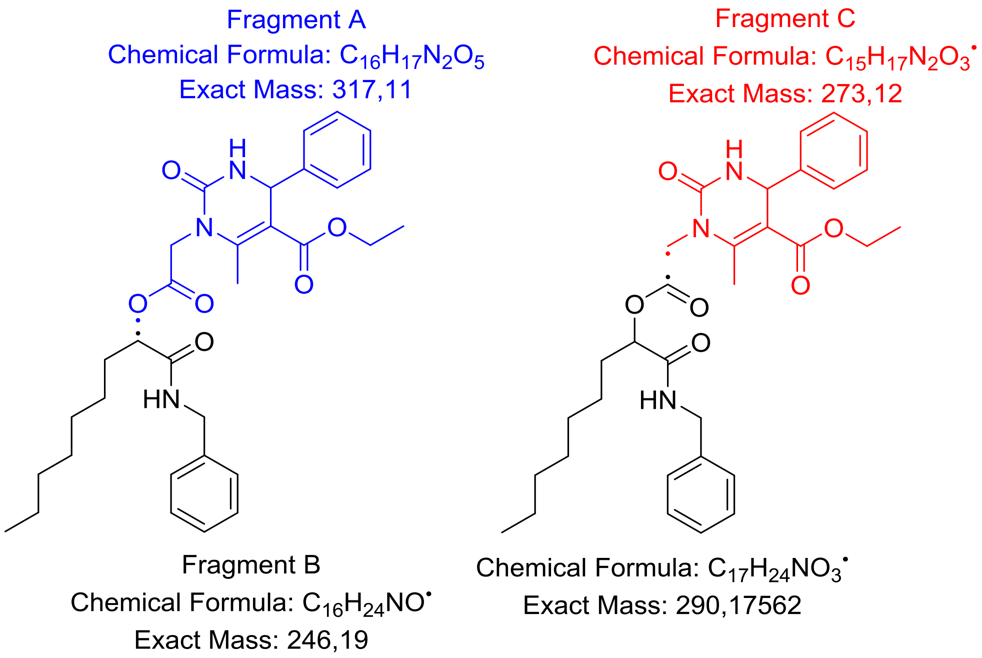

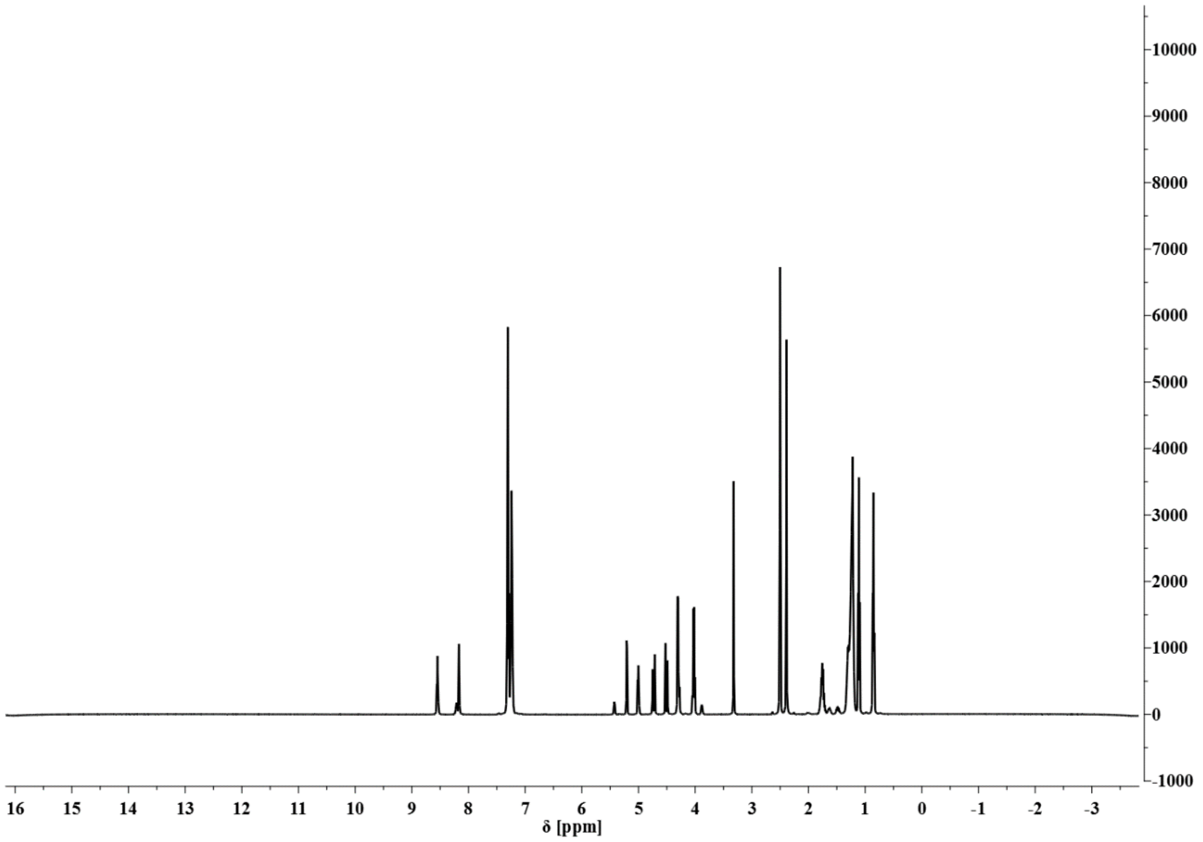
δ [ppm]

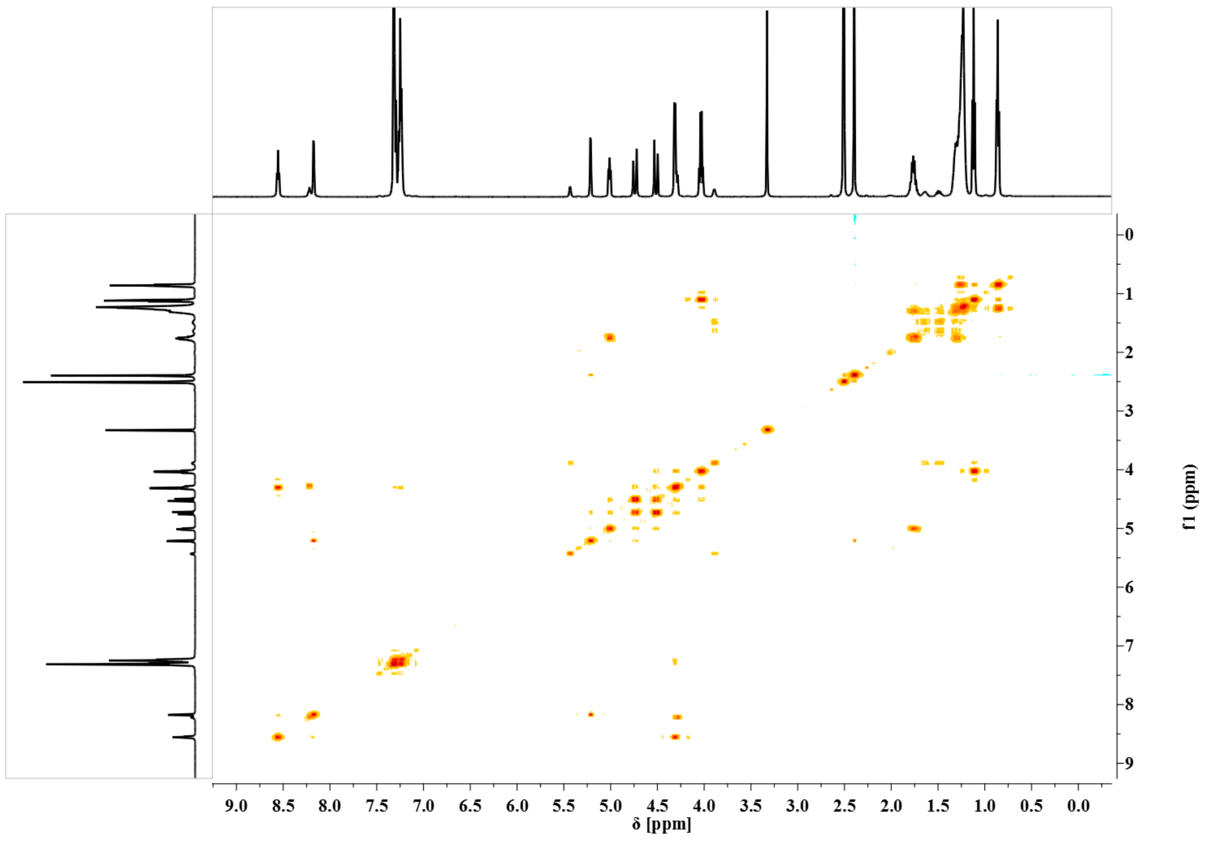
δ [ppm]
f1 (ppm)

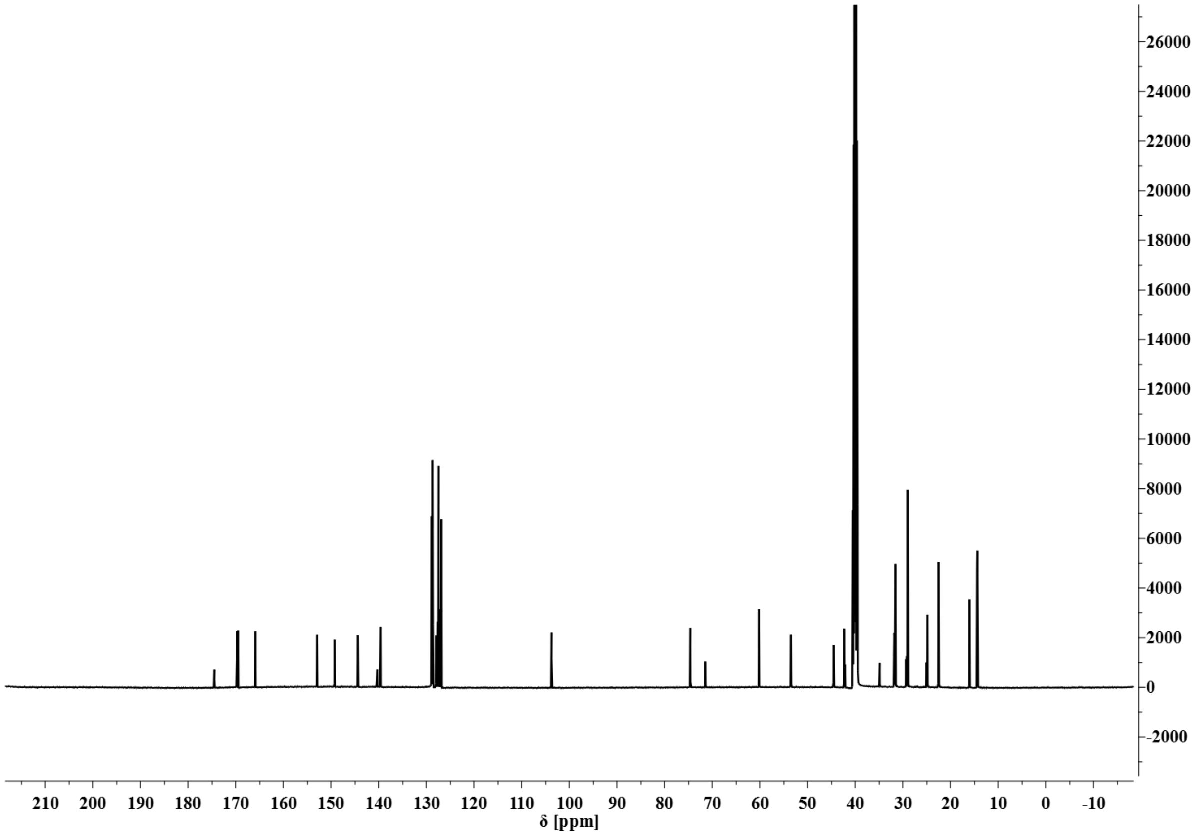

26000
24000
22000
20000
18000
16000
14000
12000
10000
8000
6000
4000
2000
0
-2000
210 200 190 180 170 160 150 140 130 120 110 100 90 80 70 60 50 40 30 20 10 0 -10
δ [ppm]

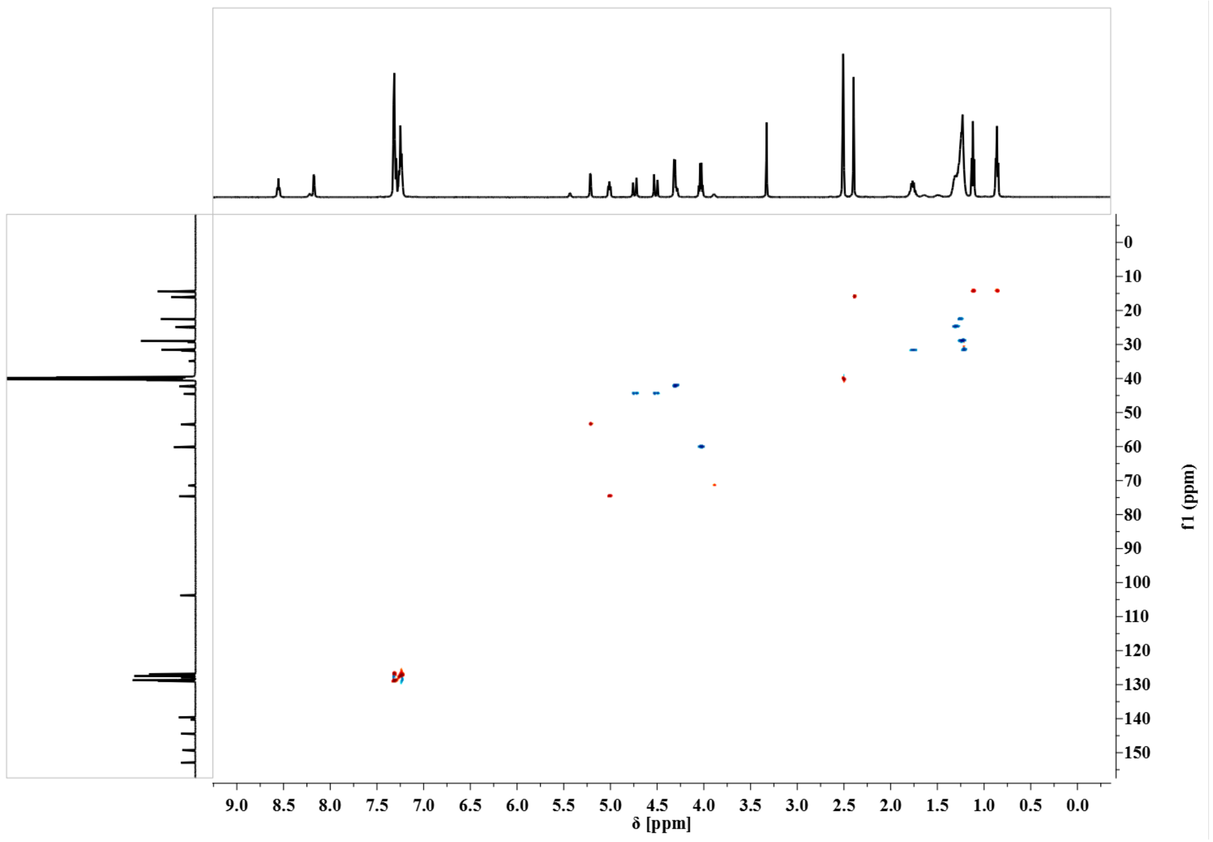

0
10
20
30
40
50
60
70
80
90
100
110
120
130
140
150
f1 (ppm)
9.0 8.5 8.0 7.5 7.0 6.5 6.0 5.5 5.0 4.5 4.0 3.5 3.0 2.5 2.0 1.5 1.0 0.5 0.0
δ [ppm]

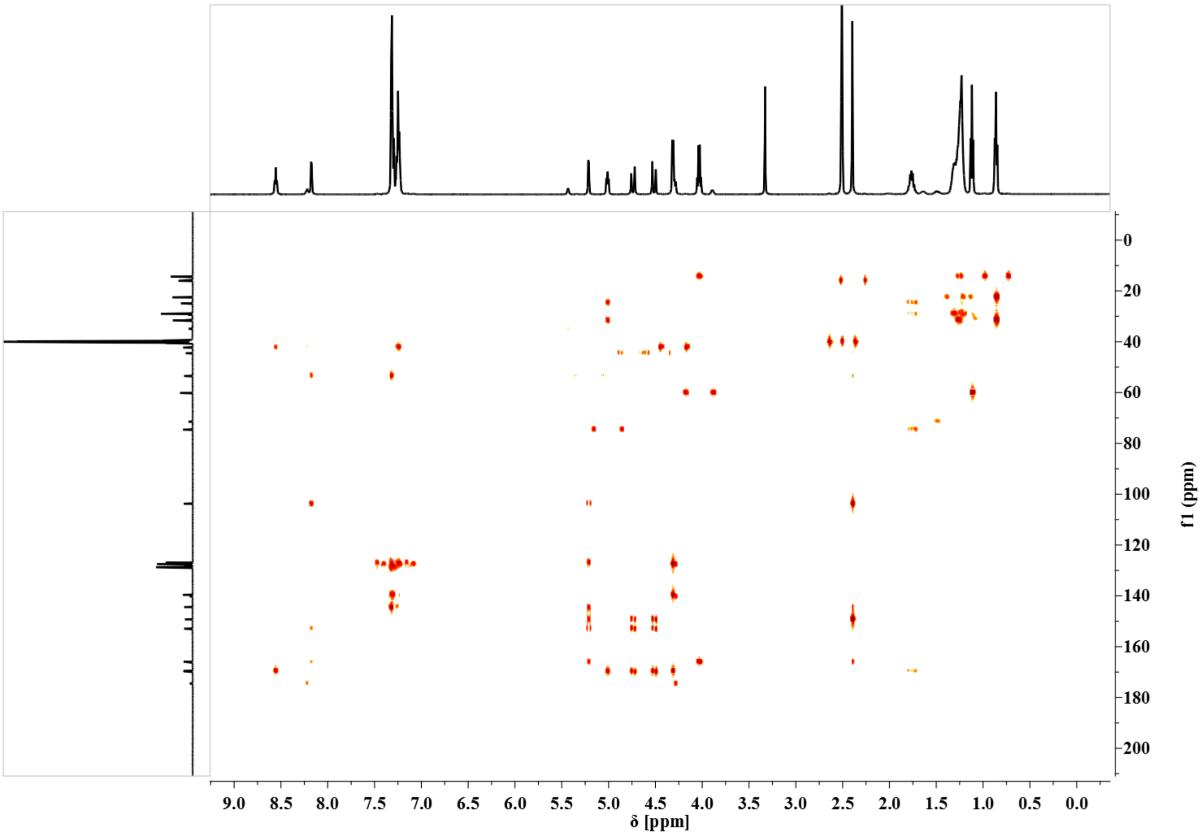

Passerini compound 25 derived from Biginelli acid 15, octanal and 2-morpholinoethyl isocyanide.

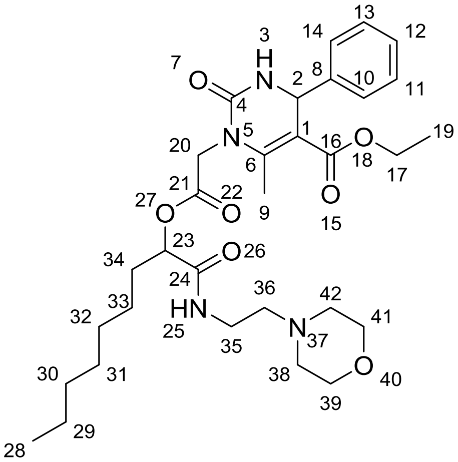

In a tube vial, finely powdered Biginelli acid **15** (302 mg, 949 µmol, 1.00 equiv) was dissolved in 0.3 mL dimethyl sulfoxide and diluted with 1.5 mL dichloromethane. Subsequently, octanal (158 mg, 1.23 mmol, 193 µL, 1.30 equiv) and 2-morpholinoethyl isocyanide (186 mg, 1.32 mmol, 182 µL, 1.40 equiv) were added. The mixture was stirred at room temperature for 3 d. The reaction mixture was evaporated under reduced pressure. The crude product was purified via column chromatography employing silica gel and eluting with a gradual solvent mixture of ethyl acetate and *c*-hexane (0:1 → 1:1) and finally with a solvent mixture of dichloromethane,

methanol and triethylamine (90:5:5). The product containing fractions were dried under reduced pressure to yield a viscous oil. The oil was suspended in 50 mL water, sonicated for 1 h and washed with 30 mL water (this washing was repeated three times). After drying under reduced pressure, the Passerini product **25** was obtained as a yellow solid (222 mg, 372 µmol, 39.2 %).

IR (ATR): ν [cm^{-1}] = 3304.4 (br, ν(N-H)), 2925.8 (w, ν(C-H)), 2854.4 (w, ν(C-H)), 1751.8 (w, ν(C=O)), 1675.6 (vs, ν(C=O)), 1541.3 (w), 1493.8 (m), 1453.9 (s), 1384.2 (m), 1307.5 (w), 1277.7 (w), 1258.5 (w), 1174.9 (vs), 1109.9 (s), 1055.6 (s), 939.9 (w), 862.8 (w), 829.0 (m), 758.1 (s), 697.8 (w), 650.1 (w), 624.4 (w), 511.8 (w), 458.7 (w).

^{1}H NMR (400 MHz, DMSO-d_6): δ [ppm] = 8.16 (dd, J = 21.1, 3.6 Hz, 1 H, NH^3), 7.92 (dt, J = 15.2, 5.7 Hz, 1 H, NH^{25}), 7.40 – 7.11 (m, 5 H, CH_{Ar}), 5.19 (t, J = 3.5 Hz, 1 H, CH^2), 4.93 (dt, J = 10.0, 5.0 Hz, 1 H, CH^{23}), 4.79 – 4.44 (m, 2 H, CH_2^{20}), 4.06 – 3.96 (m, 2 H, CH_2^{17}), 3.58 – 3.45 (m, 6 H, $CH_2^{36+38,42}$), 3.26 – 3.12 (m, 2 H, CH_2^{35}), 2.41 (d, J = 10.5 Hz, 3 H, CH_3^9), 2.39 – 2.30 (m, 2 H, $CH_2^{39,41}$), 1.76 – 1.63 (m, 2 H, CH_2^{34}), 1.34 – 1.15 (m, 12 H, CH_2), 1.13 – 1.06 (m, 3 H, CH_3^{19}), 0.85 (t, J = 6.8 Hz, 3 H, CH_3^{28}).

^{13}C NMR (101 MHz, DMSO-d_6): δ [ppm] = 169.13 (s, CO_2R^{20}), 169.04 (s, CO_2R^{20}), 168.78 (s, $CONR^{24}$), 168.77 (s, $CONR^{24}$), 165.44 (s, CO_2R^{16}), 165.42 (s, CO_2R^{16}), 152.48 (s, CO^4), 152.24 (s, CO^4), 148.73 (s, C^6), 148.59 (s, C^6), 144.05 (s, $C_{Ar}{}^8$), 143.89 (s, $C_{Ar}{}^8$), 128.40 (s, CH_{Ar}), 128.32 (s, CH_{Ar}), 127.42 (s, CH_{Ar}), 126.65 (s, CH_{Ar}), 126.51 (s, CH_{Ar}), 126.43 (s, CH_{Ar}), 103.34 (s, C^1), 103.27 (s, C^1), 74.07 (s, CH^{23}), 74.03 (s, CH^{23}), 66.16 (s, $CH_2^{36+38,42}$), 59.70 (s, CH_2^{17}), 57.12 (s, $CH_2^{39,41}$), 53.19 (s, CH^2), 44.09 (s, CH_2^{20}), 43.88 (s, CH_2^{20}), 35.73 (s, CH_2^{35}), 31.37 (s, CH_2), 31.30 (s, CH_2), 31.12 (s, CH_2), 28.56 (s, CH_2), 28.50 (s, CH_2), 28.48 (s, CH_2), 24.29 (s, CH_2), 24.27 (s, CH_2), 22.05 (s, CH_2), 15.60 (s, CH_3^9), 15.59 (s, CH_3^9), 13.94 (s, CH_3^{28}), 13.90 (s, CH_3^{28}).

FAB – MS [m/z] (relative intensity): 587.3 (100%) $[M + H]^+$, 541.3 (15%) $[M – C_2H_5O]^+$.

HRMS – FAB [m/z]: $[M + H]^+$ calculated for $^{12}C_{31}{}^1H_{47}{}^{16}O_7{}^{14}N_4$, 587.3439; found, 587.3441; Δ = 0.16 mmu.

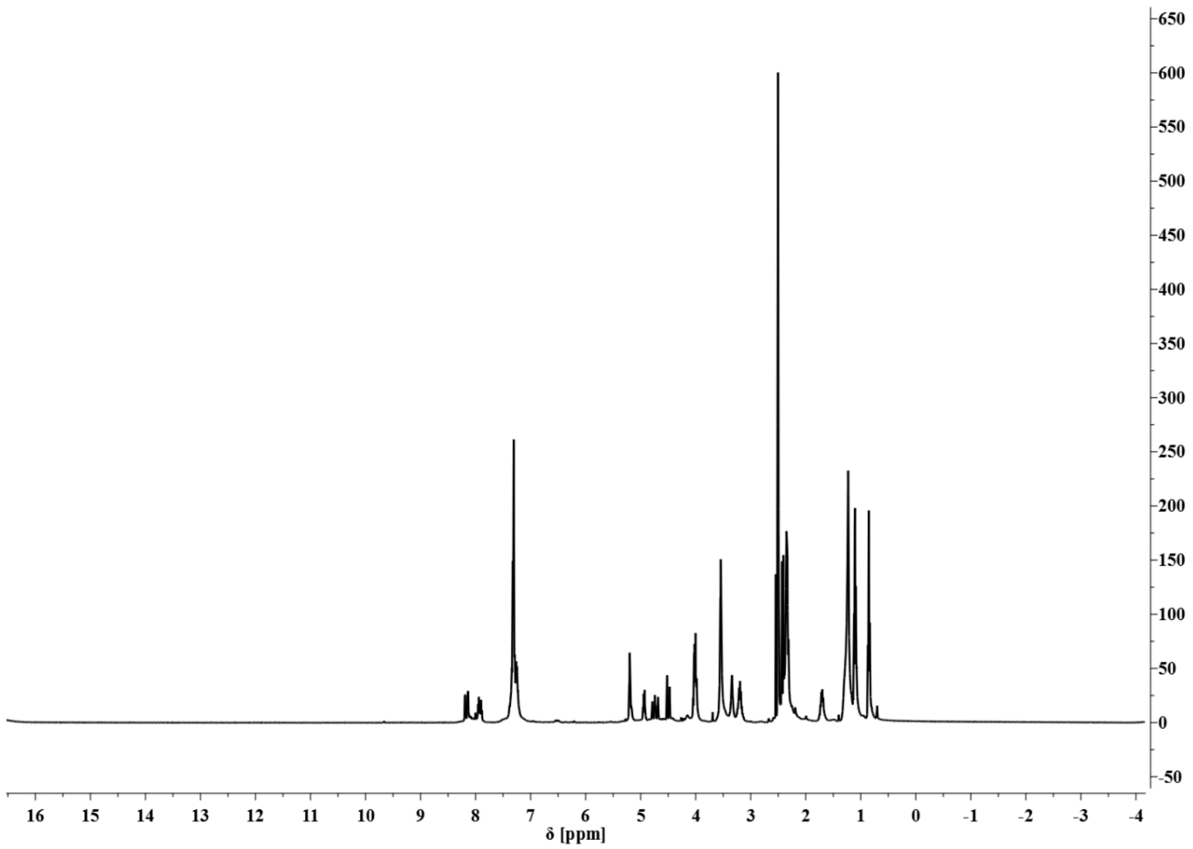
δ [ppm]

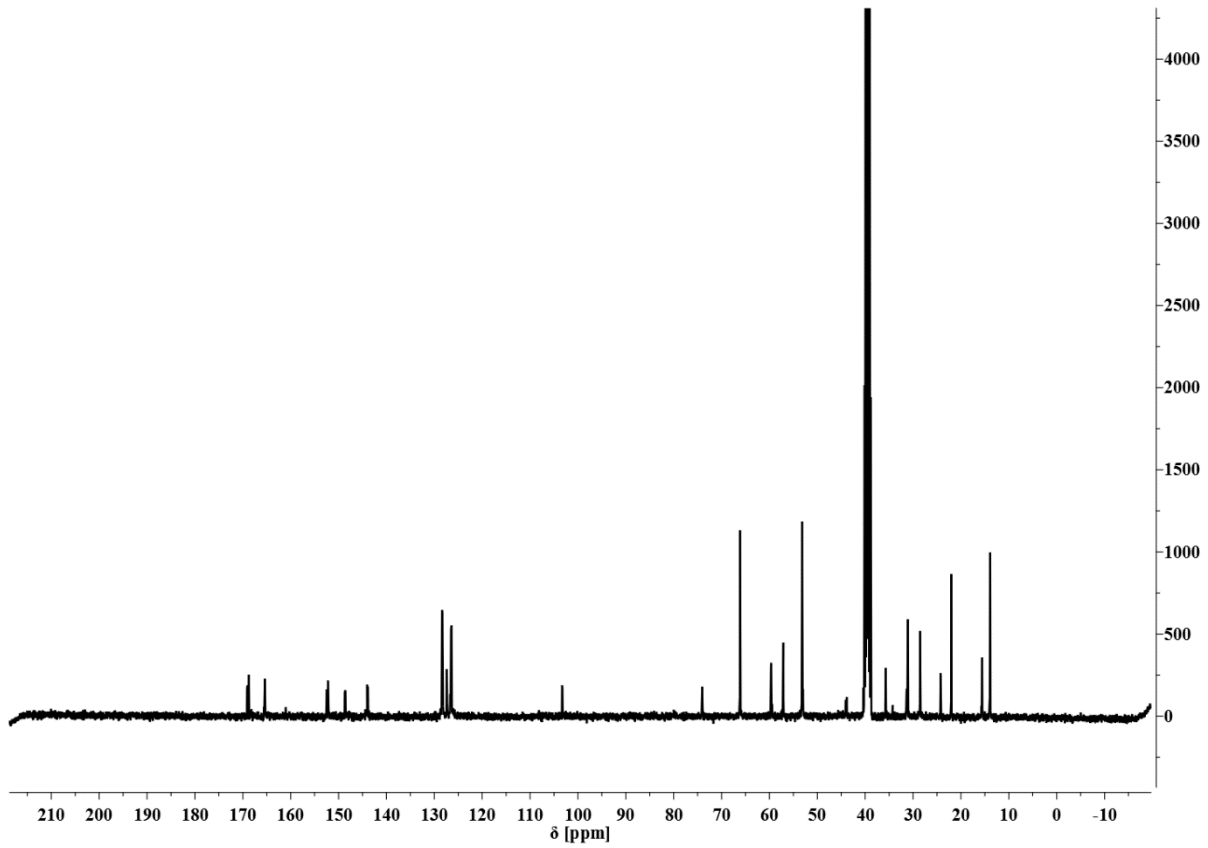
δ [ppm]

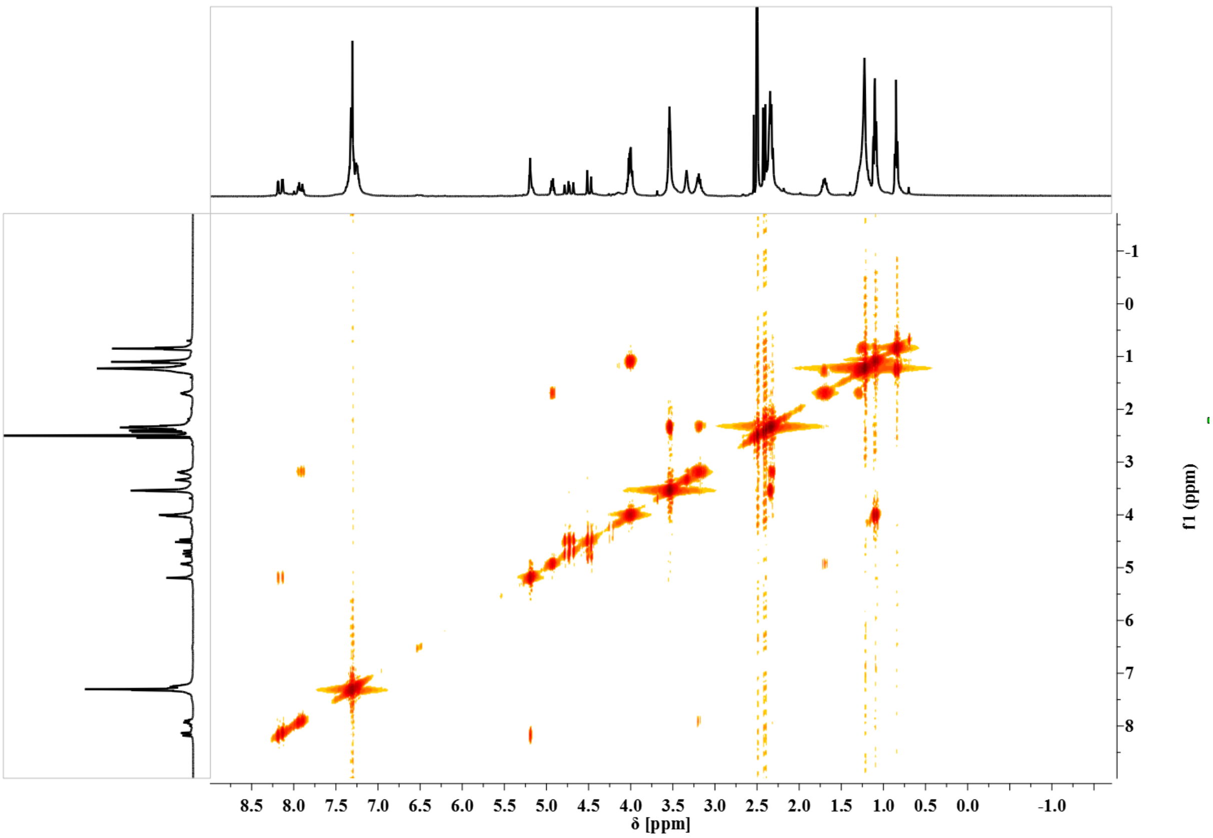

8.5 8.0 7.5 7.0 6.5 6.0 5.5 5.0 4.5 4.0 3.5 3.0 2.5 2.0 1.5 1.0 0.5 0.0 -1.0
δ [ppm]
-1 0 1 2 3 4 5 6 7 8
f1 (ppm)

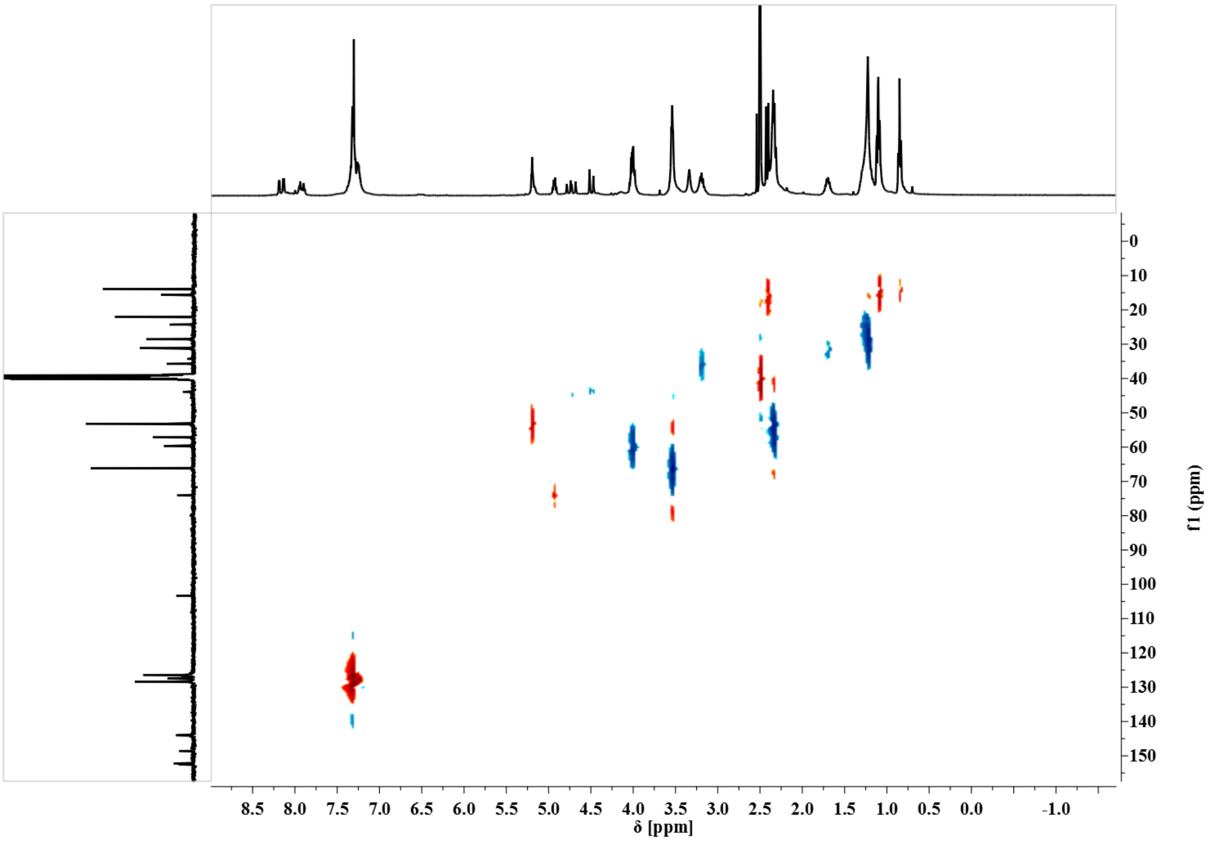

8.5 8.0 7.5 7.0 6.5 6.0 5.5 5.0 4.5 4.0 3.5 3.0 2.5 2.0 1.5 1.0 0.5 0.0 -1.0
δ [ppm]
0 10 20 30 40 50 60 70 80 90 100 110 120 130 140 150
f1 (ppm)

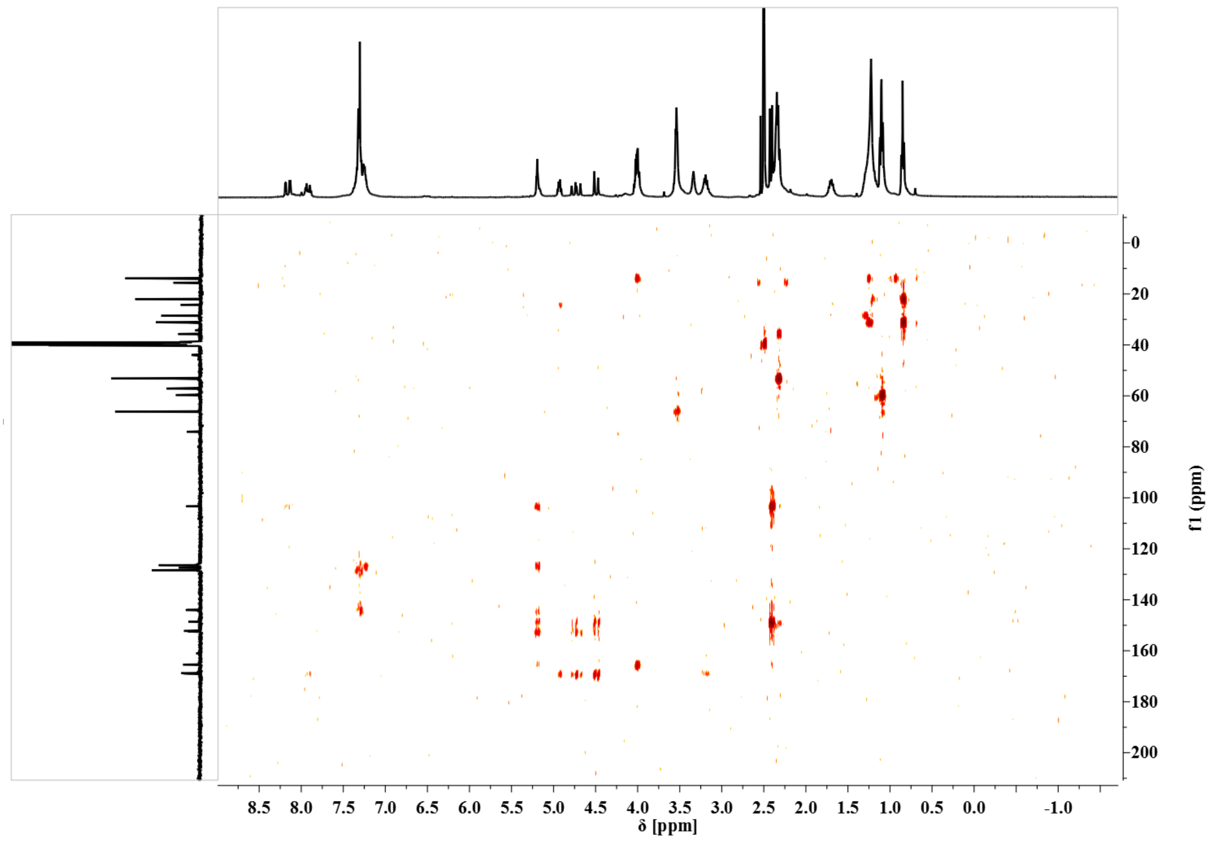

Passerini compound 26 derived from Biginelli acid 15, 2-ethylbutanal and 1-pentyl isocyanide

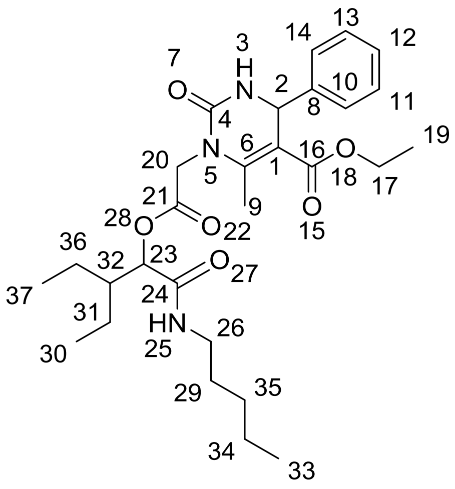

In a tube vial, finely powdered Biginelli-acid **15** (300 mg, 942 µmol, 1.00 equiv) was dissolved in 0.3 mL dimethyl sulfoxide and diluted with 1.0 mL dichloromethane. Subsequently, 2-ethylbutanal (200 mg, 2.00 mmol, 246 µL, 2.10 equiv) and 1-pentyl isocyanide (120 mg, 1.23 mmol, 155 µL, 1.30 equiv) were added. The mixture was stirred at room temperature for 3 d. The crude product was purified via column chromatography employing silica gel and eluting with a gradual solvent mixture of diethyl ether and *c*-hexane (0:1 → 5:1). The Passerini product **26** was obtained as a colorless solid (381 mg, 739 µmol, 78.5%).

IR (ATR): ν [cm^{-1}] = 3304.9 (br, ν(N-H)), 2958.8 (w, ν(C-H), 2930.1 (w, ν(C-H), 2872.3 (w, ν(C-H), 1752.2 (s, ν(C=O)), 1681.1 (vs, ν(C=O)), 1535.4 (m), 1455.7 (m), 1383.7 (m), 1309.7 (w), 1278.3 (w), 1177.3 (vs), 1104.9 (m), 1055.6 (m), 940.8 (w), 830.3 (w), 760.0 (w), 697.6 (w), 651.4 (w).

^{1}H NMR (400 MHz, DMSO-d_6): δ [ppm] = 8.19 (dd, J = 32.9, 3.7 Hz, 1 H, NH^{3}), 7.93 (dt, J = 33.8, 5.7 Hz, 1 H, NH^{25}), 7.34 – 7.22 (m, 5 H, CH_{Ar}), 5.20 (dd, J = 5.6, 3.8 Hz, 1 H, CH^{2}), 4.99 (dd, J = 17.2, 3.9 Hz, 1 H, CH^{23}), 4.75 – 4.45 (m, 2 H, CH_2^{20}), 4.02 (p, J = 7.0 Hz, 2 H, CH_2^{17}), 3.14 – 2.99 (m, 2 H, CH_2^{26}), 2.43 (d, J = 25.0 Hz. 3 H, CH_3^{9}), 1.78 – 1.66 (m, 1 H, CH^{32}), 1.45 – 1.32 (m, 4 H, CH_2), 1.33 – 1.15 (m, 6 H, CH_2), 1.10 (td, J = 7.1, 4.6 Hz, 3 H, CH_3^{19}), 0.89 – 0.79 (m, 9 H, $CH_3^{30,37}$).

^{13}C NMR (101 MHz, DMSO-d_6): δ [ppm] = 169.24 (s, CO_2R^{21}), 169.21 (s, CO_2R^{21}), 168.37 (s, $CONR^{24}$), 165.50 (s, CO_2R^{16}), 165.44 (s, CO_2R^{16}), 152.78 (s, CO^{4}), 152.37 (s, CO^{4}), 148.78 (s, C^{6}), 148.72 (s, C^{6}), 144.00 (s, C_{Ar}^{8}), 143.78 (s, C_{Ar}^{8}), 128.45 (s, CH_{Ar}), 128.39 (s, CH_{Ar}), 127.49 (s, CH_{Ar}), 127.45 (s, CH_{Ar}), 126.51 (s, CH_{Ar}), 126.40 (s, CH_{Ar}), 103.42 (s, C^{1}), 103.21 (s, C^{1}), 75.13 (s, CH^{23}), 75.02 (s, CH^{23}), 59.76 (s, CH_2^{17}), 59.69 (s, CH_2^{17}), 53.16 (s, CH^{2}), 52.92 (s, CH^{2}), 44.34 (s, CH_2^{20}), 44.12 (s, CH_2^{20}), 42.53 (s, CH^{32}), 42.51 (s, CH^{32}), 38.34 (s, CH_2^{26}), 28.62 (s, CH_2), 28.57 (s, CH_2), 28.48 (s, CH_2), 28.46 (s, CH_2), 21.80 (s, CH_2), 21.44 (s, CH_2), 21.26 (s, CH_2), 15.65 (s, CH_3^{9}), 15.62 (s, CH_3^{9}), 13.97 (s, CH_3^{19}), 13.91 (s, CH_3^{19}), 11.38 (s, $CH_3^{30,37}$), 11.33 (s, $CH_3^{30,37}$), 11.26 (s, $CH_3^{30,37}$).

FAB – MS [m/z] (relative intensity): 516.3 (100%) $[M + H]^+$, 470.2 (30%), $[M – C_2H_5O]^+$, 317.1 (35%) [Fragment A]$^+$, 301.1 (35%) [Fragment A – O]$^+$, 259.1 (45%) [Fragment C]$^+$, 198.2 (30%) [Fragment B]$^+$.

HRMS – FAB [m/z]: $[M + H]^+$ calculated for $^{12}C_{28}{}^{1}H_{42}{}^{16}O_6{}^{14}N_3$, 516.3068; found, 516.3069; Δ = 0.07 mmu.

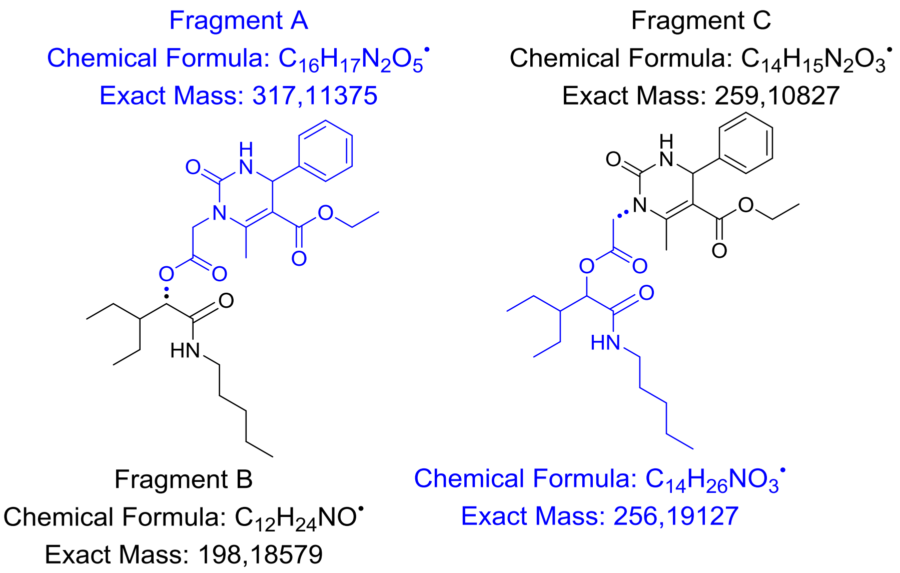
Fragment A
Chemical Formula: C16H17N2O5•
Exact Mass: 317,11375
Fragment C
Chemical Formula: C14H15N2O3•
Exact Mass: 259,10827
Fragment B
Chemical Formula: C12H24NO•
Exact Mass: 198,18579
Chemical Formula: C14H26NO3•
Exact Mass: 256,19127

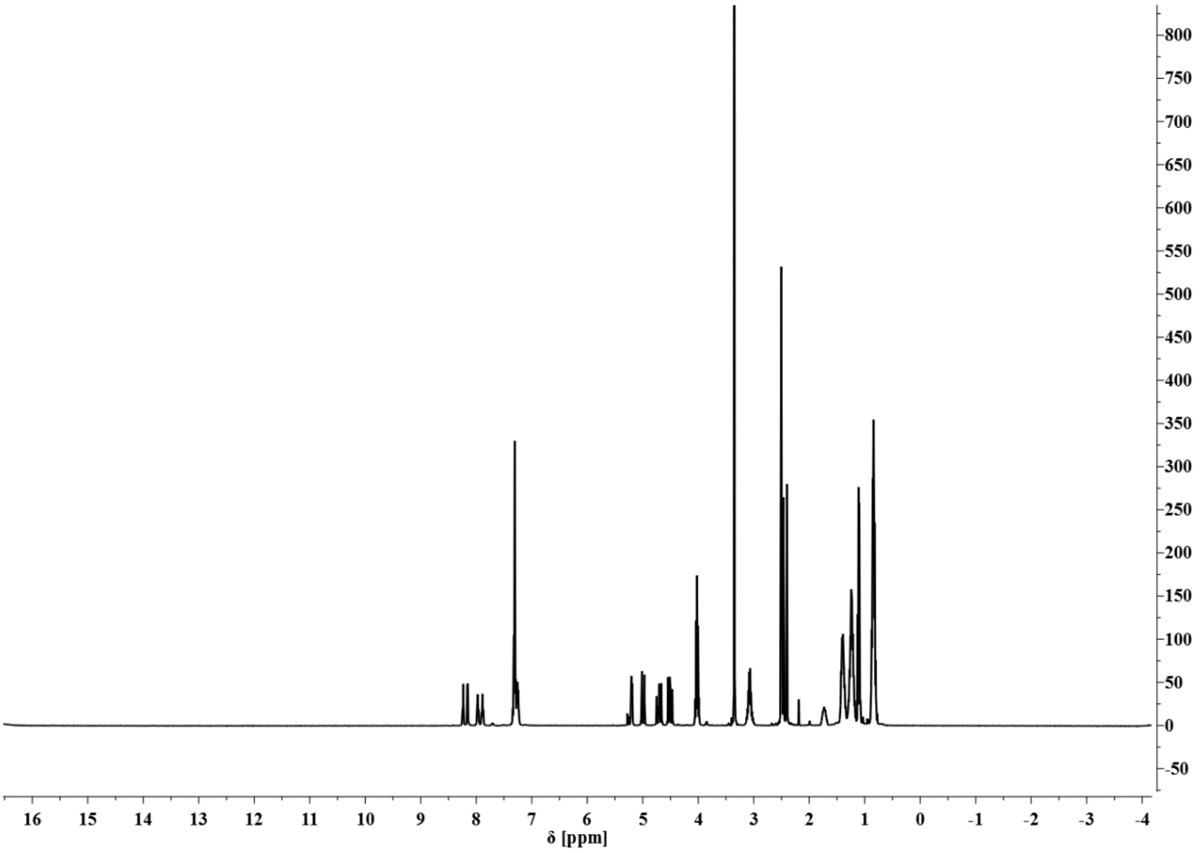
δ [ppm]

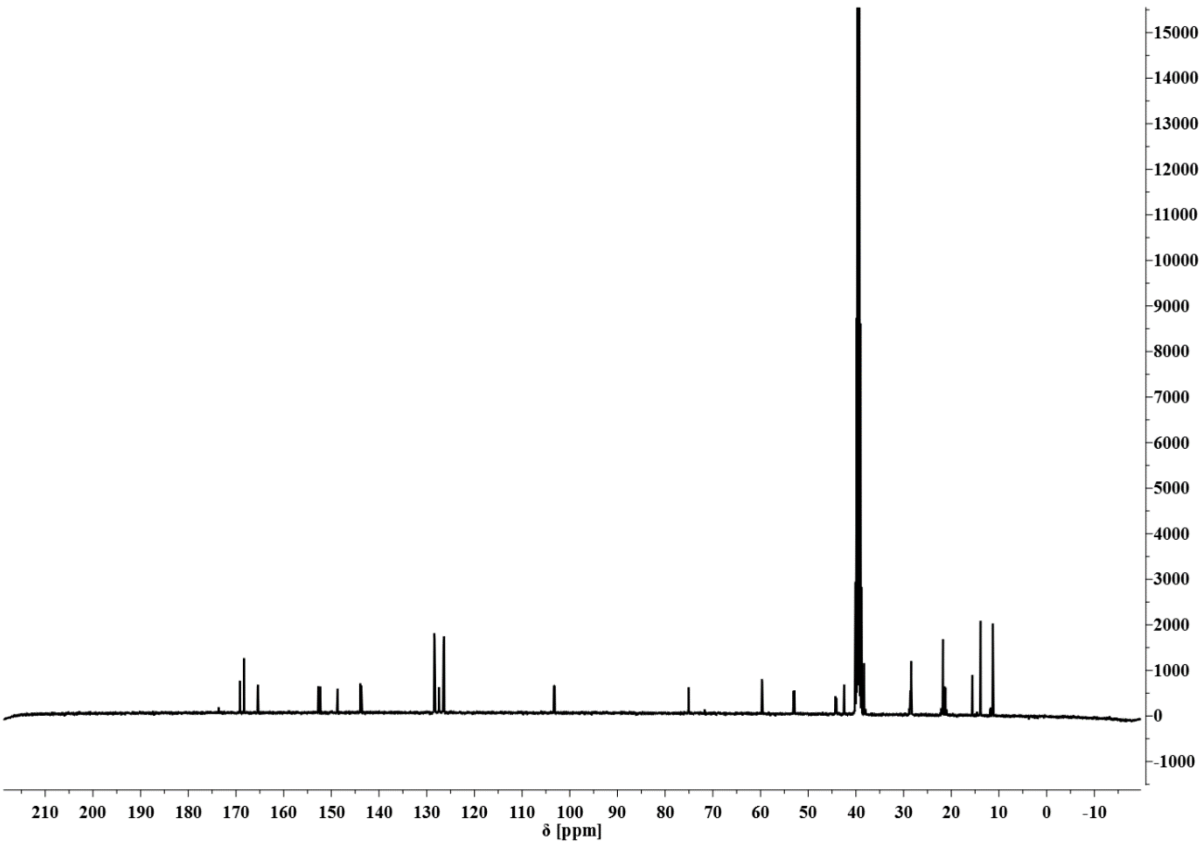

15000
14000
13000
12000
11000
10000
9000
8000
7000
6000
5000
4000
3000
2000
1000
0
-1000
210 200 190 180 170 160 150 140 130 120 110 100 90 80 70 60 50 40 30 20 10 0 -10
δ [ppm]

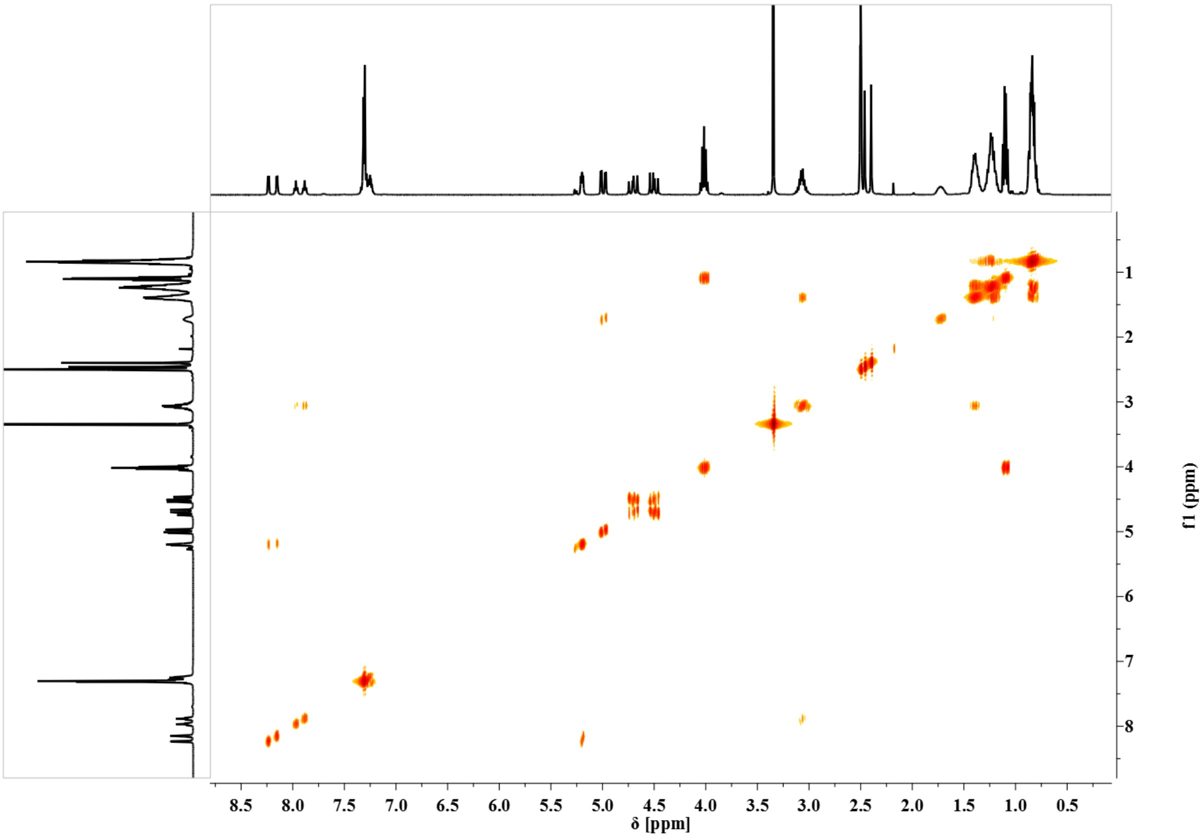

8.5 8.0 7.5 7.0 6.5 6.0 5.5 5.0 4.5 4.0 3.5 3.0 2.5 2.0 1.5 1.0 0.5
δ [ppm]
1 2 3 4 5 6 7 8
f1 (ppm)

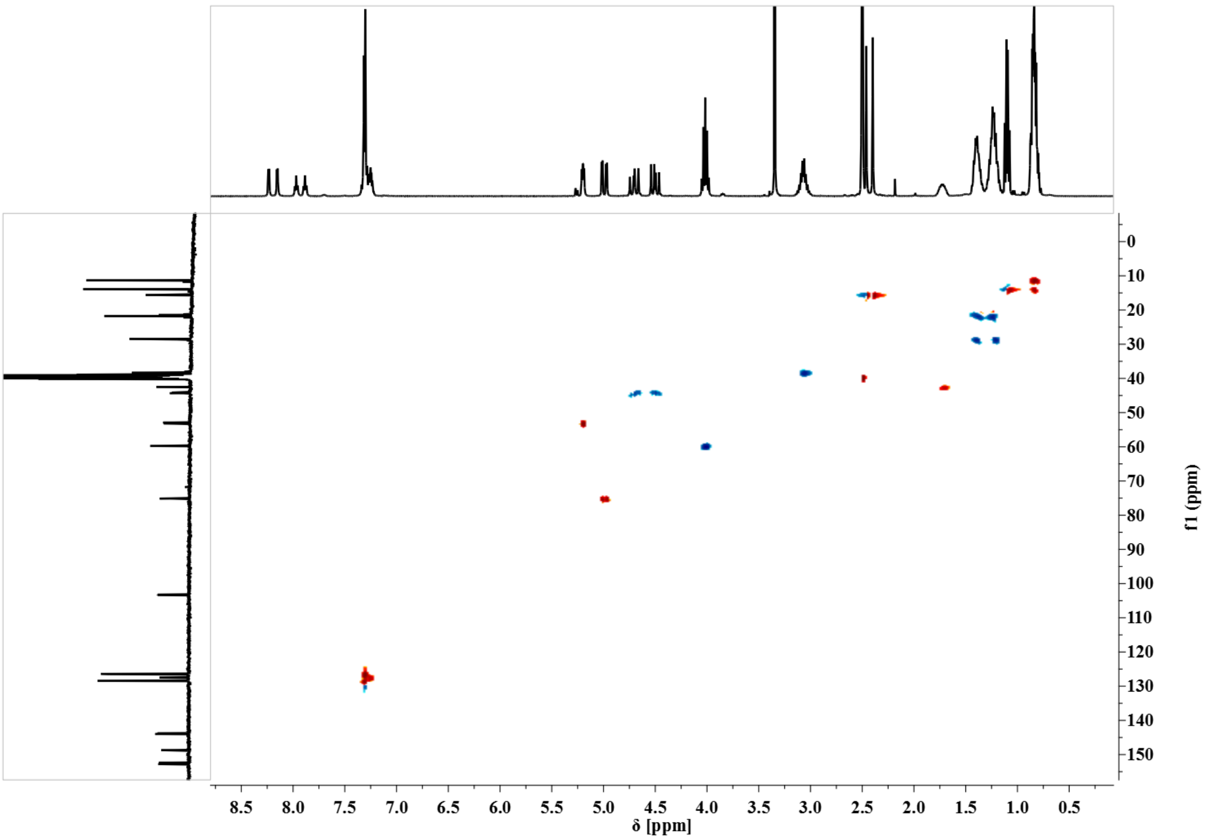

f1 (ppm)
δ [ppm]

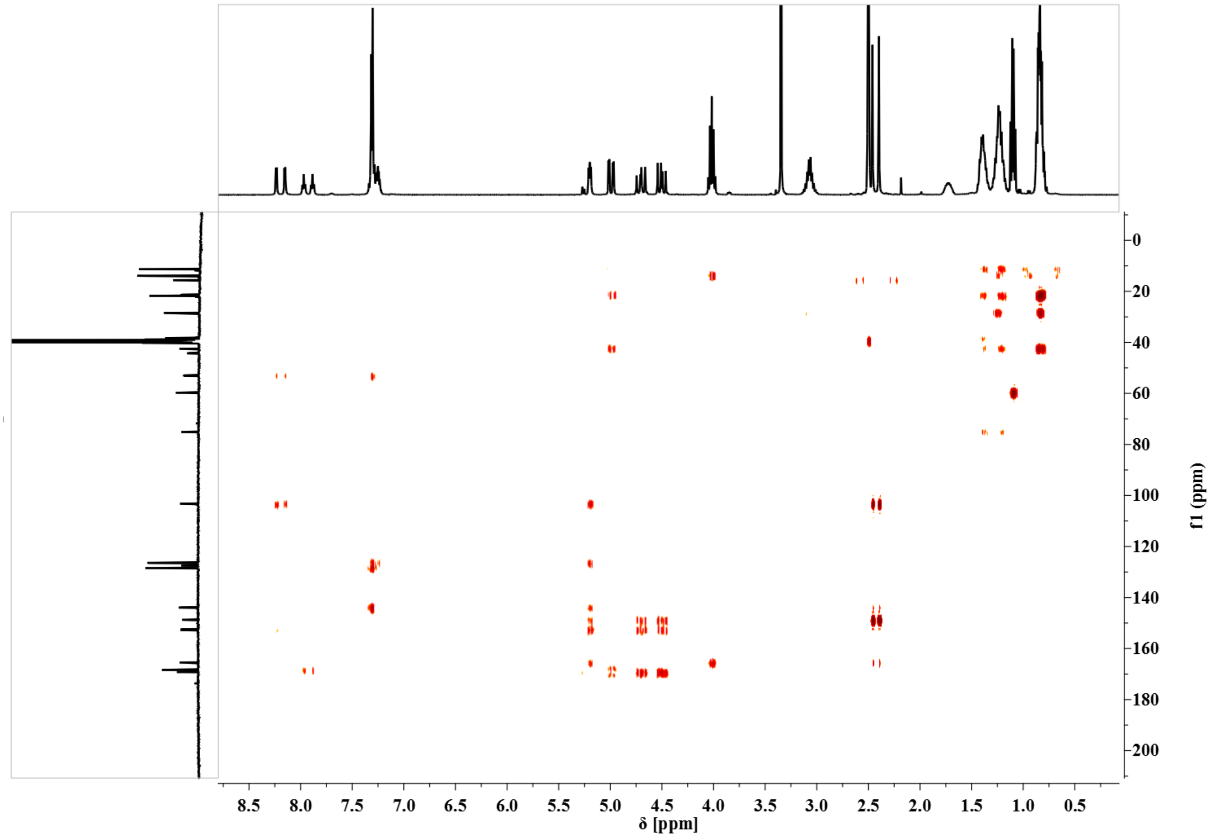

f1 (ppm)
δ [ppm]

Biginelli-Passerini product 27 prepared in one pot procedure from 4-fluorobenzaldehyde, *N*-carbamoylglycine, ethyl acetoacetate and 1-pentylisocyanide

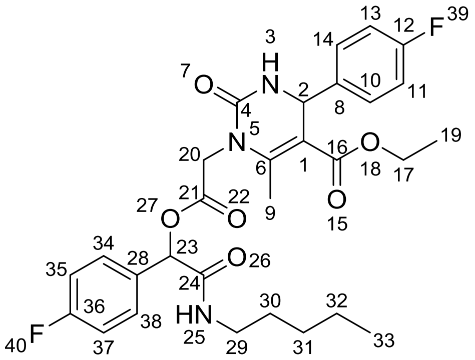

In a tube vial, finely powdered *N*-carbamoylglycine (500 mg, 4.23 mmol, 1.00 equiv), 4-fourobenzaldehyde (1.57 g, 12.7 mmol, 3.00 equiv) and ethyl acetoacetate (551 mg, 4.23 mmol, 1.00 equiv) were stirred at 110 °C for 24 h and subsequently at 80 °C for another 24 h. Subsequently, the crude reaction mixture was cooled to ambient temperature and diluted with 3 mL dichloromethane. Pentlyisocyanide (617 mg, 6.35 mmol, 1.50 equiv) was added while stirring. The resulting mixture was stirred for 3 d at room temperature. The reaction mixture was dried under reduced pressure and purified via column chromatography employing silica gel and eluting with a gradual solvent mixture of ethyl acetate and *c*-hexane (1:5 → 1:0) to yield the Biginelli-Passerini one pot product as a colorless solid (972 mg, 1.74 mmol, 41.1%). Note: impure fractions can be recrystallized by precipitation from ethyl acetate in *c*-hexane and evaporation of the solvents overnight in an open flask.

R_f = 0.51 in ethyl acetate/*c*-hexane (2:1).

IR (ATR): ν [cm^{-1}] = 3287.9 (br, ν(N-H)), 3097.3 (br, ν(N-H)), 2931.7 (w, ν(C-H)), 2860.3 (w, ν(C-H)), 1737.5 (m, ν(C=O)), 1682.1 (vs, ν(C=O)), 1659.4 (vs), 1621.6 (s), 1602.6 (s), 1556.4 (s), 1508.3 (vs), 1449.9 (m), 1411.3 (m), 1369.2 (m), 1308.4 (m), 1277.5 (m), 1256.0 (m), 1188.6 (vs), 1157.4 (s), 1098.5 (m), 1055.2 (m), 1013.6 (w), 940.1 (w), 806.4 (w), 758.7 (w), 648.2 (w), 579.8 (w), 515.6 (w).

^{1}H NMR (500 MHz, DMSO-d_6): δ [ppm] = 8.27 (q, J = 5.6 Hz, 1 H, NH^{25}), 8.16 (dd, J = 16.4, 3.4 Hz, 1 H, NH^{3}), 7.54 – 7.50 (m, 2 H, CH$_{Ar}$), 7.35 (ddd, J = 31.1, 8.3, 5.6 Hz, 2 H, CH$_{Ar}$), 7.23 (td, J = 8.8, 3.7 Hz, 2 H, CH$_{Ar}$), 7.17 – 7.06 (m, 2 H, CH$_{Ar}$), 5.92 (d, J = 10.2 Hz, 1 H, CH^{23}), 5.20 (t, J = 3.7 Hz, 1 H, CH^{2}), 4.92 – 4.52 (m, 2 H, CH$_2^{20}$), 4.07 – 3.95 (m, 2 H, CH$_2^{17}$), 3.10 – 2.98 (m, 2 H, CH$_2^{29}$), 2.43 (d, J = 10.8 Hz, 3 H, CH$_3^{9}$), 1.43 – 1.33 (m, 2 H, CH$_2$), 1.28 – 1.05 (m, 7 H, CH$_2$ + CH$_3^{19}$), 0.87 – 0.74 (m, 3 H, CH$_3^{33}$).

^{13}C NMR (126 MHz, DMSO-d_6): δ [ppm] = 169.00 (s, CO$_2$R^{21}), 168.96 (s, CO$_2$R^{21}), 167.29 (s, CONR^{24}), 167.26 (s, CONR^{24}), 165.37 (s, CO$_2$R^{16}), 165.36 (s, CO$_2$R^{16}), 162.25 (d, J = 244.9

Hz, CF_{Ar}), 162.23 (d, J = 245.1 Hz, CF_{Ar}), 161.45 (d, J = 243.3 Hz, CF_{Ar}), 161.42 (d, J = 243.4 Hz, CF_{Ar}), 152.16 (s, CO^{4}), 152.01 (s, CO^{4}), 148.92 (s, C^{6}), 148.73 (s, C^{6}), 140.36 (d, J = 2.9 Hz, C_{Ar}^{8}), 140.19 (d, J = 3.0 Hz, C_{Ar}^{8}), 131.85 (d, J = 2.9 Hz, C_{Ar}^{28}), 131.76 (d, J = 2.9 Hz, C_{Ar}^{28}), 129.63 (s, CH_{Ar}), 129.56 (s, CH_{Ar}), 129.48 (s, CH_{Ar}), 128.69 (s, CH_{Ar}), 128.62 (s, CH_{Ar}), 128.54 (s, CH_{Ar}), 128.47 (s, CH_{Ar}), 128.43 (s, CH_{Ar}), 128.37 (s, CH_{Ar}), 115.43 (s, CH_{Ar}), 115.25 (s, CH_{Ar}), 115.18 (s, CH_{Ar}), 115.16 (s, CH_{Ar}), 115.01 (s, CH_{Ar}), 114.99 (s, CH_{Ar}), 114.71 (s, CH_{Ar}), 114.54 (s, CH_{Ar}), 103.28 (s, C^{1}), 103.16 (s, C^{1}), 75.12 (s, CH^{23}), 75.00 (s, CH^{23}), 59.77 (s, CH_2^{17}), 52.66 (s, CH^{2}), 52.44 (s, CH^{2}), 43.91 (s, CH_2^{20}), 43.74 (s, CH_2^{20}), 38.39 (s, CH_2^{29}), 38.36 (s, CH_2^{29}), 28.79 (s, CH_2), 28.56 (s, , CH_2), 28.54 (s, CH_2), 28.49 (s, CH_2), 28.30 (s, CH_2), 21.73 (s, CH_2), 15.61 (s, CH_3^{9}), 13.97 (s, CH_3^{19}), 13.95 (s, CH_3^{19}), 13.84 (s, CH_3^{33}), 13.83 (s, CH_3^{33}).

^{19}F NMR (377 MHz, DMSO-d_6): δ [ppm] = -117.49 (s, CF_{Ar}), -117.50 (s, CF_{Ar}), -119.43 (s, CF_{Ar}), -119.49 (s, CF_{Ar}).

FAB – MS [m/z] (relative intensity): 558.3 (80%) $[M + H]^+$, 512.2 (40%) $[M - C_2H_5O]^+$, 335.1 (100%) $[\text{Fragment A}]^+$, 222.1 (30%) $[\text{Fragment B}]^+$, 136.0 (70 %) $[\text{Fragment B} - C_5H_{12}N]^+$.

HRMS – FAB [m/z]: $[M + H]^+$ calculated for $^{12}C_{29}{}^{1}H_{34}{}^{16}O_6{}^{14}N_3{}^{19}F_2$, 558.2410; found, 558.2411; Δ = 0.11 mmu.

Fragment A

Chemical Formula: $C_{16}H_{16}FN_2O_5^{\bullet}$

Exact Mass: 335,10432

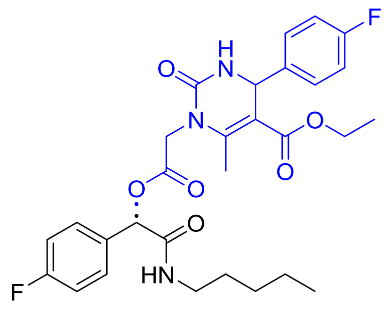

Fragment B

Chemical Formula: $C_{13}H_{17}FNO^{\bullet}$

Exact Mass: 222,12942

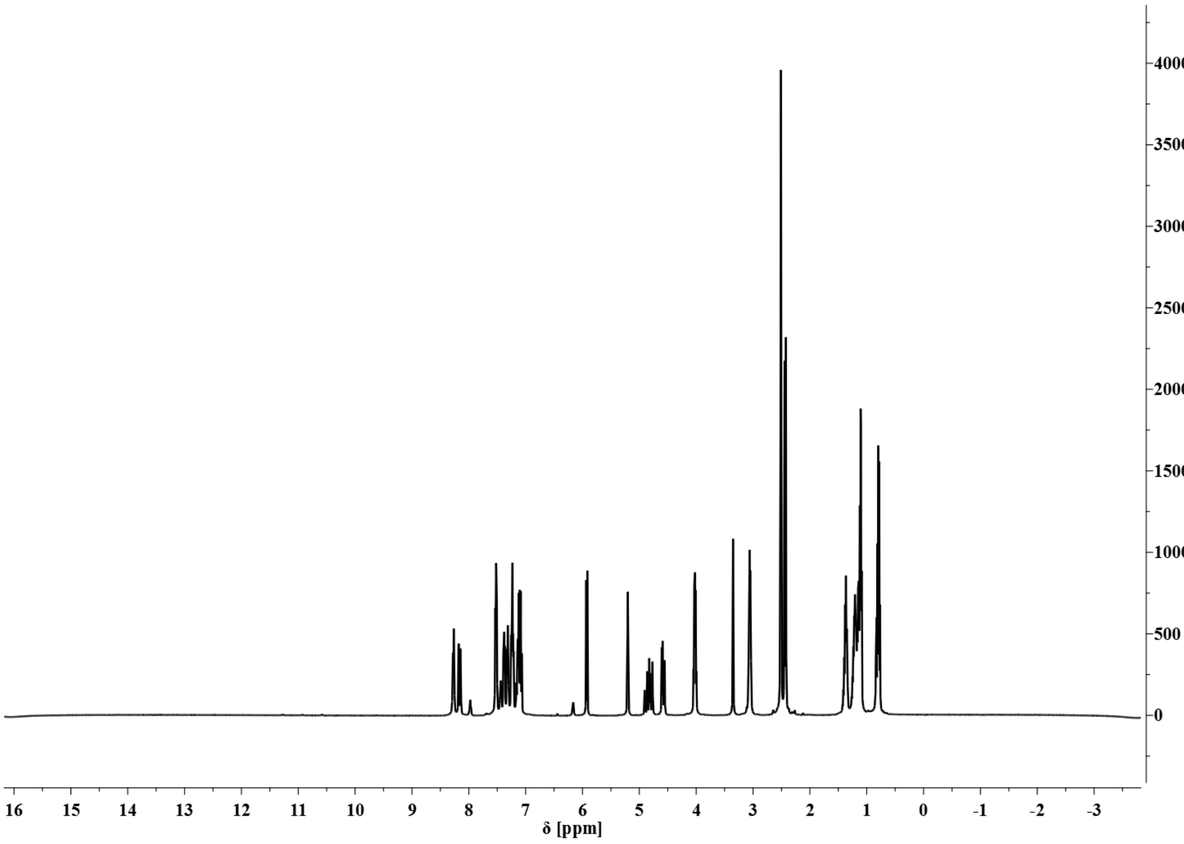
δ [ppm]

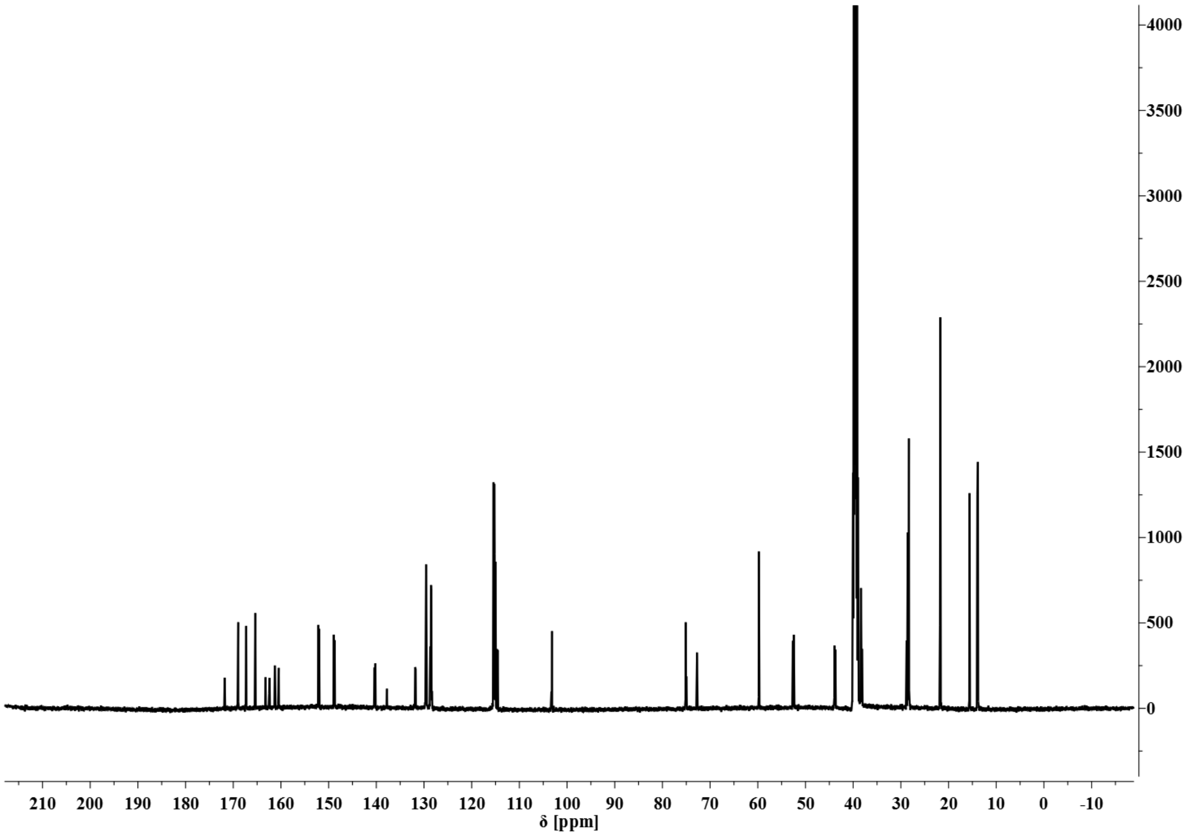
δ [ppm]

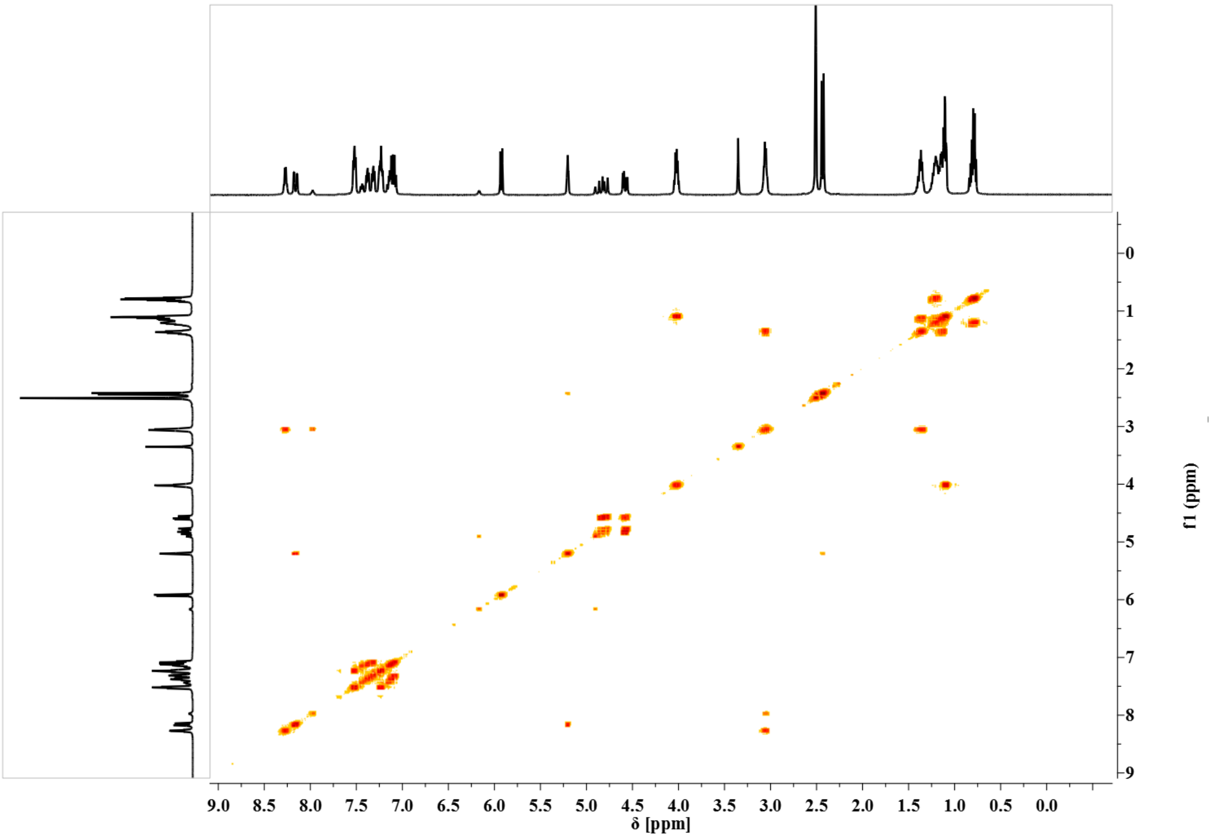
δ [ppm]
f1 (ppm)

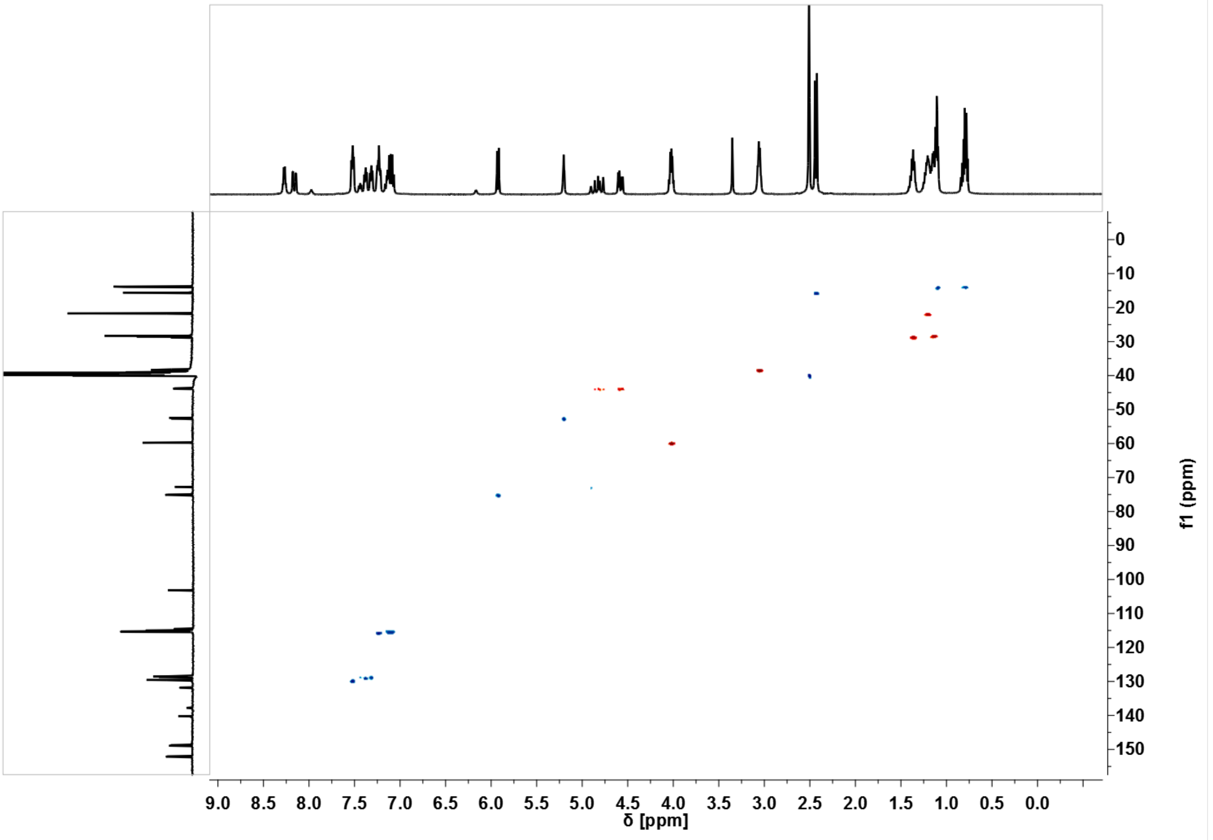
δ [ppm]
f1 (ppm)

References

1. W. C. Still, M. Kahn, A. Mitra, *J. Org. Chem.* **1978**, *43*, 2923–2925.
2. A. Shaabani, A. Bazgir, S. Arab-Ameri, *Phosphorus. Sulfur. Silicon Relat. Elem.* **2004**, *179*, 2169–2175.
3. B. Desai, D. Dallinger, C. O. Kappe, *Tetrahedron* **2006**, *62*, 4651–4664.
4. E. N. Ostapchuk, A. S. Plaskon, O. O. Grygorenko, A. A. Tolmachev, S. V. Ryabukhin, *J. Heterocycl. Chem.* **2013**, *50*, 1299–1303.
5. N. A. Liberto, S. De Paiva Silva, Â. De Fátima, S. A. Fernandes, *Tetrahedron* **2013**, *69*, 8245–8249.
